# Trends and Patterns in Gastrointestinal Cancer Burden in Asia, 1990–2023: Evidence from the Global Burden of Disease Study 2023

**DOI:** 10.3390/cancers18213535

**Published:** 2026-11-03

**Authors:** Lanwei Guo, Chenxin Zhu, Jiani Yuan, Lin Cai, Huamei Luan, Hongda Chen, Yan Zheng, Yanyan Liu

**Affiliations:** 1 Clinical Trials Center, The Affiliated Cancer Hospital of Zhengzhou University & Henan Cancer Hospital, Zhengzhou 450008, China; chxinzhu@gs.zzu.edu.cn (C.Z.); jnyuan@gs.zzu.edu.cn (J.Y.); tingting2025@gs.zzu.edu.cn (L.C.); lhm970805@gs.zzu.edu.cn (H.L.); zlyysunnyzheng@zzu.edu.cn (Y.Z.); yyliu@zzu.edu.cn (Y.L.); 2 Department of Epidemiology, School of Public Health, Zhengzhou University, Zhengzhou 450001, China; 3 Center for Prevention and Early Intervention, National Infrastructures for Translational Medicine, Institute of Clinical Medicine, Peking Union Medical College Hospital, Chinese Academy of Medical Sciences & Peking Union Medical College, Beijing 100730, China; chenhongda@pumch.cn

**Keywords:** Asia, gastrointestinal cancers, burden, trends

## Abstract

Background: Gastrointestinal cancers are among the leading causes of cancer incidence and mortality worldwide and represent a major contributor to the cancer burden in Asia. This study aims to assess the burden of six major gastrointestinal cancers in Asia from 1990 to 2023. Methods: Based on the Global Burden of Disease 2023 data, we analyzed the incidence, mortality, and disability-adjusted life years (DALYs) of six major gastrointestinal cancers—esophageal cancer (EC), stomach cancer (SC), colon and rectum cancer (CRC), liver cancer (LC), pancreatic cancer (PC), and gallbladder and biliary tract cancer (GBTC)—from 1990 to 2023. Results: In 2023, Asia reported about 3.23 million new gastrointestinal cancer cases and 2.26 million deaths, resulting in 55.10 million DALYs. EC had the highest burden in East Asia [age-standardized incidence rate (ASIR): 11.41 per 100,000; age-standardized death rate (ASDR): 10.88 per 100,000; age-standardized DALY rate (ASDALYR): 244.41 per 100,000], while CRC, PC, and GBTC had a high burden in the high-income Asia-Pacific region. From 1990 to 2023, EC, LC, and GBTC declined in most regions, whereas PC increased except in East Asia. The burden was generally higher in males; however, in South Asia, females had higher ASIR (3.35 per 100,000), ASDR (3.44 per 100,000), and ASDALYR (82.94 per 100,000) for GBTC than males. Conclusions: The burden of gastrointestinal cancer in Asia is heavy and unevenly distributed; in the future, more targeted regional prevention and control strategies should be implemented based on socioeconomic levels and the characteristics of the disease spectrum.

## Simple Summary

Gastrointestinal cancers are a major health problem in Asia, but their burden differs across regions and population groups. This study examined six major gastrointestinal cancers in 33 Asian countries and territories from 1990 to 2023. In 2023, these cancers caused about 3.23 million new cases, 2.26 million deaths, and 55.10 million years of healthy life lost. The burden of esophageal, liver, and gallbladder and biliary tract cancers generally decreased over time, while pancreatic cancer increased in most regions. Males generally had a higher burden than females, although gallbladder and biliary tract cancer was more common among females in South Asia. These findings show the need for prevention and control strategies tailored to different regions and populations in Asia.

## 1. Introduction

GLOBOCAN 2024 data indicate that there were 20.6 million new cancer cases and 9.8 million cancer deaths worldwide [1]. Among these, gastrointestinal cancers [esophageal cancer (EC), stomach cancer (SC), colon and rectum cancer (CRC), liver cancer (LC), pancreatic cancer (PC), and gallbladder and biliary tract cancer (GBTC)] accounted for approximately one-quarter of all new cancer cases and one-third of all cancer-related deaths worldwide [2]. Among the top ten cancers by incidence and mortality globally in 2024, gastrointestinal cancers accounted for three and five types, respectively [1]. By 2050, the global burden of these six gastrointestinal cancers is projected to increase further due to population growth, accelerated aging, and continued exposure to risk factors [3].

Gastrointestinal cancers represent a major global public health challenge, with a particularly pronounced impact in Asia. Over the past three decades, cases of CRC, GBTC, PC, and LC have more than doubled [4]. By 2021, Asia had surpassed Europe, the Americas and Africa in terms of the absolute numbers of gastrointestinal cancer incidence, mortality and disability-adjusted life years (DALYs) [5]. Notably, over three-quarters of the world’s SC and EC patients reside in Asia [6].

Asia, being the most populous continent, bears a particularly heavy disease burden due to the high incidence and mortality rates of gastrointestinal cancers. Significant variations in population structure, socioeconomic levels, lifestyles, and healthcare resources among Asian countries may lead to heterogeneity in disease distribution and trends. However, existing research on the burden of gastrointestinal cancers in Asia remains relatively scarce [5,7]. Therefore, this study systematically analyzes trends in incidence, mortality, and DALYs for six major gastrointestinal cancers across Asia from 1990 to 2023, based on data from the Global Burden of Disease (GBD) 2023 study. It examines distribution disparities across sex, age groups, and countries/regions. The findings aim to update and supplement previous research, providing precise insights for regional disease prevention and control strategies.

## 2. Methods

### 2.1. Data Sources

This study utilized data from the GBD 2023 Global Health Data Exchange (GHDx) query tool (http://ghdx.healthdata.org/gbd-results-tool, accessed on 23 October 2025), which provides modeled estimates for 375 diseases and injuries across 204 countries and territories based on comprehensive modeling [8]. This study leveraged GBD 2023 data to quantitatively assess the disease burden of six major gastrointestinal cancers across five GBD subregions in Asia, focusing on disparities in incidence, mortality, and DALYs by sex and age group.

Based on the GBD 2023 geographic subregion classification, this study included a total of 33 countries and regions in Asia: 9 in Central Asia, 2 in East Asia, 4 in High-income Asia-Pacific, 5 in South Asia, and 13 in Southeast Asia. The detailed list of countries and regions in each subregion is provided in Table S1.

### 2.2. Estimating Methods

The GBD 2023 study employs its standardized stepwise statistical modeling framework—including models such as DisMod-MR 2.1 for disease incidence modeling and CODEm for cause-of-death modeling—to integrate data from multiple sources and generate estimates of indicators like incidence and mortality rates [9,10,11]. According to GBD 2023 definitions, incidence data for the six gastrointestinal cancers were mapped to the following ICD-10 codes: C15–C15.9 for EC; C16–C16.9 for SC; C18–C21.9 for CRC; C22–C22.8 for LC; C23–C24.9 for GBTC; and C25–C25.9 for PC. Mortality data were mapped to C15–C15.9, D00.1, and D13.0 for EC; C16–C16.9, D00.2, D13.1, and D37.1 for SC; C18–C21.9, D01.0–D01.3, D12–D12.9, and D37.3–D37.5 for CRC; C22–C22.8 and D13.4 for LC; C23–C24.9 and D13.5 for GBTC; and C25–C25.9 and D13.6–D13.7 for PC. Years lived with disability (YLDs) were estimated by multiplying the cause-age-sex-location-year-specific prevalence of sequelae (or the nature and duration of injuries) by the corresponding disability weights for each disease and injury, with adjustment for comorbidity [9]. Years of life lost (YLLs) were derived by multiplying the cause-age-sex-location-year-specific number of deaths by the standard life expectancy at the age of death [11]. DALYs represented the sum of YLDs and YLLs. The 95% uncertainty intervals (UIs) for all metrics were computed using the 2.5th and 97.5th percentiles from a 250-draw distribution for each metric [11].

### 2.3. Statistical Analysis

Age-standardized incidence rates (ASIRs), age-standardized death rates (ASDRs), and age-standardized disability-adjusted life year rates (ASDALYRs) were used to compare the burden of gastrointestinal cancers across different countries and regions. Estimated annual percentage changes (EAPCs) were used to assess trends in the burden of disease from 1990 to 2023.

*EAPC* is a widely used metric to quantify temporal trends in indicators such as incidence and prevalence over specific time periods. The *EAPC* is derived from a regression model that fits the natural logarithm of the age-standardized rate (ASR) to calendar year, with time serving as the independent variable [12]. The natural logarithm of each observed ASR is fitted to a straight line, and the *EAPC* is calculated based on the slope of this regression line [13]. We calculated *EAPC* using the following Formulas (1) and (2).


(1) y=α+βx+ε

(2) EAPC=100×(exp(β)−1)

In the regression model, *x* represents the calendar year, *y* denotes the natural logarithm of the rate (e.g., incidence or prevalence), *α* is the intercept, *β* is the slope, and *ε* represents the random error. The 95% confidence interval (CI) for the *EAPC* is also derived from this fitted model. Interpretation of the trend results is based on the 95% CI: a lower bound of the 95% CI greater than 0 indicates an upward trend, while an upper bound less than 0 indicates a downward trend. If the 95% CI includes 0, it suggests no statistically significant change in trend [14]. The sociodemographic index (SDI) is a composite measure of two demographic indicators (total fertility rate in people younger than 25 years and mean educational attainment for those aged 15 years and older) and an economic indicator (lag-distributed income per capita) [15]. SDI ranges from 0 to 1, with higher values indicating higher levels of socioeconomic development. In this study, LOESS smoothing was used to visualize the associations between SDI and disease burden indicators, and Spearman’s rank correlation analysis was performed to assess the correlations between SDI and age-standardized incidence, death, and DALY rates. The burden of gastrointestinal cancers among different subregions and countries was mainly ranked and compared based on point estimates. Statistical analyses and visualization of results were performed using R software (version 4.5.0).

## 3. Results

### 3.1. The Burden of Gastrointestinal Cancers in Asia in 2023

In 2023, approximately 3.23 million new gastrointestinal cancer cases were reported across the five Asian subregions. The highest number of new cases was accounted for by CRC (approximately 1.14 million), followed by SC, EC, and LC. As demonstrated in Table 1, the lowest incidences were observed in PC and GBTC. A total of about 2.26 million deaths were recorded, with SC being the leading cause of gastrointestinal cancer–related mortality (approximately 631,000 deaths), followed by CRC, EC, LC, PC, and GBTC (Table S2). Gastrointestinal cancers resulted in an estimated 55.10 million DALYs lost across the five subregions, with SC contributing the largest proportion (approximately 15.12 million), followed by CRC, EC, LC, PC, and GBTC (Table S3).

Compared with the other four regions, the ASIR [11.41 per 100,000 (95% UI: 9.83 to 12.99)], ASDR [10.88 per 100,000 (95% UI: 9.34 to 12.43)] and ASDALYR [244.41 per 100,000 (95% UI: 217.21 to 277.63)] of EC in East Asia were the highest, and the ASDR [16.54 per 100,000 (95% UI: 13.12 to 20.72)] and ASDALYR [388.47 per 100,000 (95% UI: 307.78 to 490.69)] of SC were the highest; the ASIR of SC and LC, and the ASIR, ASDR and ASDALYR of CRC, PC, GBTC were the highest in the high-income Asia-Pacific region (Table 1, Tables S2 and S3).

### 3.2. Temporal Trends of Gastrointestinal Cancers from 1990 to 2023

**Figure 1:**
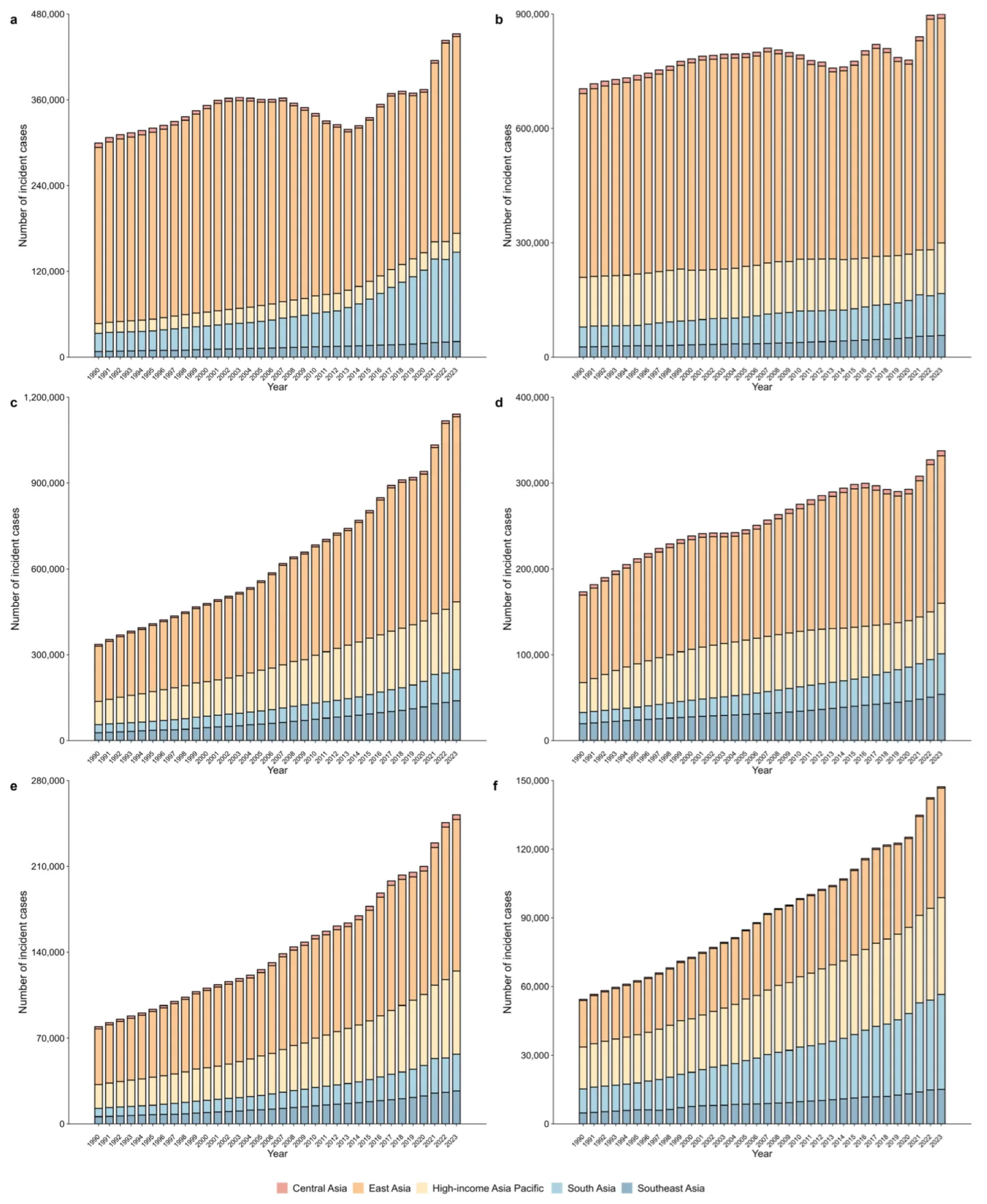
Number of incident cases for esophageal (**a**), stomach (**b**), colorectal (**c**), liver (**d**), pancreatic (**e**), and gallbladder and biliary tract (**f**) cancers in the five GBD subregions of Asia, 1990–2023.

**Figure 2:**
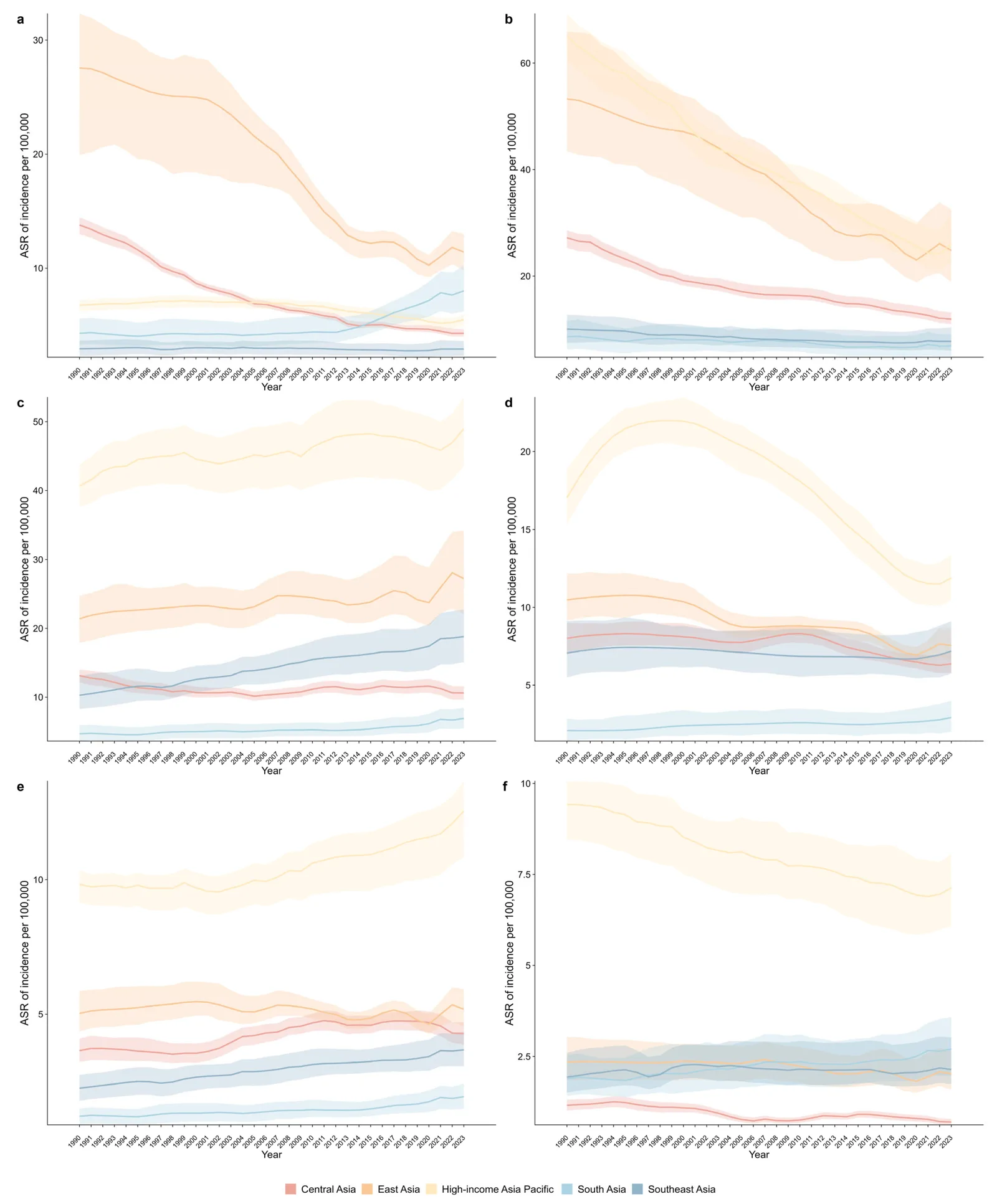
ASIRs for esophageal (**a**), stomach (**b**), colorectal (**c**), liver (**d**), pancreatic (**e**), and gallbladder and biliary tract (**f**) cancers in the five GBD subregions of Asia, 1990–2023. ASIRs: age-standardized incidence rates.

As demonstrated in Figure 1, Figure 2 and Figures S1–S4, from 1990 to 2023, the incidence, mortality, and DALYs for CRC, LC, PC, and GBTC generally increased across five Asian subregions, while DALYs for SC generally decreased. From 1990 to 2023, the ASIR, ASDR, and ASDALYR of EC decreased in four regions except South Asia. The ASIR and ASDR of EC decreased most significantly in Central Asia, with EAPC of −3.66 (95% CI: −3.86 to −3.47) and −3.67 (95% CI: −3.86 to −3.47), respectively. The ASIR, ASDR, and ASDALYR of SC showed a downward trend in the five regions, and the ASDR and ASDALYR of SC decreased most significantly in East Asia, with EAPC of −3.81 (95% CI: −4.07 to −3.54) and −4.12 (95% CI: −4.39 to −3.86), respectively. The ASDR and ASDALYR of CRC decreased most significantly in East Asia, while the ASIR, ASDR, and ASDALYR of CRC increased in South Asia and Southeast Asia. For LC, a downward trend was observed in all regions except South Asia, with the most pronounced reductions occurring in the high-income Asia-Pacific region. In contrast, the age-standardized rates of PC declined only in East Asia, while positive EAPC values in the other four regions indicated a continuous increase. The ASIR, ASDR and ASDALYR of PC increased most significantly in Southeast Asia, and the EAPC were 1.43 (95% CI: 1.37 to 1.50), 1.35 (95% CI: 1.28 to 1.41) and 1.44 (95% CI: 1.38 to 1.51), respectively. GBTC showed a consistent upward trend across South Asia (Table 1, Tables S2 and S3, and Figure S5).

### 3.3. The Burden of Gastrointestinal Cancers by Sex and Age Group

Figure S6 shows the number of incident cases, deaths, and DALYs of six gastrointestinal cancers in five subregions in 2023 by sex. Overall, the number of incident cases, deaths, and DALYs were higher in males than in females. In South Asia, females had higher ASIR, ASDR, and ASDALYR of GBTC than males, and the ASDALYR of CRC in females [145.27 per 100,000 (95% UI: 110.07 to 195.26)] was also higher than in males [142.54 per 100,000 (95% UI: 105.75 to 190.58)]; in addition, the ASIR, ASDR and ASDALYR of males in other regions were higher than those of females. From 1990 to 2023, the ASIR, ASDR, and ASDALYR of EC in females decreased most significantly in East Asia. The ASIR, ASDR, and ASDALYR of SC in females showed a clear decline in high-income Asia-Pacific and East Asia. The ASIR, ASDR, and ASDALYR of PC in females in high-income Asia-Pacific showed an upward trend, with EAPC of 1.01 (95% CI: 0.88 to 1.13), 0.71 (95% CI: 0.58 to 0.85), and 0.43 (95% CI: 0.32 to 0.55), respectively. Meanwhile, the ASIR, ASDR, and ASDALYR of PC in Central Asia showed an upward trend in both males and females. In South Asia, except for SC, the ASIR, ASDR, and ASDALYR of other gastrointestinal cancers all increased (Figure S5 and Tables S4–S6).

**Figure 3:**
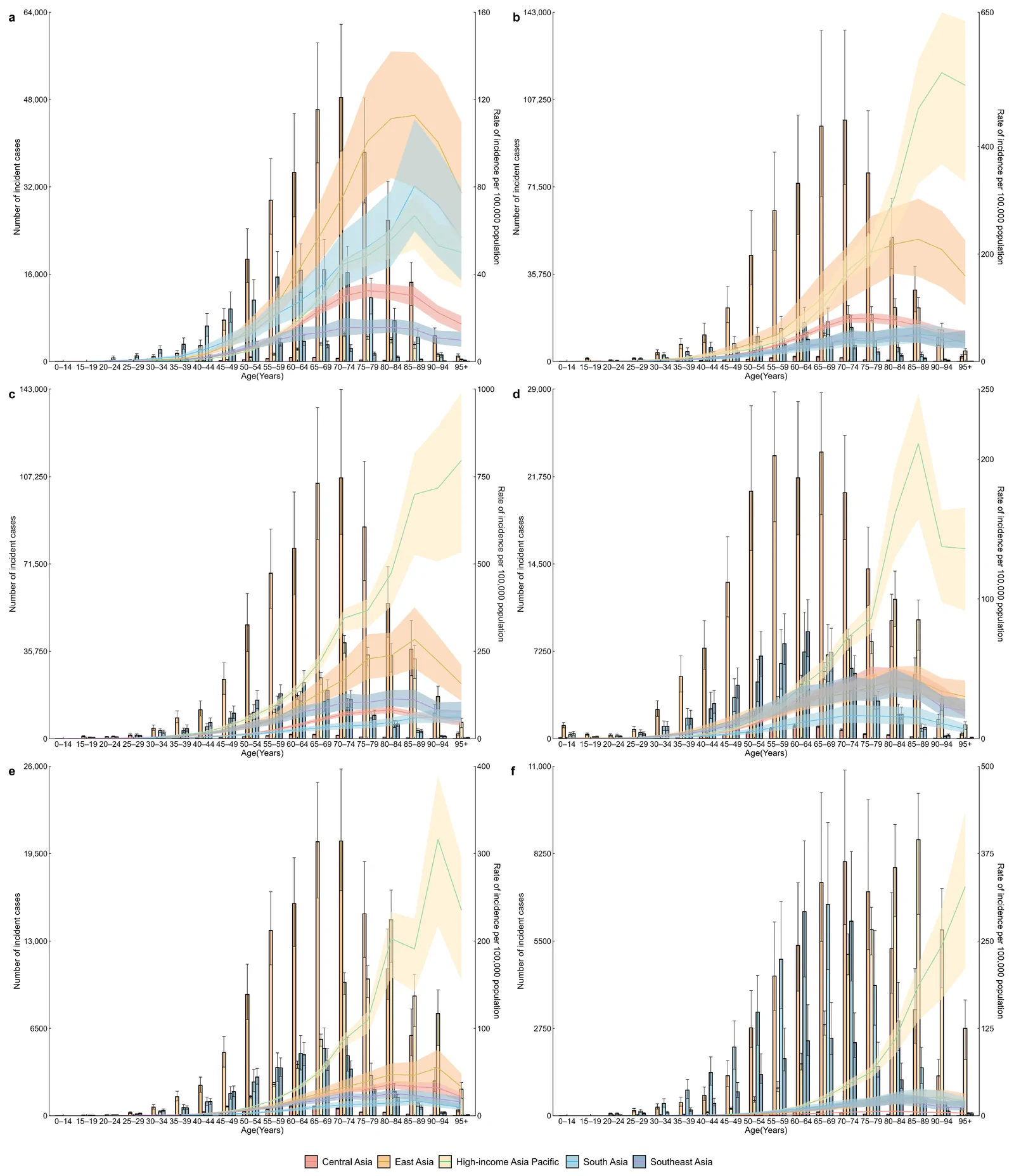
Number of incident cases and age-specific incidence rates for esophageal (**a**), stomach (**b**), colorectal (**c**), liver (**d**), pancreatic (**e**), and gallbladder and biliary tract (**f**) cancers by age group in the five GBD subregions of Asia, 2023.

Figure 3, Figures S7 and S8 show the incidence, mortality, and DALYs for different age groups. Overall, the burden is higher in middle-aged and elderly groups. Specifically, among people aged 65 and older in high-income Asia-Pacific countries, the incidence, mortality, and DALY rates for CRC, LC, PC, and GBTC were all higher than in the other four regions; in contrast, for the other four regions, the incidence, mortality, and DALY rates for gastrointestinal cancers generally increased with age, peaking at a certain age and then beginning to decline. For EC, the disease burden is particularly pronounced among people aged 50 and older in East Asia. Regarding SC, mortality and DALY rates in East Asia are higher than those in the high-income Asia-Pacific region for those under 85, whereas for those aged 85 and older, the high-income Asia-Pacific region has higher rates.

Further analysis by age and sex (Tables S7–S24) showed that in the 15–49, 50–69, and ≥70 age groups, incidence, mortality, and DALY rates for males were higher than those for females for all six gastrointestinal cancers in most regions. Only a few cancer types showed the opposite trend: in the 15–49 age group in high-income Asia-Pacific regions, mortality and DALY rates for SC were slightly higher among females than among males; in South Asia, among those aged 15–49, all three indicators for CRC were higher for females than for males; and for GBTC, incidence rates among those aged 15–49 in Central Asia, as well as incidence, mortality, and DALY rates across all three age groups in South Asia, were higher for females than for males.

### 3.4. The Burden of Gastrointestinal Cancers in Different Countries/Regions

**Figure 4:**
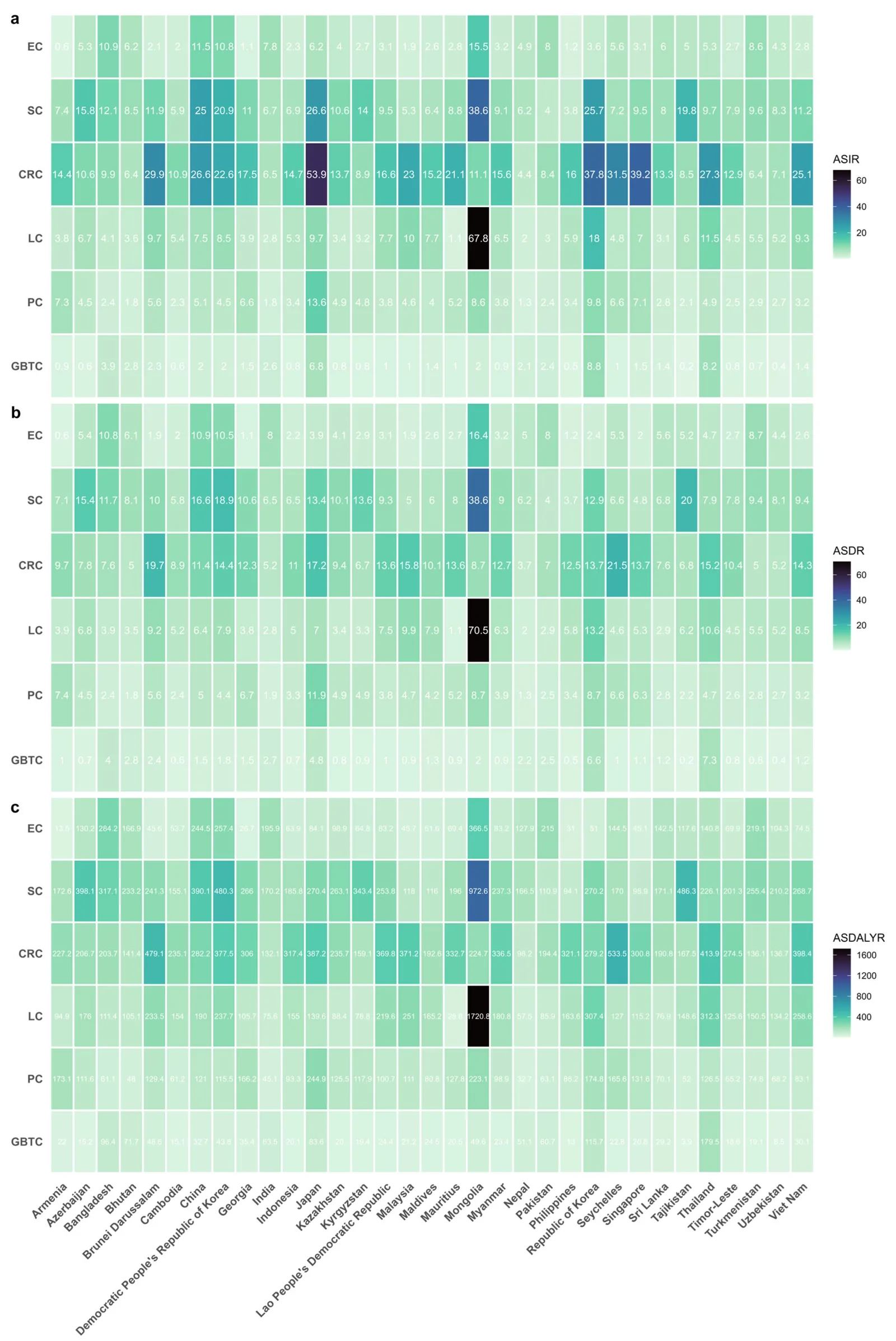
The age-standardized incidence (**a**), death (**b**), and DALY (**c**) rates for gastrointestinal cancers across Asian countries and regions, 2023. DALY: disability-adjusted life years.

Based on the point estimates, Mongolia had the highest ASIR, ASDR, and ASDALYR for EC, SC, and LC, whereas Japan had the highest point estimates for PC. For GBTC, Thailand had the highest point estimates for ASDR and ASDALYR, at 7.28 per 100,000 (95% UI: 5.78 to 9.74) and 179.52 per 100,000 (95% UI: 141.03 to 238.63), respectively (Figure 4). From 1990 to 2023, the ASIR, ASDR, and ASDALYR of EC and LC decreased most significantly in Kazakhstan, and the ASIR, ASDR, and ASDALYR of GBTC decreased most significantly in Turkmenistan; the ASIR for SC and the ASDR and ASDALYR for CRC showed the most significant decline in Singapore, with EAPC values of −3.36 (95% CI: −3.48 to −3.25), −2.34 (95% CI: −2.47 to −2.22), and −2.51 (95% CI: −2.62 to −2.40), respectively; and the ASIR, ASDR, and ASDALYR of PC increased in most countries (Figure S9, Tables S25–S30).

### 3.5. SDI and Gastrointestinal Cancer Burden

Figure S10 illustrates the long-term trends in the disease burden of six major gastrointestinal cancers across five GBD subregions in Asia from 1990 to 2023, as SDI changed. Overall, between 1990 and 2023, the SDI continued to rise across all subregions, with the disease burden of different cancer types exhibiting distinct patterns in response to changes in the SDI. For EC, the ASIR, ASDR, and ASDALYR generally followed an inverted V-shaped trend, gradually increasing until the SDI reached approximately 0.5, and then gradually decreasing. In contrast, the ASIR, ASDR, and ASDALYR for SC exhibited a wave-like trend, first increasing, then declining slightly, and subsequently rising again; the ASIR, ASDR, and ASDALYR for PC continued to increase as SDI rose, showing the most significant association with SDI (*p* < 0.001).

Figure S11 further illustrates the relationship between the ASIR, ASDR, and ASDALYR for six major gastrointestinal cancers in Asian countries in 2023 and the SDI. Overall, the patterns of association between different cancer types and the SDI vary. The ASIR, ASDR, and ASDALYR for CRC and PC generally increased as the SDI rose; the ASDR and ASDALYR for EC generally decreased as the SDI increased (*p* < 0.05). No significant correlation was observed between the SDI and SC, LC, or GBTC (*p* > 0.05). Furthermore, some countries deviated significantly from the overall trend. For example, the age-standardized rates for EC, SC, and LC in Mongolia, as well as for PC in Japan, were all higher than the levels expected based on their respective SDI values.

## 4. Discussion

This study updated and evaluated trends in the incidence, mortality, and disease burden of six major gastrointestinal cancers across five subregions in Asia from 1990 to 2023, and further analyzed differences in disease burden across regions with varying SDI levels. The results showed that gastrointestinal cancers in Asia caused approximately 3.23 million new cases, 2.26 million deaths, and 55.10 million DALYs in 2023. The spatial distribution showed obvious regional heterogeneity. Trend analysis showed that although the overall burden of gastrointestinal cancers in some regions showed a downward trend, PC only decreased in East Asia, and the EAPC in the remaining four regions showed an upward trend. While the overall burden of infection-related cancers, such as gastric cancer, has declined, the burden of CRC remains high, and the burden of PC is on the rise in most regions. These patterns highlight the importance of addressing both infection-related and metabolic and lifestyle-related risk factors through region-specific strategies to prevent and control gastrointestinal cancers in Asia.

The disease burden of EC was the highest in East Asia, significantly higher than the other four subregions. Although the overall EAPC showed a downward trend, indicating that the disease burden was gradually alleviated, EC remains a major public health concern in this region. Its high burden may be closely related to long-term lifestyle risk factors among the local population, including high-salt diets, smoking, alcohol consumption, and hot beverage intake [16,17]. Mongolia had the highest burden of EC among the countries analyzed, consistent with a recent comparative analysis of GBD 2021 and GLOBOCAN 2022, which also identified Mongolia as one of the countries most affected by EC in the Asia-Pacific region [18]. This high burden may be associated with regional differences in dietary patterns, environmental exposures, and infection-related risk factors [19,20]. From 1990 to 2023, the ASIR and ASDR in Central Asia decreased the most, while the downward trend among females in East Asia and Southeast Asia was also very obvious, which may be attributed to the promotion of tobacco control and alcohol restriction policies and the improvement of public health awareness [21,22]. Some countries in East Asia have made significant progress in the prevention and control of EC. For example, since the launch of the national key public health project in China in 2005, early cancer screening projects based on endoscopy have been carried out in more than 110 high-risk areas, providing systematic detection and follow-up management for individuals aged 40–69 at high risk, which has promoted the gradual alleviation of the burden of EC [23]. It is clear that continuous endoscopic screening, health education, and risk factor control in high-risk areas are key to reducing the burden of EC in East Asia and surrounding areas. Although the overall burden has decreased, significant sex differences still exist: ASIR and ASDR in males are generally higher than those in females, which is closely related to the higher smoking and drinking rates of males and the biological susceptibility caused by hormone differences. Studies have shown that androgen receptor activation may promote the early occurrence and progression of cancer, while estrogen receptor β has a certain protective effect in females [24,25]. It is worth noting that the burden of EC follows an inverted V-shaped pattern as the SDI increases: it rises gradually until the SDI reaches approximately 0.5 and then continues to decline. This suggests that the disease profile of EC in Asia exhibits characteristics of “poverty and economic transition”. The burden of EC in Mongolia is significantly higher than expected based on the SDI, which may be related to continued exposure to local risk factors such as alcohol consumption and a diet low in vegetables [26].

SC remains one of the most common gastrointestinal malignancies in Asia, with the highest ASDR and ASDALYR in East Asia. The highest ASIR was observed in the high-income Asia-Pacific region, highlighting regional differences in the distribution of SC burden. The high burden of SC in East Asia is primarily attributed to the continued high prevalence of *Helicobacter pylori* infection and traditional dietary patterns, and the risk of disease is further increased due to the prevalence of the more pathogenic East Asian *cagA* strain in the region [27,28]. In contrast, the high incidence in the high-income Asia-Pacific region may also be associated with lifestyle and demographic factors, including obesity, diabetes, high-fat diets, and population aging [29,30]. Mongolia has the highest ASIR, ASDR, and ASDALYR of SC in Asia. The high rate of *H. pylori* infection, a diet high in salt and red meat, and insufficient fruit and vegetable intake may contribute to the high burden of SC in Mongolia [19,20,31]. These combined risk factors have led to the high incidence of SC in the country for a long time. In some Asian countries, public health efforts, including screening and risk factor control, have been implemented to reduce the burden of SC. For example, China, Japan, and South Korea have significantly reduced the mortality rate of SC by promoting upper gastrointestinal endoscopy screening, improving dietary structure, and strengthening *H. pylori* prevention and control. Among these countries, Japan and South Korea established nationwide gastric cancer screening systems relatively early, while China began its screening efforts later, initially focusing on high-risk regions and populations, with coverage gradually expanding. These differences in screening models and implementation strategies may explain the variations in the extent of the decline in the burden of SC across the three countries [32]. Meanwhile, global and regional health initiatives such as the Bangkok Declaration on Cancer Control have helped optimize prevention and control strategies in the Asia-Pacific region by promoting risk stratification and equitable access to screening [22].

CRC is the most common gastrointestinal malignancy in the five subregions of Asia, with approximately 1.14 million new cases in 2023. In the high-income Asia-Pacific region, CRC had the highest ASIR, ASDR, and ASDALYR, with a substantially higher disease burden among individuals aged 65 years and older than in other subregions. On the one hand, this reflects the shift to a Westernized lifestyle caused by rapid economic development, such as increased high-fat diets, obesity, sedentary behavior, and the accumulation of metabolic risks such as diabetes [30]. On the other hand, it is also related to the increasing aging of the population, long-term exposure to carcinogenic factors, and the decline of immune function with age [33,34]. Together, these factors may contribute to the high burden of CRC in the region. In addition, rising CRC incidence among younger patients has been reported in some countries and regions [35,36], highlighting the importance of monitoring age-specific trends. In contrast, the ASDR and ASDALYR of CRC in East Asia decreased most significantly, suggesting that the prevention, control, and screening systems in some countries have achieved remarkable results. Among them, China promoted fecal immunochemical testing and colonoscopy screening in high-risk groups, which improved the early diagnosis rate and reduced the mortality rate [37]. Singapore’s Screen for Life program offers subsidized screening based on annual fecal immunochemical testing, and individuals with positive fecal immunochemical testing results can receive subsidized colonoscopy at designated centers, thereby significantly increasing screening participation rates and improving outcomes [38]. These successful practices provide valuable insights for other low- and middle-income countries in the region, but their widespread adoption remains limited by endoscopy resources and the coverage capacity of primary healthcare. In terms of sex differences, the overall CRC burden in males is higher than that in females, which may be related to the higher proportion of smoking and drinking in males, as well as hormonal differences. Studies have shown that low methylation of the androgen receptor gene increases the risk of CRC in males, while estrogen receptor β plays a protective role in females and inhibits tumor growth [24,25]. These findings highlight the importance of considering sex and regional differences when designing CRC prevention and control strategies, with interventions tailored to different populations. From the perspective of SDI, the burden of CRC increases as the SDI rises, which is highly consistent with the higher prevalence of metabolic risks in high-income regions.

In 2023, the ASIR, ASDR, and ASDALYR of LC showed a downward trend in all four Asian subregions except South Asia, indicating a gradual reduction in the overall disease burden, although marked regional disparities remained. Hepatitis B virus (HBV) and hepatitis C virus (HCV) remain the main risk factors for LC [39], and their epidemic patterns determine the differences in disease burden in different regions. In 2012, East and Southeast Asia accounted for the majority of liver cancer cases worldwide. This high burden may be related to the high prevalence of HBV infection, as these regions harbor approximately three-quarters of the global population of chronic HBV carriers [40]. Mongolia had the highest ASIR, ASDR, and ASDALYR of LC in Asia, which was closely related to the high prevalence of HBV and HCV, alcohol consumption, obesity, increased risk of metabolic liver disease, and lifestyle factors such as high-fat diet and lack of exercise [41,42]. In contrast, Kazakhstan had the largest decrease in LC burden, suggesting that its public health interventions in the prevention and control of viral hepatitis have achieved significant results. In East Asia, despite the persistently high burden, the ASDR and ASDALYR of LC declined over time. This trend may be partly associated with the expansion of antiviral treatment, increased hepatitis B vaccination coverage, and improvements in chronic liver disease management [43]. Taking China as an example, the national immunization program has promoted widespread vaccination with the hepatitis B vaccine and strengthened virus screening, antiviral treatment, and regular monitoring in high-risk groups, which has significantly reduced the burden of LC [44]. Studies have shown that China has made substantial progress in HBV prevention and control, with HBV endemicity shifting from high to intermediate levels and liver cancer incidence and mortality decreasing by 10.04% and 22.35%, respectively [45]. Overall, comprehensive prevention and control measures for viral hepatitis, alcohol consumption, and metabolic diseases are driving the continuous decline of LC burden in Asia.

The ASIR, ASDR, and ASDALYR of PC only slightly decreased in East Asia, while the EAPC of the other four subregions were all positive, indicating that the overall trend was still upward. The ASIR and ASDR in the high-income Asia-Pacific region were among the highest across the Asian subregions. Japan had the highest ASIR, ASDR, and ASDALYR in Asia. These patterns may be associated with population aging, the prevalence of diabetes and smoking, and dietary and other metabolic risk factors [46,47]. Among them, smoking and diabetes have become important drivers of PC incidence in Asia. Previous studies have shown that smoking can increase the risk of PC by 70–80% [48,49,50], and about 30% of smokers worldwide currently live in Asia [51]. At the same time, diabetes can increase the risk of PC by about 1.4 times [52]. The accumulation of these behavioral and metabolic risk factors has jointly exacerbated the disease burden of PC in Asia. Unlike EC or CRC, PC lacks effective screening methods. The disease often presents with few noticeable symptoms in its early stages, and most cases are diagnosed at intermediate or advanced stages, contributing to poor prognosis and high mortality [53]. The sensitivity of existing serological markers is limited, and the cost of imaging examination is high, making it difficult to achieve universal screening [54]. Some countries have explored the precise monitoring model based on high-risk groups (such as family history, chronic pancreatitis, or diabetic patients), but the overall prevention and control system is still imperfect [55]. PC is the gastrointestinal cancer most strongly associated with the SDI among the six types; its burden increases steadily as the SDI rises, and the EAPC is positive in all four regions except East Asia, suggesting that as economic levels across Asia continue to rise, this cancer will remain one of the most serious challenges in the future.

In 2023, although GBTC incidence was relatively low compared with that of the other five gastrointestinal cancers in Asia, substantial regional differences in disease burden persisted. These differences may be associated with variations in risk factor exposure and socioeconomic conditions. In South Asia, the burden of GBTC is higher in females than in males, which may be associated with increasing obesity rates and the effects of estrogen on bile metabolism and gallstone formation [56,57]. Gallstones are the main risk factor, and metabolic abnormalities and chronic cholecystitis may also contribute to carcinogenesis [58]. In addition, the delay in diagnosis and insufficient medical accessibility caused by socioeconomic inequality further aggravated the disease burden of females [59]. In some countries, environmental and infectious factors also play an important role. For example, the high incidence of GBTC in Thailand may be associated with exposure to parasites, such as liver flukes, and other region-specific risk factors [60]. Despite Thailand’s relatively high overall level of economic development, disparities in access to healthcare between urban and rural areas may pose challenges for timely diagnosis. These differences in epidemic trends reflect the significant differences in the implementation of prevention and control measures and the accessibility of medical resources in different regions.

This study is based on GBD 2023 and has the following limitations. First, although GBD 2023 improves the comparability of estimates through standardized statistical modeling and the integration of data from multiple sources, its disease burden estimates still depend on model assumptions and the quality of the underlying data. For countries and regions with inadequate cancer registration and mortality reporting systems, the estimates rely more heavily on model extrapolation, which may introduce some uncertainty. Therefore, country and subregion rankings based on point estimates should be interpreted cautiously, taking into account the uncertainty reflected by the 95% UIs. Second, as this study is based on aggregated data at the national level, it fails to reflect the heterogeneity of the disease burden within countries (e.g., across different provinces or between urban and rural areas) and cannot perform stratified analyses for specific cancer subtypes (e.g., esophageal squamous cell carcinoma versus adenocarcinoma), even though these subtypes differ significantly in epidemiological characteristics, risk factor profiles, and prevention and control strategies. Future iterations of the GBD study that disaggregate results by histological subtype would allow more precise risk attribution and more targeted public health interventions. Third, this study primarily analyzed the spatiotemporal trends in disease burden and did not further examine the levels of exposure to risk factors in each region or their contribution to the burden of gastrointestinal cancers. Future studies integrating risk factor exposure data and disease burden estimates may help clarify these relationships. Fourth, this study included data from 33 countries and regions in Asia; although it covered major population and disease burden areas, it did not fully encompass all Asian countries, which may have affected the comprehensiveness of the results and regional comparisons to some extent.

## 5. Conclusions

Overall, although the age-standardized burden of gastrointestinal cancers has generally declined across most regions of Asia over the past 30-plus years, the absolute burden of disease remains high due to factors such as population growth, population aging, and continued exposure to risk factors. Currently, gastrointestinal cancers remain a major public health challenge in Asia, and the disease spectrum is gradually shifting from infection-driven to metabolism- and lifestyle-driven. This study reveals significant differences in the disease burden across different cancer types, regions, populations, and levels of sociodemographic development, providing a crucial basis for identifying priority populations for prevention and control and optimizing resource allocation. In the future, Asian countries should optimize gastrointestinal cancer prevention and control strategies according to varying levels of socioeconomic development and disease spectrum characteristics, and promote the rational allocation of health resources to address the evolving burden of gastrointestinal cancer.

## Figures and Tables

**Table 1:** Incidence, age-standardized rates, and changes in gastrointestinal cancers in the five GBD subregions of Asia, 1990–2023.

	1990 Incidence (95% UI)	2023 Incidence (95% UI)	1990 ASIR per 100,000 (95% UI)	2023 ASIR per 100,000 (95% UI)	1990 to 2023 EAPC (95% CI)
Central Asia					
EC	6278 (5922 to 6573)	3836 (3589 to 4132)	13.79 (13.00 to 14.44)	4.29 (4.02 to 4.64)	−3.66 (−3.86 to −3.47)
SC	12,783 (11,876 to 13,427)	10,866 (10,043 to 12,047)	27.19 (25.29 to 28.59)	11.91 (10.99 to 13.19)	−2.30 (−2.43 to −2.16)
CRC	6179 (5701 to 6623)	9509 (8652 to 10,386)	13.13 (12.13 to 14.03)	10.60 (9.65 to 11.56)	−0.19 (−0.40 to 0.02)
LC	3818 (3324 to 4291)	5792 (5273 to 6444)	8.01 (6.98 to 9.00)	6.37 (5.79 to 7.12)	−0.75 (−0.94 to −0.56)
PC	1685 (1492 to 1887)	3808 (3453 to 4166)	3.65 (3.22 to 4.09)	4.28 (3.88 to 4.70)	0.99 (0.77 to 1.21)
GBTC	518 (456 to 587)	609 (543 to 687)	1.16 (1.01 to 1.31)	0.70 (0.62 to 0.79)	−1.57 (−1.95 to −1.19)
East Asia					
EC	246,319 (175,140 to 289,116)	275,284 (239,158 to 314,352)	27.56 (19.91 to 32.31)	11.41 (9.83 to 12.99)	−3.35 (−3.67 to −3.02)
SC	482,062 (390,157 to 598,476)	589,094 (448,295 to 771,331)	53.27 (43.44 to 65.92)	24.80 (18.87 to 32.49)	−2.73 (−2.94 to −2.53)
CRC	193,373 (161,177 to 223,997)	646,371 (523,490 to 810,250)	21.40 (17.89 to 24.73)	27.24 (22.14 to 34.24)	0.49 (0.38 to 0.61)
LC	101,992 (89,212 to 118,352)	171,648 (149,780 to 196,294)	10.48 (9.16 to 12.18)	7.55 (6.63 to 8.65)	−1.30 (−1.46 to −1.15)
PC	45,482 (39,419 to 53,023)	123,714 (108,852 to 140,968)	5.03 (4.36 to 5.86)	5.19 (4.54 to 5.93)	−0.20 (−0.32 to −0.07)
GBTC	20,232 (15,950 to 26,514)	47,872 (37,644 to 63,172)	2.34 (1.85 to 3.03)	2.02 (1.59 to 2.67)	−0.65 (−0.80 to −0.50)
High-income Asia-Pacific					
EC	13,699 (12,652 to 14,617)	26,591 (23,714 to 28,840)	6.76 (6.24 to 7.22)	5.49 (4.95 to 5.94)	−0.86 (−1.06 to −0.67)
SC	130,432 (121,345 to 138,727)	132,899 (111,235 to 157,285)	65.07 (60.53 to 69.28)	26.05 (22.67 to 31.62)	−3.01 (−3.10 to −2.91)
CRC	81,219 (75,474 to 87,077)	236,751 (204,745 to 260,800)	40.66 (37.68 to 43.76)	48.99 (43.49 to 53.55)	0.38 (0.30 to 0.46)
LC	34,813 (31,357 to 38,672)	58,824 (50,581 to 66,132)	17.03 (15.32 to 18.91)	11.90 (10.47 to 13.40)	−1.87 (−2.30 to −1.43)
PC	19,460 (18,169 to 20,439)	67,615 (56,094 to 75,114)	9.83 (9.14 to 10.35)	12.56 (10.84 to 13.67)	0.71 (0.60 to 0.82)
GBTC	18,351 (16,578 to 19,552)	42,335 (34,441 to 48,274)	9.42 (8.46 to 10.06)	7.13 (6.09 to 8.06)	−0.99 (−1.04 to −0.95)
South Asia					
EC	25,512 (19,497 to 33,089)	124,870 (96,957 to 159,930)	4.29 (3.30 to 5.58)	8.01 (6.22 to 10.28)	1.78 (1.32 to 2.25)
SC	52,176 (37,755 to 70,896)	109,691 (79,402 to 145,048)	8.59 (6.22 to 11.66)	6.89 (4.99 to 9.10)	−0.78 (−0.91 to −0.65)
CRC	28,250 (21,865 to 35,479)	109,320 (85,814 to 134,548)	4.68 (3.63 to 5.92)	6.91 (5.46 to 8.50)	0.99 (0.82 to 1.16)
LC	13,048 (8817 to 17,793)	47,365 (32,749 to 64,849)	2.08 (1.41 to 2.85)	2.92 (2.02 to 4.00)	0.83 (0.70 to 0.97)
PC	6849 (5099 to 8573)	30,015 (22,878 to 37,389)	1.20 (0.89 to 1.50)	1.93 (1.48 to 2.42)	1.27 (1.10 to 1.44)
GBTC	10,473 (7899 to 14,001)	41,412 (30,239 to 54,811)	1.88 (1.42 to 2.54)	2.71 (1.97 to 3.58)	1.05 (0.94 to 1.16)
Southeast Asia					
EC	7795 (6129 to 9726)	21,797 (17,420 to 27,138)	2.93 (2.31 to 3.66)	2.91 (2.33 to 3.61)	−0.18 (−0.26 to −0.10)
SC	26,808 (19,867 to 33,728)	57,095 (44,051 to 76,336)	10.03 (7.43 to 12.72)	7.74 (6.00 to 10.37)	−0.92 (−1.03 to −0.82)
CRC	27,377 (21,972 to 34,978)	139,000 (111,194 to 168,650)	10.27 (8.29 to 13.17)	18.80 (15.09 to 22.76)	1.81 (1.74 to 1.88)
LC	19,640 (15,150 to 25,457)	53,837 (43,050 to 68,491)	7.06 (5.49 to 9.12)	7.18 (5.74 to 9.11)	−0.29 (−0.37 to −0.21)
PC	5841 (4638 to 7087)	26,897 (22,472 to 31,854)	2.25 (1.78 to 2.75)	3.67 (3.07 to 4.35)	1.43 (1.37 to 1.50)
GBTC	4794 (3747 to 6319)	15,086 (12,339 to 21,383)	1.93 (1.50 to 2.60)	2.14 (1.74 to 3.02)	0.09 (−0.06 to 0.23)

EC: esophageal cancer; SC: stomach cancer; CRC: colon and rectum cancer; LC: liver cancer; PC: pancreatic cancer; GBTC: gallbladder and biliary tract cancer.

## Data Availability

The data used for the analyses are publicly available from the Global Burden of Disease Results Tool of the Institute for Health Metrics and Evaluation (https://vizhub.healthdata.org/gbd-results/; accessed on 23 October 2025).

## Supplementary Materials

The following supporting information can be downloaded at https://www.mdpi.com/article/10.3390/cancers18213535/s1. Figure S1: Number of deaths for esophageal (a), stomach (b), colorectal (c), liver (d), pancreatic (e), and gallbladder and biliary tract (f) cancers in five GBD subregions of Asia, 1990–2023. Figure S2: ASDRs for esophageal (a), stomach (b), colorectal (c), liver (d), pancreatic (e), and gallbladder and biliary tract (f) cancers in five GBD subregions of Asia, 1990–2023. ASDRs: age-standardized death rates. Figure S3: Number of DALYs for esophageal (a), stomach (b), colorectal (c), liver (d), pancreatic (e), and gallbladder and biliary tract (f) cancers in five GBD subregions of Asia, 1990–2023. DALYs: disability-adjusted life years. Figure S4: ASDALYR for esophageal (a), stomach (b), colorectal (c), liver (d), pancreatic (e), and gallbladder and biliary tract (f) cancers in five GBD subregions of Asia, 1990–2023. ASDALYR: age-standardized disability-adjusted life years rate. Figure S5: The EAPCs in the age-standardized incidence (a), death (b), and DALY (c) rates for gastrointestinal cancers across five GBD subregions of Asia, 1990–2023. EAPCs: estimated annual percent changes; DALY: disability-adjusted life years. Figure S6: The number of incident cases (a), deaths (b), and DALYs (c) for gastrointestinal cancers by sex in five GBD subregions of Asia, 2023. DALYs: disability-adjusted life years. Figure S7: Deaths and age-specific death rates for esophageal (a), stomach (b), colorectal (c), liver (d), pancreatic (e), and gallbladder and biliary tract (f) cancers by age group in five GBD subregions of Asia, 2023. Figure S8: DALYs and age-specific DALY rates for esophageal (a), stomach (b), colorectal (c), liver (d), pancreatic (e), and gallbladder and biliary tract (f) cancers by age group in five GBD subregions of Asia, 2023. DALYs: disability-adjusted life years. Figure S9: The EAPCs in the age-standardized incidence (a), death (b), and DALY (c) rates for gastrointestinal cancers across Asian countries and regions, 1990–2023. EAPCs: estimated annual percent changes; DALY: disability-adjusted life years. Figure S10: Temporal trends in age-standardized incidence (a), death (b), and DALY (c) rates of gastrointestinal cancers by SDI in five GBD subregions of Asia, 1990–2023. DALY: disability-adjusted life year; SDI: Socio-demographic Index. Figure S11: Associations between SDI and age-standardized incidence (a), death (b), and DALY (c) rates of gastrointestinal cancers across Asian countries and regions, 2023. SDI: Socio-demographic Index; DALY: disability-adjusted life year. Table S1: Geographic subregions and countries/regions included in the study. Table S2: Deaths, age-standardized rates, and changes of gastrointestinal cancers in five GBD subregions of Asia, 1990–2023. Table S3: DALYs, age-standardized rates, and changes of gastrointestinal cancers in five GBD subregions of Asia, 1990–2023. Table S4: Incidence, age-standardized rates, and changes of gastrointestinal cancers by sex in five GBD subregions of Asia, 1990–2023. Table S5: Deaths, age-standardized rates, and changes of gastrointestinal cancers by sex in five GBD subregions of Asia, 1990–2023. Table S6: DALYs, age-standardized rates, and changes of gastrointestinal cancers by sex in five GBD subregions of Asia, 1990–2023. Table S7: Age-specific incidence rates and male-to-female ratios of esophageal cancer by region in Asia, 2023. Table S8: Age-specific death rates and male-to-female ratios of esophageal cancer by region in Asia, 2023. Table S9: Age-specific DALY rates and male-to-female ratios of esophageal cancer by region in Asia, 2023. Table S10: Age-specific incidence rates and male-to-female ratios of stomach cancer by region in Asia, 2023. Table S11: Age-specific death rates and male-to-female ratios of stomach cancer by region in Asia, 2023. Table S12: Age-specific DALY rates and male-to-female ratios of stomach cancer by region in Asia, 2023. Table S13: Age-specific incidence rates and male-to-female ratios of colorectal cancer by region in Asia, 2023. Table S14: Age-specific death rates and male-to-female ratios of colorectal cancer by region in Asia, 2023. Table S15: Age-specific DALY rates and male-to-female ratios of colorectal cancer by region in Asia, 2023. Table S16: Age-specific incidence rates and male-to-female ratios of liver cancer by region in Asia, 2023. Table S17: Age-specific death rates and male-to-female ratios of liver cancer by region in Asia, 2023. Table S18: Age-specific DALY rates and male-to-female ratios of liver cancer by region in Asia, 2023. Table S19: Age-specific incidence rates and male-to-female ratios of pancreatic cancer by region in Asia, 2023. Table S20: Age-specific death rates and male-to-female ratios of pancreatic cancer by region in Asia, 2023. Table S21: Age-specific DALY rates and male-to-female ratios of pancreatic cancer by region in Asia, 2023. Table S22: Age-specific incidence rates and male-to-female ratios of gallbladder and biliary tract cancer by region in Asia, 2023. Table S23: Age-specific death rates and male-to-female ratios of gallbladder and biliary tract cancer by region in Asia, 2023. Table S24: Age-specific DALY rates and male-to-female ratios of gallbladder and biliary tract cancer by region in Asia, 2023. Table S25: Incidence, death, DALYs, age-standardized rates, and changes from 1990 to 2023 for esophageal cancer in Asian countries and regions. Table S26: Incidence, death, DALYs, age-standardized rates, and changes from 1990 to 2023 for stomach cancer in Asian countries and regions. Table S27: Incidence, death, DALYs, age-standardized rates, and changes from 1990 to 2023 for colorectal cancer in Asian countries and regions. Table S28: Incidence, death, DALYs, age-standardized rates, and changes from 1990 to 2023 for liver cancer in Asian countries and regions. Table S29: Incidence, death, DALYs, age-standardized rates, and changes from 1990 to 2023 for pancreatic cancer in Asian countries and regions. Table S30: Incidence, death, DALYs, age-standardized rates, and changes from 1990 to 2023 for gallbladder and biliary tract cancer in Asian countries and regions.

## Abbreviations

The following abbreviations are used in this manuscript:

ASDALYR	Age-standardized disability-adjusted life year rate
ASDR	Age-standardized death rate
ASIR	Age-standardized incidence rate
ASR	Age-standardized rate
CI	Confidence interval
CRC	Colon and rectum cancer
DALY	Disability-adjusted life year
EAPC	Estimated annual percentage change
EC	Esophageal cancer
GBD	Global Burden of Disease
GBTC	Gallbladder and biliary tract cancer
GHDx	Global Health Data Exchange
HBV	Hepatitis B virus
HCV	Hepatitis C virus
LC	Liver cancer
PC	Pancreatic cancer
SC	Stomach cancer
SDI	Sociodemographic index
UI	Uncertainty interval
YLD	Years lived with disability
YLL	Years of life lost
