# Supplementary material for: Trends and Patterns in Gastrointestinal Cancer Burden in Asia, 1990–2023: Evidence from the Global Burden of Disease Study 2023
Source: Cancers (Basel). 2026 Nov 3;18(21):3535. doi: 10.3390/cancers18213535 (PMC13761716; doi:10.3390/cancers18213535)
Supplement: Supplementary file 1 [file cancers-18-03535-s001.zip › cancers-4621600-supplementary.pdf]

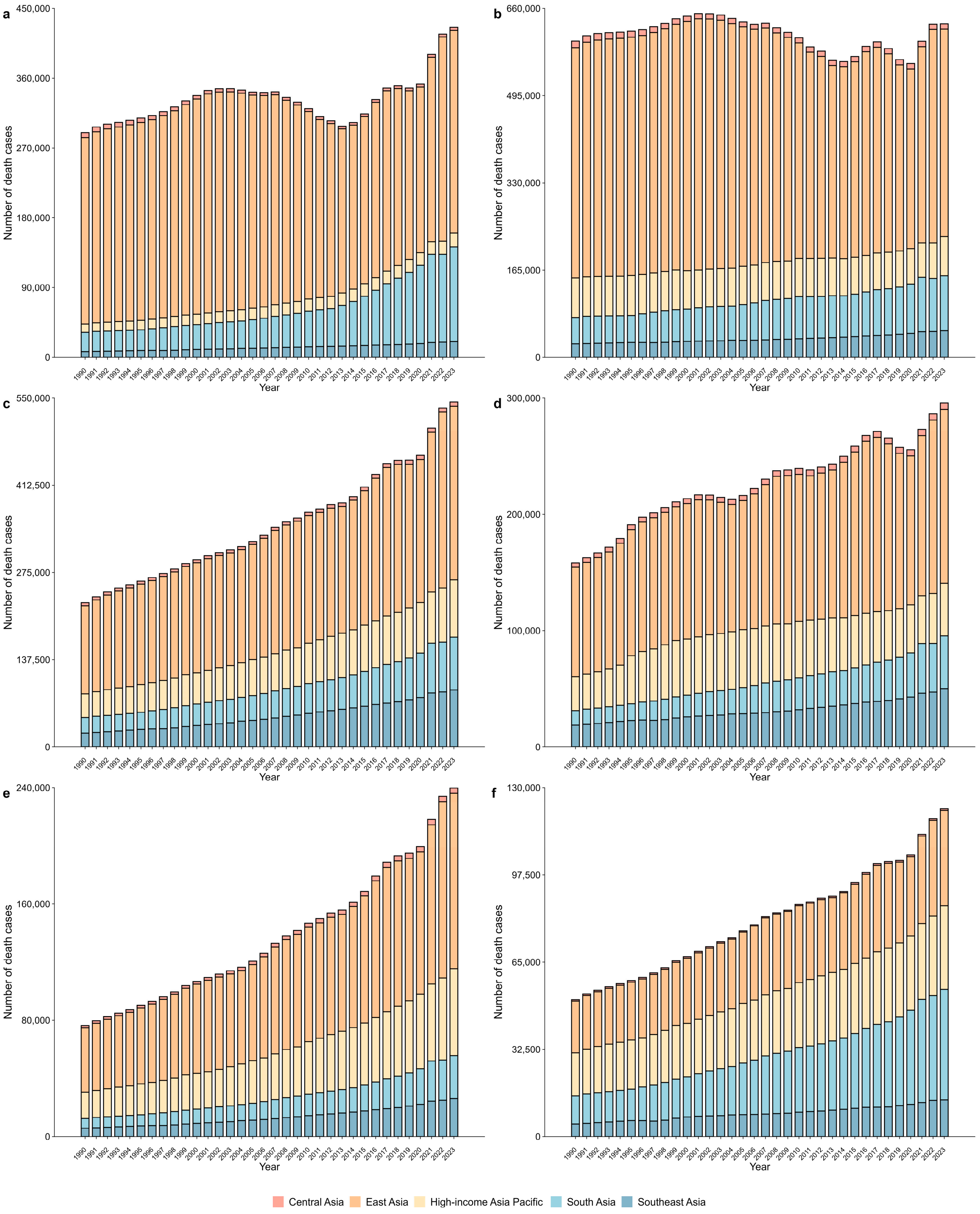

Figure S1. Number of deaths for esophageal (a), stomach (b), colorectal (c), liver (d), pancreatic (e), and gallbladder and biliary tract (f) cancers in five GBD subregions of Asia, 1990–2023.

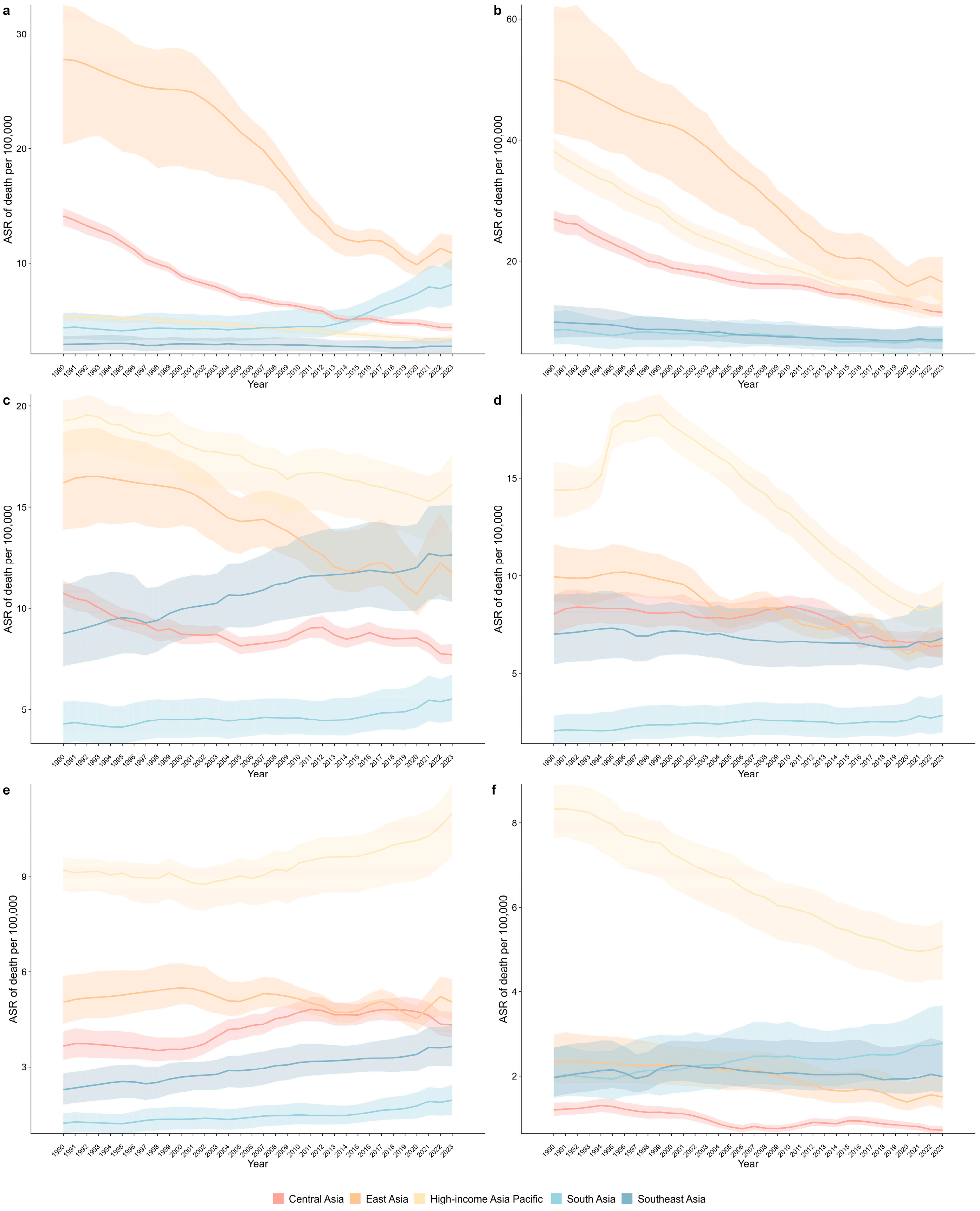

Figure S2. ASDRs for esophageal (a), stomach (b), colorectal (c), liver (d), pancreatic (e), and gallbladder and biliary tract (f) cancers in five GBD subregions of Asia, 1990–2023. ASDRs: age-standardized death rates.

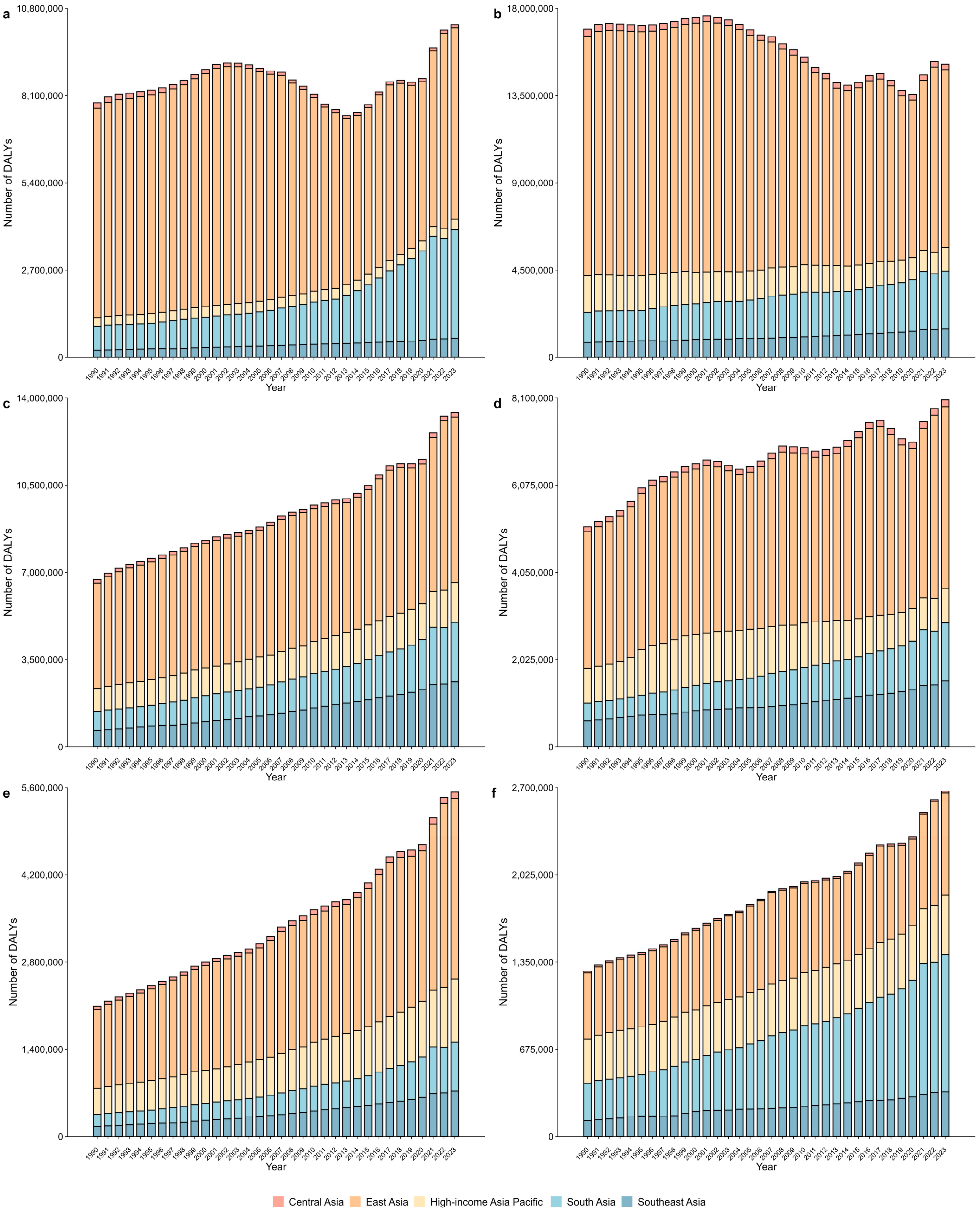

Figure S3. Number of DALYs for esophageal (a), stomach (b), colorectal (c), liver (d), pancreatic (e), and gallbladder and biliary tract (f) cancers in five GBD subregions of Asia, 1990–2023. DALYs: disability-adjusted life years.

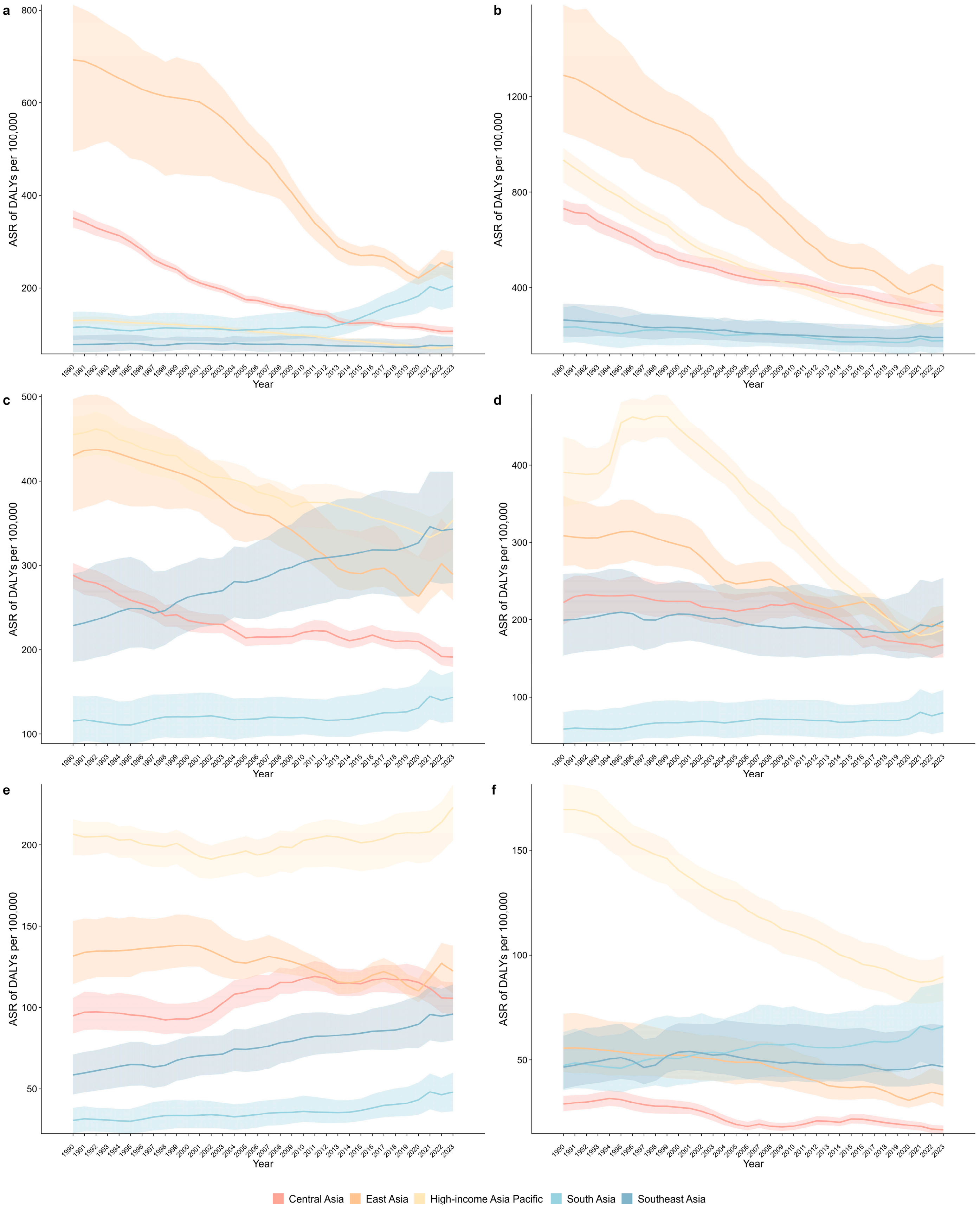

Figure S4. ASDALYR for esophageal (a), stomach (b), colorectal (c), liver (d), pancreatic (e), and gallbladder and biliary tract (f) cancers in five GBD subregions of Asia, 1990–2023. ASDALYR: age-standardized disability-adjusted life years rate.

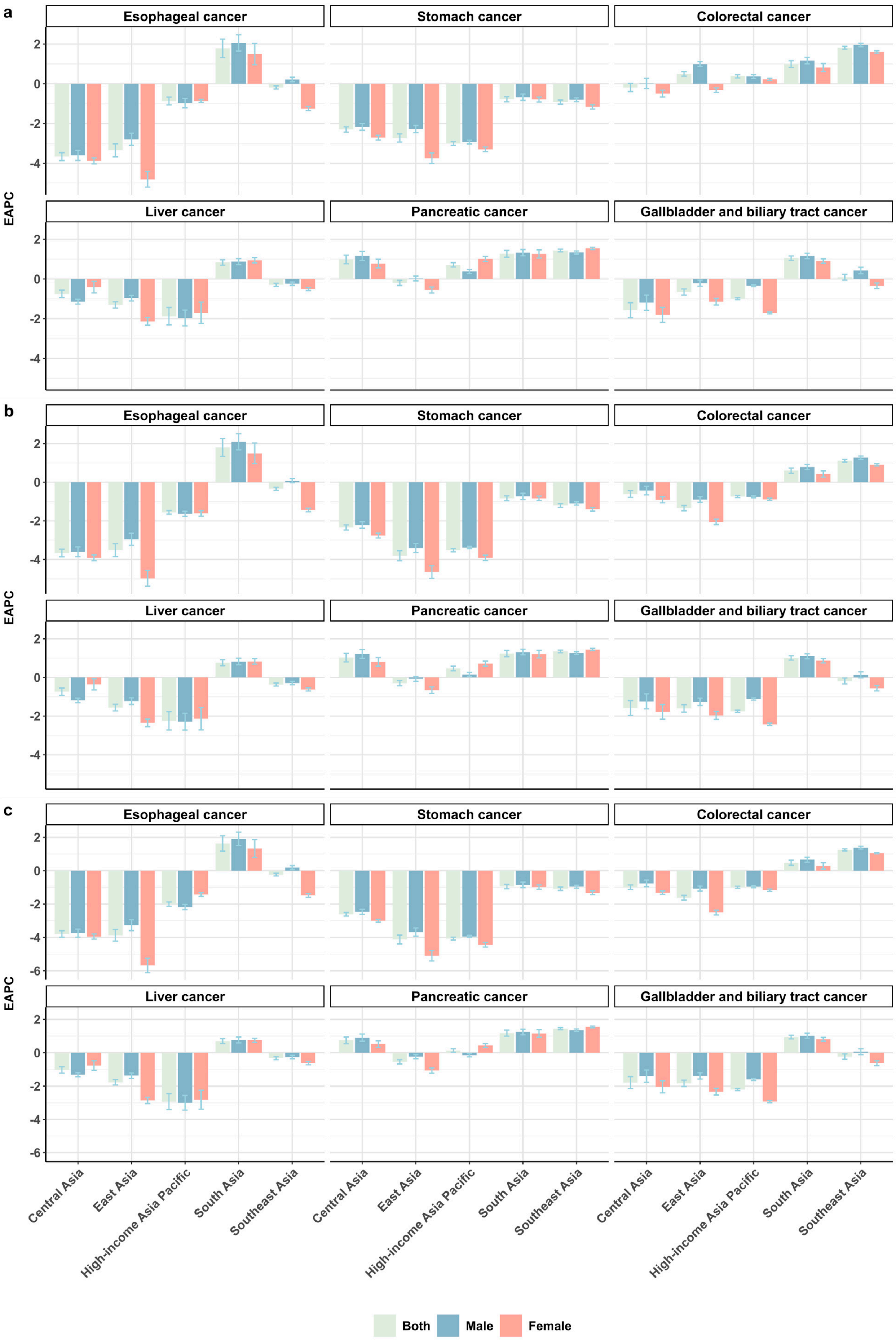

Figure S5. The EAPCs in the age-standardized incidence (a), death (b), and DALY (c) rates for gastrointestinal cancers across five GBD subregions of Asia, 1990–2023. EAPCs: estimated annual percent changes; DALY: disability-adjusted life years.

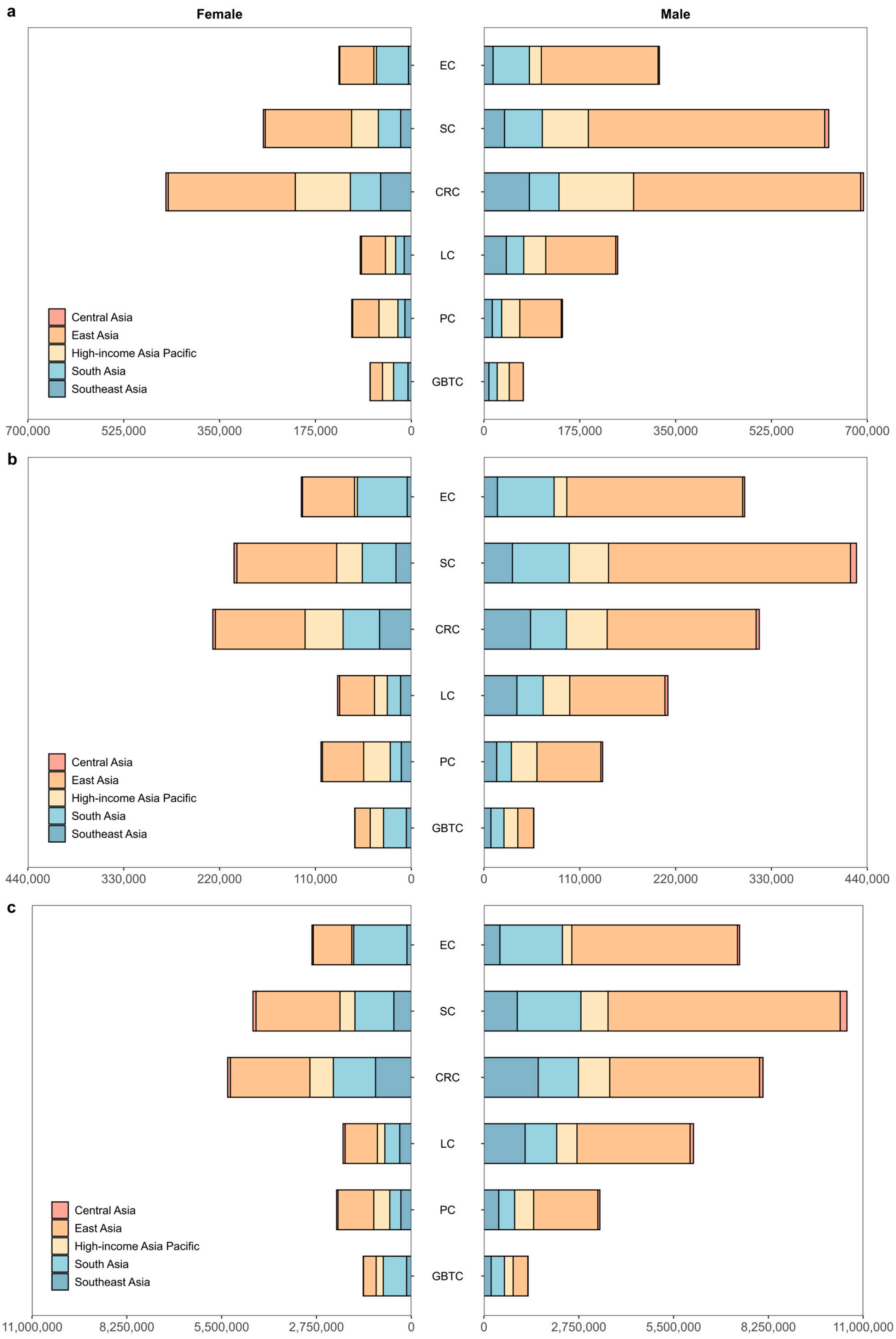

Figure S6. The number of incident cases (a), deaths (b), and DALYs (c) for gastrointestinal cancers by sex in five GBD subregions of Asia, 2023. DALYs: disability-adjusted life years.

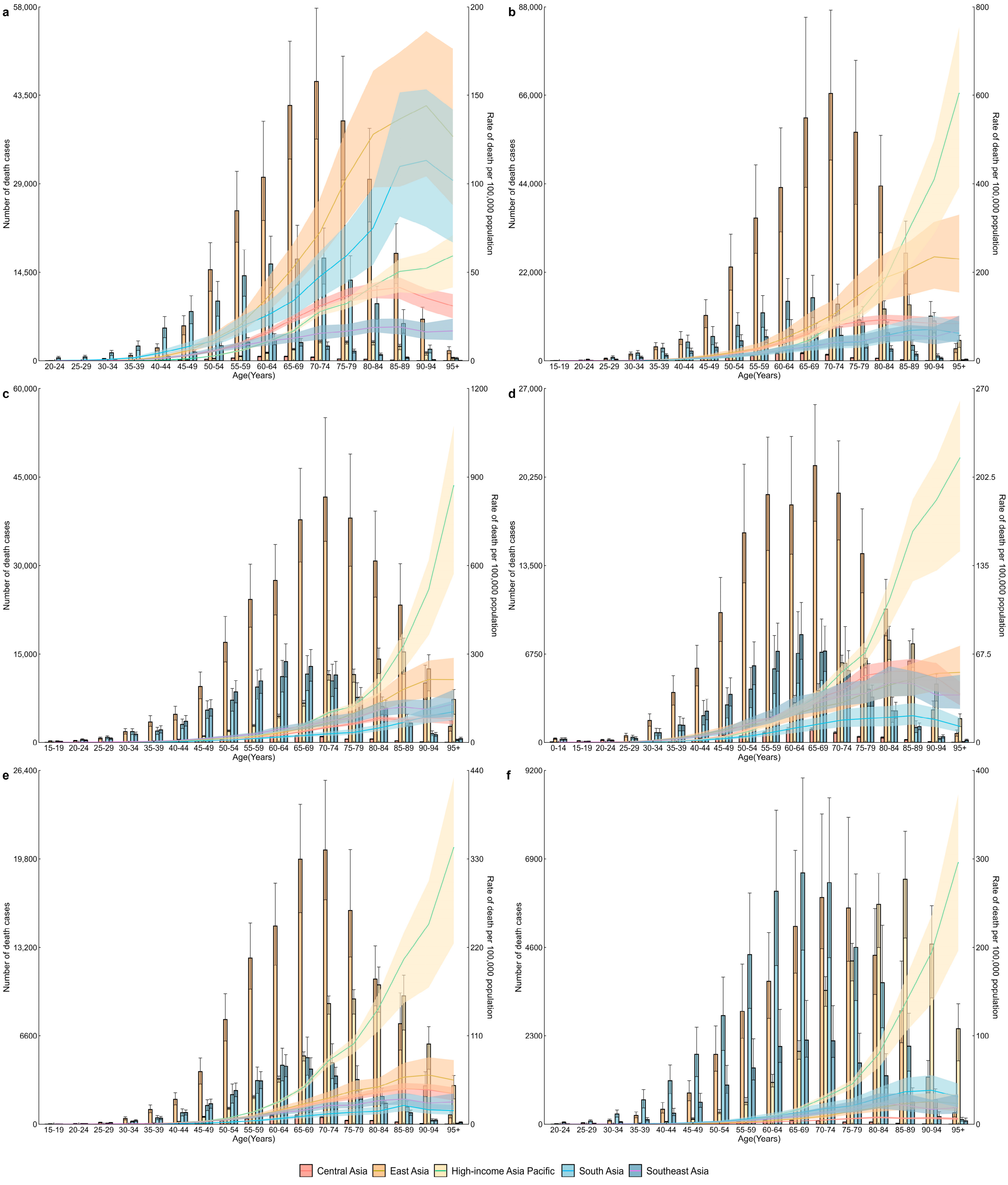

Figure S7. Deaths and age-specific death rates for esophageal (a), stomach (b), colorectal (c), liver (d), pancreatic (e), and gallbladder and biliary tract (f) cancers by age group in five GBD subregions of Asia, 2023.

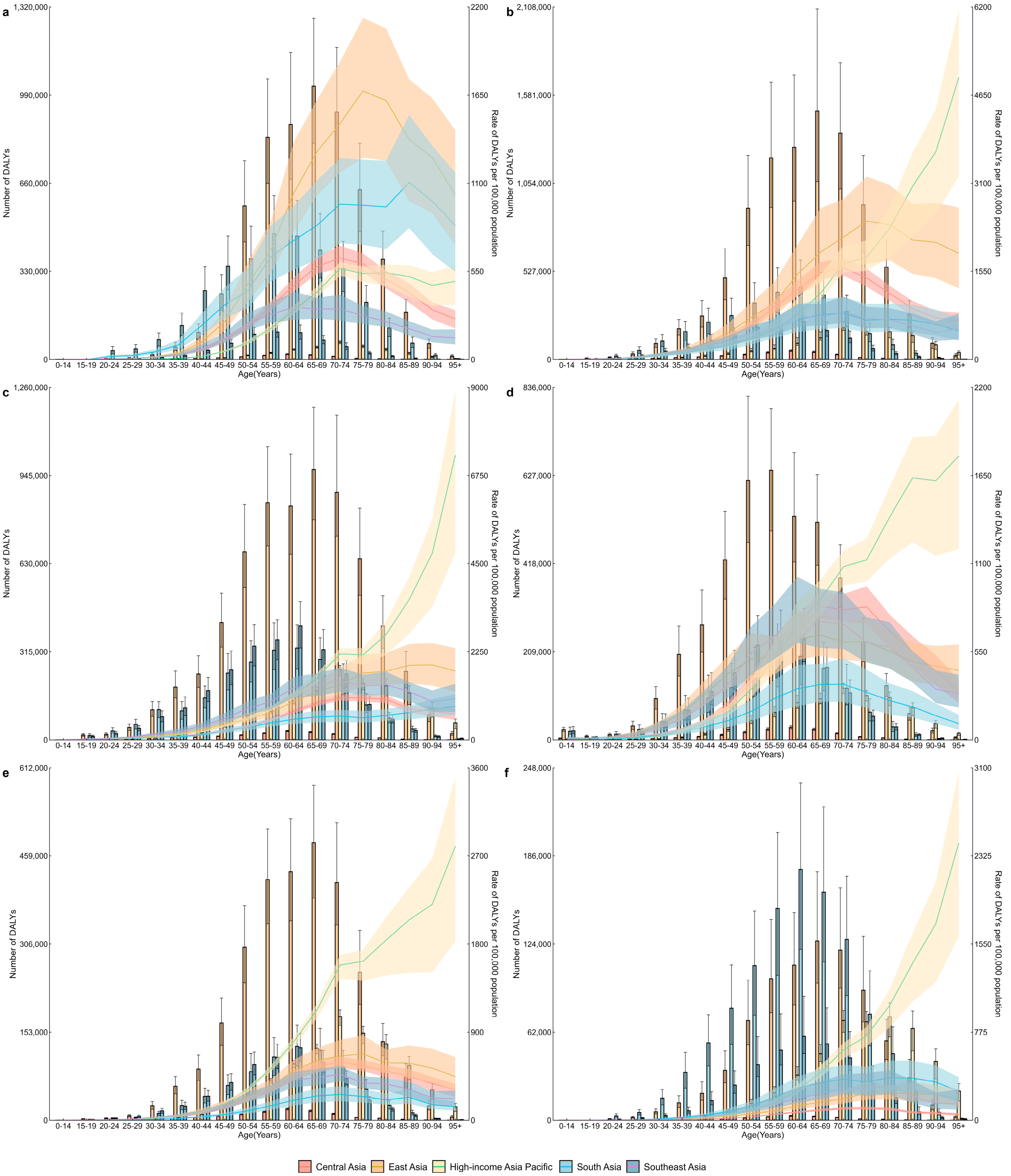

Figure S8. DALYs and age-specific DALY rates for esophageal (a), stomach (b), colorectal (c), liver (d), pancreatic (e), and gallbladder and biliary tract (f) cancers by age group in five GBD subregions of Asia, 2023. DALYs: disability-adjusted life years.

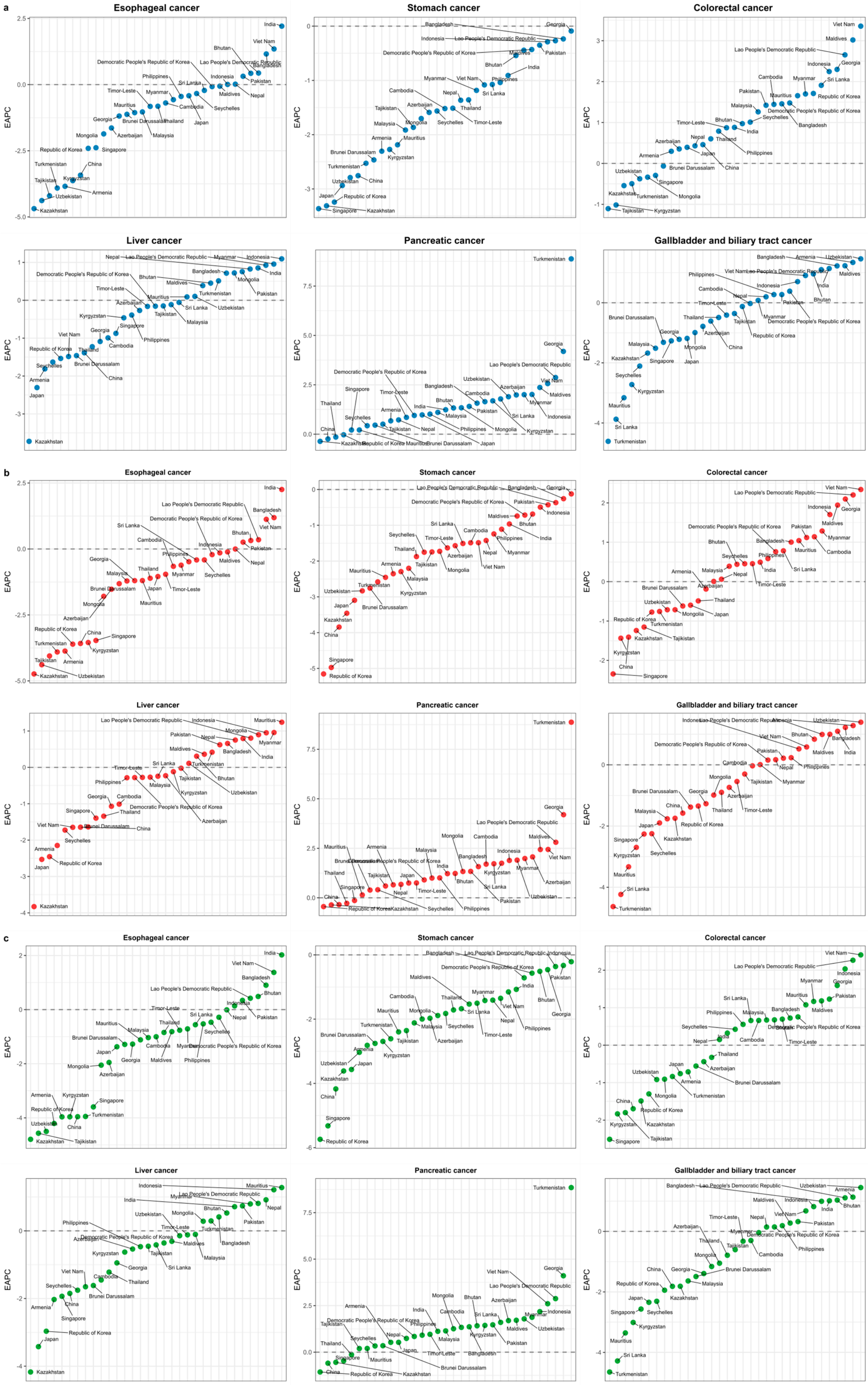

Figure S9. The EAPCs in the age-standardized incidence (a), death (b), and DALY (c) rates for gastrointestinal cancers across Asian countries and regions, 1990–2023. EAPCs: estimated annual percent changes; DALY: disability-adjusted life years.

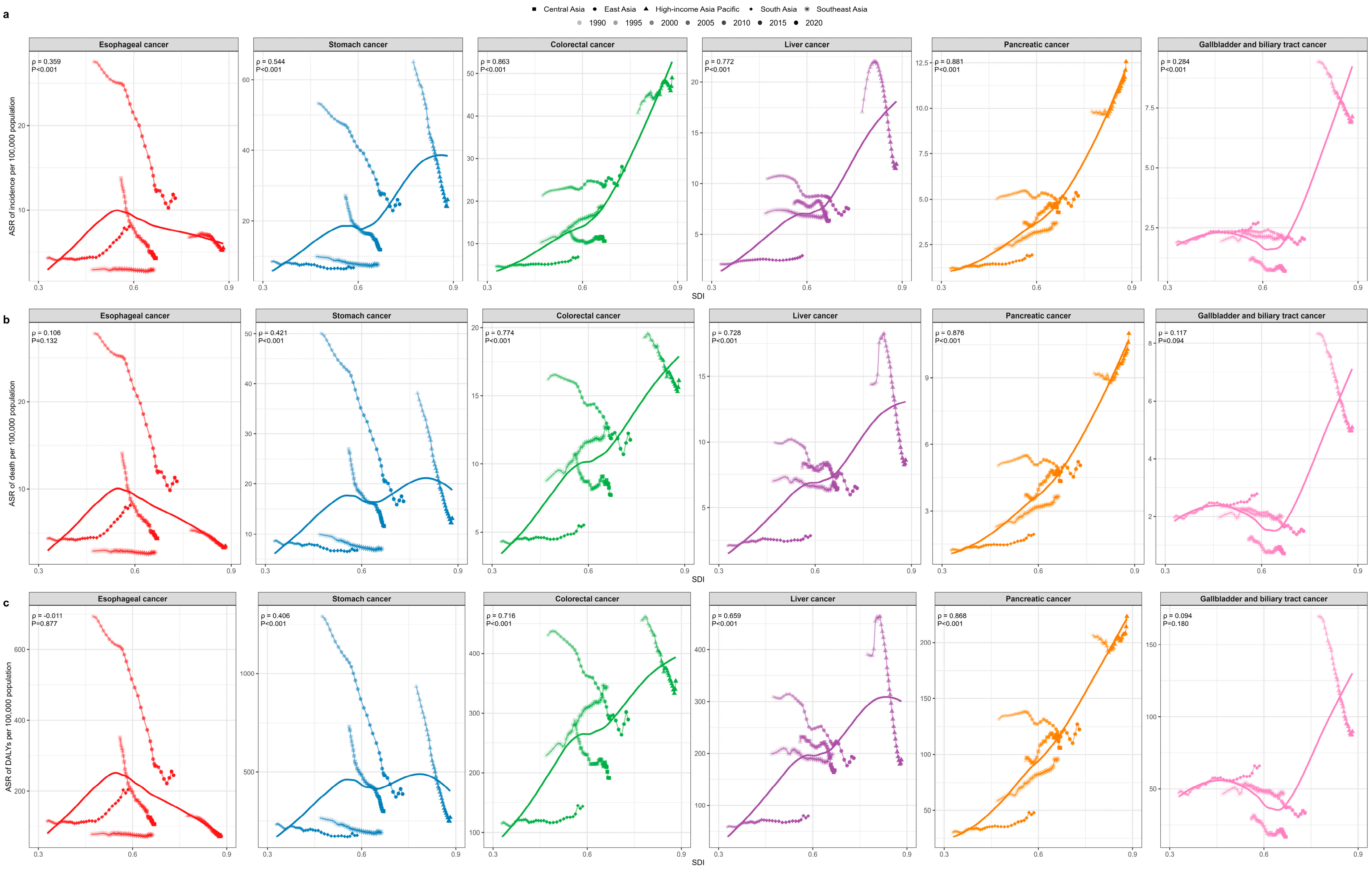

Figure S10. Temporal trends in age-standardized incidence(a), death(b), and DALY(c) rates of gastrointestinal cancers by SDI in five GBD subregions of Asia, 1990–2023. DALY: disability-adjusted life year; SDI: Socio-demographic Index.

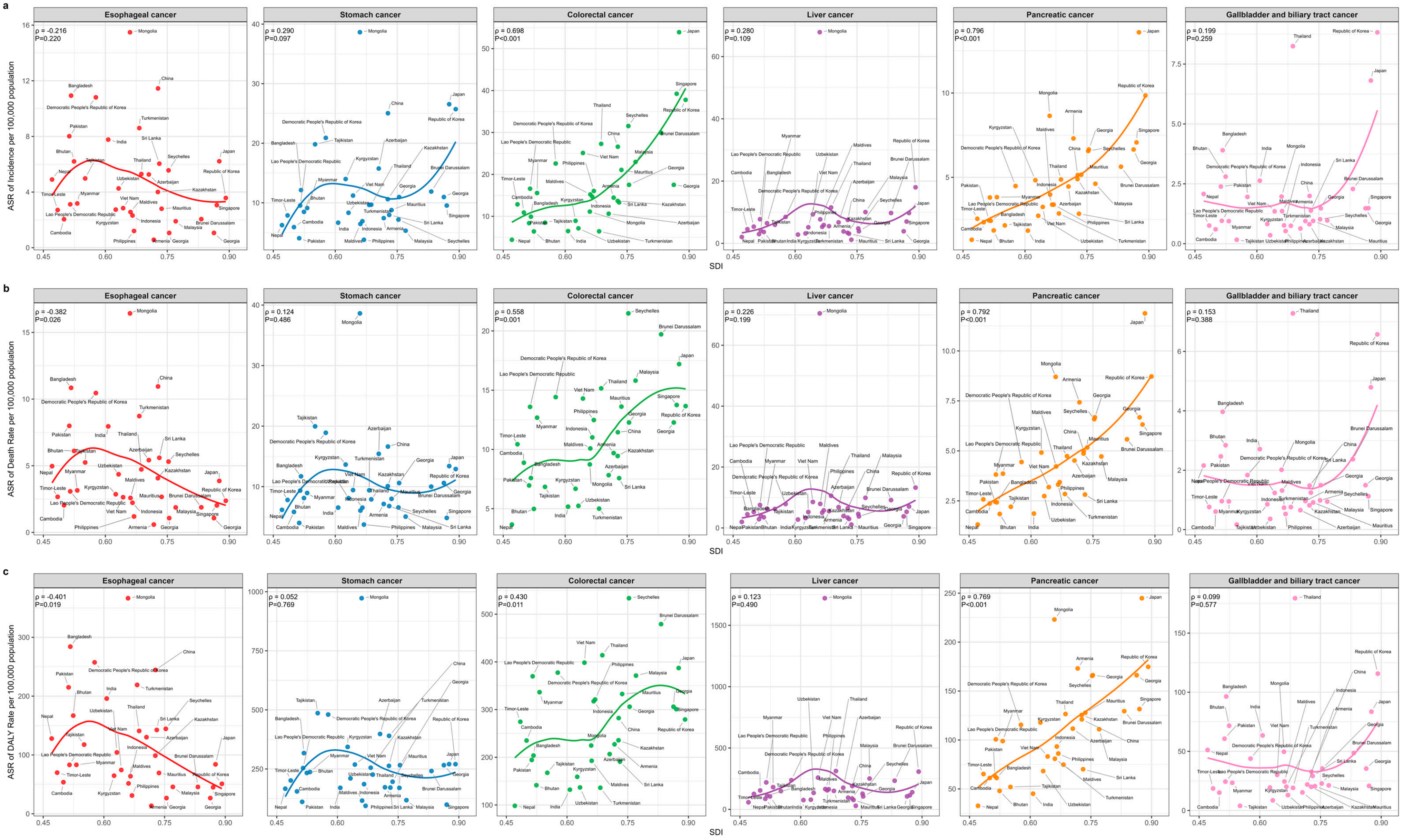

Figure S11. Associations between SDI and age-standardized incidence (a), death (b), and DALY (c) rates of gastrointestinal cancers across Asian countries and regions, 2023. SDI: Socio-demographic Index; DALY: disability-adjusted life year.

Table S1. Geographic subregions and countries/regions included in the study.

GBD subregion	Number of countries/regions	Countries/Regions
Central Asia	9	Armenia, Azerbaijan, Georgia, Kazakhstan, Kyrgyzstan, Mongolia, Tajikistan, Turkmenistan, Uzbekistan
East Asia*	2	China, Democratic People's Republic of Korea
High-income Asia Pacific	4	Brunei Darussalam, Japan, Republic of Korea, Singapore
South Asia	5	Bangladesh, Bhutan, India, Nepal, Pakistan
Southeast Asia	13	Cambodia, Indonesia, Lao People's Democratic Republic, Malaysia, Maldives, Mauritius, Myanmar, Philippines, Seychelles, Sri Lanka, Thailand, Timor-Leste, Viet Nam

*Estimates for Taiwan (Province of China) were included in the East Asian subregional analysis but were not presented separately in the country and territory level analyses.

Table S2. Deaths, age-standardized rates, and changes of gastrointestinal cancers in five GBD subregions of Asia, 1990–2023

	1990 Death (95% UI)	2023 Death (95% UI)	1990 ASDR Per 100 000 (95% UI)	2023 ASDR Per 100 000 (95% UI)	1990 to 2023 EAPC (95% CI)
**Central Asia**					
EC	6247 (5887 to 6537)	3789 (3548 to 4108)	19.95 (18.50 to 21.29)	4.37 (4.09 to 4.75)	-3.67 (-3.86 to -3.47)
SC	12,337 (11,447 to 12,951)	10,249 (9428 to 11,255)	26.96 (25.04 to 28.38)	11.57 (10.67 to 12.68)	-2.34 (-2.47 to -2.21)
CRC	4871 (4606 to 5131)	6565 (6174 to 6975)	10.76 (10.16 to 11.36)	7.70 (7.23 to 8.23)	-0.61 (-0.79 to -0.44)
LC	3727 (3244 to 4192)	5691 (5161 to 6336)	8.04 (6.99 to 9.03)	6.45 (5.83 to 7.21)	-0.74 (-0.93 to -0.55)
PC	1653 (1458 to 1848)	3749 (3398 to 4094)	3.66 (3.22 to 4.11)	4.33 (3.93 to 4.75)	1.03 (0.81 to 1.25)
GBTC	522 (460 to 596)	606 (542 to 684)	1.20 (1.05 to 1.37)	0.72 (0.64 to 0.81)	-1.58 (-1.96 to -1.20)
**East Asia**					
EC	240,667 (173,059 to 282,288)	261,368 (223,691 to 299,941)	27.78 (20.36 to 32.54)	10.88 (9.34 to 12.43)	-3.52 (-3.85 to -3.18)
SC	435,720 (356,140 to 538,806)	392,100 (310,498 to 492,699)	50.04 (41.14 to 62.12)	16.54 (13.12 to 20.72)	-3.81 (-4.07 to -3.54)
CRC	138,849 (118,999 to 161,020)	273,848 (240,026 to 320,808)	16.21 (13.88 to 18.70)	11.77 (10.36 to 13.72)	-1.34 (-1.48 to -1.20)
LC	94,080 (82,102 to 109,885)	149,347 (132,132 to 169,467)	9.95 (8.66 to 11.61)	6.48 (5.78 to 7.37)	-1.56 (-1.73 to -1.39)
PC	44,177 (38,322 to 51,315)	120,635 (106,516 to 137,340)	5.04 (4.37 to 5.87)	5.05 (4.45 to 5.76)	-0.30 (-0.43 to -0.16)
GBTC	19,310 (15,348 to 25,065)	35,603 (28,869 to 46,988)	2.34 (1.86 to 2.99)	1.51 (1.22 to 1.98)	-1.60 (-1.80 to -1.40)
**High-income Asia Pacific**					

EC	10,684 (10,017 to 11,257)	17,853 (15,768 to 19,387)	5.33 (4.98 to 5.62)	3.47 (3.15 to 3.75)	-1.56 (-1.66 to -1.47)
SC	74,776 (68,677 to 78,796)	74,535 (60,625 to 87,961)	38.15 (35.13 to 40.24)	13.13 (11.15 to 15.72)	-3.52 (-3.60 to -3.44)
CRC	37,272 (34,350 to 39,050)	90,336 (75,288 to 100,285)	19.28 (17.61 to 20.31)	16.12 (14.11 to 17.59)	-0.74 (-0.79 to -0.69)
LC	29,259 (26,401 to 32,146)	45,259 (39,065 to 50,570)	14.39 (12.99 to 15.82)	8.63 (7.57 to 9.68)	-2.25 (-2.72 to -1.77)
PC	18,121 (16,973 to 18,884)	59,809 (50,065 to 65,801)	9.21 (8.56 to 9.62)	11.00 (9.63 to 11.91)	0.46 (0.35 to 0.58)
GBTC	16,075 (14,900 to 17,206)	31,105 (25,218 to 35,425)	8.33 (7.65 to 8.91)	5.08 (4.28 to 5.71)	-1.76 (-1.81 to -1.70)
**South Asia**					
EC	24,682 (18,965 to 31,913)	122,202 (94,809 to 156,867)	4.35 (3.34 to 5.64)	8.14 (6.34 to 10.40)	1.80 (1.34 to 2.27)
SC	49,841 (36,082 to 68,010)	103,877 (75,369 to 138,240)	8.61 (6.24 to 11.66)	6.73 (4.89 to 9.01)	-0.84 (-0.96 to -0.71)
CRC	24,431 (18,912 to 30,840)	83,110 (66,603 to 100,690)	4.29 (3.31 to 5.39)	5.51 (4.42 to 6.73)	0.60 (0.46 to 0.74)
LC	12,479 (8412 to 17,063)	45,322 (31,261 to 62,219)	2.07 (1.41 to 2.84)	2.86 (1.98 to 3.94)	0.76 (0.61 to 0.92)
PC	6721 (4998 to 8400)	29,567 (22,604 to 36,829)	1.23 (0.90 to 1.54)	1.95 (1.49 to 2.43)	1.24 (1.08 to 1.40)
GBTC	10,426 (7857 to 13,987)	41,206 (30,050 to 54,375)	1.97 (1.49 to 2.66)	2.78 (2.02 to 3.67)	1.01 (0.90 to 1.12)
**Southeast Asia**					
EC	7483 (5856 to 9378)	20,218 (15,957 to 25,129)	2.92 (2.30 to 3.67)	2.76 (2.18 to 3.40)	-0.34 (-0.42 to -0.26)
SC	25,434 (18,909 to 32,012)	50,242 (39,487 to 66,469)	9.96 (7.40 to 12.69)	6.99 (5.49 to 9.22)	-1.20 (-1.30 to -1.11)
CRC	21,917 (17,834 to 27,893)	89,887 (73,297 to 107,966)	8.75 (7.14 to 11.18)	12.64 (10.32 to 15.10)	1.11 (1.04 to 1.18)
LC	18,733 (14,533 to 24,294)	50,146 (40,037 to 63,915)	7.01 (5.47 to 9.06)	6.82 (5.45 to 8.69)	-0.37 (-0.45 to -0.29)
PC	5718 (4533 to 6933)	26,107 (21,743 to 31,026)	2.29 (1.81 to 2.80)	3.64 (3.02 to 4.33)	1.35 (1.28 to 1.41)
GBTC	4679 (3630 to 6225)	13,656 (11,050 to 19,400)	1.96 (1.51 to 2.67)	1.99 (1.61 to 2.83)	-0.18 (-0.33 to -0.04)

EC: esophageal cancer; SC: stomach cancer; CRC: colon and rectum cancer; LC: liver cancer; PC: pancreatic cancer; GBTC: gallbladder and biliary tract cancer; ASDR: age-standardized death rate; EAPC: estimated annual percentage change

Table S3. DALYs, age-standardized rates, and changes of gastrointestinal cancers in five GBD subregions of Asia, 1990–2023

	1990 DALYs (95% UI)	2023 DALYs (95% UI)	1990 ASDALYR Per 100 000 (95% UI)	2023 ASDALYR Per 100 000 (95% UI)	1990 to 2023 EAPC (95% CI)
**Central Asia**					
EC	168,012 (158,182 to 175,942)	100,458 (94,260 to 108,741)	351.22 (330.46 to 367.84)	106.57 (99.87 to 115.04)	-3.79 (-3.99 to -3.60)
SC	362,026 (336,395 to 380,326)	286,687 (264,479 to 316,150)	731.88 (680.14 to 768.76)	299.05 (275.73 to 328.94)	-2.61 (-2.72 to -2.50)

CRC	142,461 (134,726 to 149,356)	179,079 (167,850 to 189,353)	288.09 (272.56 to 302.52)	191.25 (179.61 to 202.79)	-1.00 (-1.13 to -0.86)
LC	114,130 (98,933 to 128,553)	160,168 (144,580 to 178,008)	222.33 (193.61 to 250.76)	167.47 (151.34 to 186.20)	-1.03 (-1.21 to -0.84)
PC	46,143 (41,016 to 51,481)	99,838 (90,187 to 108,999)	94.83 (84.12 to 105.87)	105.70 (95.58 to 115.51)	0.75 (0.54 to 0.95)
GBTC	13,629 (12,053 to 15,477)	15,465 (13,784 to 17,429)	28.94 (25.59 to 32.84)	16.68 (14.90 to 18.80)	-1.79 (-2.15 to -1.43)
**East Asia**					
EC	6,482,794 (4,575,837 to 7,572,522)	5,920,498 (5,269,283 to 6,748,331)	692.65 (494.42 to 811.51)	244.41 (217.21 to 277.63)	-3.87 (-4.22 to -3.52)
SC	12,356,078 (10,015,805 to 15,247,415)	9,166,431 (7,259,020 to 11,594,254)	1289.83 (1052.46 to 1584.53)	388.47 (307.78 to 490.69)	-4.12 (-4.39 to -3.86)
CRC	4,228,429 (3,579,181 to 4,896,813)	6,650,054 (5,936,268 to 7,828,709)	430.49 (364.00 to 497.28)	289.26 (258.42 to 339.91)	-1.62 (-1.76 to -1.48)
LC	3,173,440 (2,770,894 to 3,693,095)	4,214,674 (3,702,693 to 4,819,913)	308.77 (269.82 to 360.14)	190.83 (167.99 to 218.31)	-1.77 (-1.93 to -1.61)
PC	1,268,280 (1,103,370 to 1,479,995)	2,900,243 (2,580,770 to 3,281,332)	131.71 (114.42 to 153.28)	122.39 (108.42 to 137.98)	-0.54 (-0.68 to -0.41)
GBTC	513,331 (408,527 to 668,616)	790,510 (655,899 to 1,048,775)	55.54 (44.27 to 71.92)	33.28 (27.61 to 44.20)	-1.84 (-2.03 to -1.65)
**High-income Asia Pacific**					
EC	267,546 (252,303 to 281,927)	328,144 (298,252 to 354,184)	129.60 (122.01 to 136.63)	74.38 (68.69 to 79.72)	-2.00 (-2.13 to -1.87)
SC	1,890,826 (1,698,202 to 1,992,548)	1,220,372 (1,045,184 to 1,462,308)	933.86 (841.49 to 983.75)	267.70 (237.12 to 327.56)	-4.07 (-4.16 to -3.99)
CRC	918,197 (863,579 to 960,469)	1,586,078 (1,403,648 to 1,720,368)	455.11 (426.10 to 476.64)	353.44 (319.10 to 380.63)	-1.00 (-1.05 to -0.94)
LC	806,425 (715,808 to 899,924)	803,244 (704,857 to 900,790)	390.81 (346.12 to 436.84)	187.43 (165.01 to 210.46)	-2.93 (-3.40 to -2.46)
PC	420,618 (395,155 to 439,401)	1,014,599 (895,559 to 1,093,154)	206.53 (193.58 to 215.84)	223.15 (202.78 to 237.19)	0.14 (0.03 to 0.24)
GBTC	340,841 (318,868 to 364,247)	459,972 (390,478 to 514,092)	169.42 (158.47 to 181.37)	89.67 (78.20 to 99.81)	-2.21 (-2.27 to -

					2.14)
**South Asia**					
EC	737,979 (561,209 to 955,034)	3,357,367 (2,617,990 to 4,288,886)	114.55 (87.56 to 148.59)	203.97 (159.22 to 261.06)	1.63 (1.18 to 2.09)
SC	1,548,403 (1,117,384 to 2,115,664)	2,975,307 (2,180,367 to 3,910,788)	233.47 (168.81 to 317.18)	177.63 (129.76 to 234.03)	-0.95 (-1.08 to -0.82)
CRC	763,779 (589,796 to 961,477)	2,391,075 (1,899,598 to 2,907,211)	115.32 (89.18 to 145.16)	143.57 (114.54 to 174.26)	0.47 (0.32 to 0.63)
LC	407,086 (276,070 to 555,017)	1,348,756 (929,946 to 1,845,939)	58.63 (39.62 to 80.15)	79.63 (54.87 to 109.12)	0.70 (0.55 to 0.85)
PC	189,240 (141,591 to 237,300)	782,332 (588,554 to 977,418)	30.54 (22.80 to 38.24)	47.95 (36.21 to 59.85)	1.18 (0.99 to 1.36)
GBTC	288,052 (217,078 to 383,655)	1,061,654 (775,522 to 1,403,808)	47.33 (35.62 to 63.36)	65.89 (48.16 to 86.90)	0.94 (0.83 to 1.05)
**Southeast Asia**					
EC	221,959 (173,197 to 278,855)	589,200 (468,355 to 739,397)	77.92 (60.87 to 97.53)	75.97 (60.30 to 95.02)	-0.24 (-0.32 to -0.15)
SC	770,155 (566,386 to 977,521)	1,472,879 (1,150,902 to 1,935,249)	264.59 (196.17 to 333.48)	192.21 (150.23 to 252.73)	-1.08 (-1.18 to -0.98)
CRC	662,124 (533,812 to 846,827)	2,611,966 (2,119,996 to 3,142,278)	228.55 (185.89 to 290.32)	343.15 (279.28 to 411.24)	1.25 (1.19 to 1.31)
LC	608,136 (466,557 to 795,065)	1,530,859 (1,207,582 to 1,968,805)	199.25 (153.69 to 259.39)	198.06 (156.71 to 254.04)	-0.32 (-0.41 to -0.23)
PC	164,533 (130,395 to 201,212)	732,328 (606,279 to 872,051)	58.52 (46.46 to 71.07)	95.86 (79.81 to 114.18)	1.44 (1.38 to 1.51)
GBTC	124,200 (96,801 to 162,624)	346,274 (280,549 to 494,017)	46.63 (36.24 to 61.50)	46.80 (37.82 to 66.51)	-0.23 (-0.39 to -0.08)

EC: esophageal cancer; SC: stomach cancer; CRC: colon and rectum cancer; LC: liver cancer; PC: pancreatic cancer; GBTC: gallbladder and biliary tract cancer; DALY: disability-adjusted life year; ASDALYR : age-standardized disability-adjusted life year rate; EAPC: estimated annual percentage change

Table S4. Incidence, age-standardized rates, and changes of gastrointestinal cancers by sex in five GBD subregions of Asia, 1990-2023

		1990 Incidence (95% UI)	2023 Incidence (95% UI)	1990 ASIR Per 100 000 (95% UI)	2023 ASIR Per 100 000 (95% UI)	1990 to 2023 EAPC (95% CI)
**Central Asia**						
EC	Male	3513 (3263 to 3738)	2350 (2165 to 2582)	19.20 (17.80 to 20.49)	6.02 (5.56 to 6.69)	-3.60 (-3.86 to -3.35)
	Female	2764 (2564 to 2951)	1487 (1369 to 1715)	10.32 (9.55 to 11.03)	3.00 (2.76 to 3.46)	-3.88 (-4.03 to -3.73)
SC	Male	7829 (7087 to 8389)	7403 (6698 to 8485)	40.35 (36.58 to 43.29)	18.27 (16.51 to 20.74)	-2.17 (-2.34 to -2.00)
	Female	4954 (4591 to 5334)	3462 (3146 to 3787)	18.27 (16.96 to 19.67)	6.98 (6.30 to 7.62)	-2.71 (-2.83 to -2.60)
CRC	Male	2987 (2733 to 3229)	5245 (4737 to 5762)	15.44 (14.17 to 16.61)	13.24 (11.93 to 14.48)	0.02 (-0.25 to 0.28)
	Female	3192 (2907 to 3488)	4264 (3745 to 4764)	11.68 (10.64 to 12.72)	8.60 (7.58 to 9.59)	-0.50 (-0.67 to -0.33)
LC	Male	2320 (1986 to 2713)	3531 (3135 to 4044)	11.83 (10.13 to 13.84)	8.54 (7.62 to 9.78)	-1.14 (-1.26 to -1.02)
	Female	1498 (1250 to 1757)	2261 (1950 to 2624)	5.44 (4.55 to 6.40)	4.61 (3.96 to 5.37)	-0.41 (-0.70 to -0.12)
PC	Male	857 (722 to 1015)	2169 (1891 to 2459)	4.40 (3.70 to 5.20)	5.45 (4.75 to 6.19)	1.16 (0.94 to 1.39)
	Female	828 (708 to 978)	1639 (1410 to 1877)	3.08 (2.62 to 3.63)	3.35 (2.87 to 3.85)	0.78 (0.56 to 0.99)
GBTC	Male	183 (155 to 214)	273 (228 to 322)	1.02 (0.86 to 1.19)	0.71 (0.59 to 0.84)	-1.19 (-1.58 to -0.80)
	Female	335 (279 to 396)	336 (280 to 398)	1.25 (1.04 to 1.48)	0.69 (0.57 to 0.82)	-1.81 (-2.18 to -1.43)
**East Asia**						
EC	Male	168,798 (124,761 to 208,616)	213,199 (183,937 to 249,072)	38.43 (29.10 to 47.85)	18.37 (15.91 to 21.38)	-2.79 (-3.09 to -2.49)
	Female	77,521 (38,482 to 103,032)	62,085 (47,171 to 75,810)	17.29 (8.83 to 23.02)	4.90 (3.71 to 5.97)	-4.81 (-5.21 to -4.40)
SC	Male	316,262 (241,708 to 416,361)	431,510 (305,504 to 586,090)	71.69 (54.51 to 95.94)	37.54 (26.38 to 50.96)	-2.28 (-2.46 to -2.10)
	Female	165,800 (134,896 to 212,230)	157,584 (121,586 to 205,614)	36.14 (29.31 to 46.70)	12.86 (9.98 to 16.50)	-3.75 (-4.01 to -3.48)
CRC	Male	108,303 (88,369 to 131,860)	414,439 (338,131 to 507,003)	24.83 (20.27 to 30.79)	36.42 (29.83 to 44.82)	0.99 (0.87 to 1.12)
	Female	85,070 (64,816 to 109,039)	231,932 (178,387 to 315,763)	18.43 (14.01 to 23.67)	18.62 (14.36 to 25.31)	-0.32 (-0.43 to -0.21)
LC	Male	71,170 (60,024 to 84,789)	127,823 (108,503 to 151,288)	14.40 (12.09 to 17.29)	11.49 (9.85 to 13.48)	-0.96 (-1.11 to -0.82)
	Female	30,822 (24,263 to 39,658)	43,825 (34,372 to 53,426)	6.50 (5.11 to 8.44)	3.66 (2.90 to 4.39)	-2.13 (-2.32 to -1.94)
PC	Male	27,110 (22,400 to 34,264)	75,696 (64,916 to 88,595)	6.10 (5.02 to 7.74)	6.60 (5.68 to 7.71)	0.03 (-0.09 to 0.15)
	Female	18,372 (15,042 to 22,687)	48,017 (39,309 to 60,278)	4.03 (3.29 to 4.99)	3.83 (3.15 to 4.77)	-0.56 (-0.71 to -0.40)
GBTC	Male	9627 (7316 to 13,642)	25,471 (19,278 to 37,851)	2.33 (1.76 to 3.22)	2.27 (1.71 to 3.34)	-0.21 (-0.36 to -0.06)
	Female	10,605 (7404 to 14,348)	22,400 (15,880 to 31,581)	2.37 (1.64 to 3.23)	1.79 (1.27 to 2.53)	-1.14 (-1.31 to -0.98)
**High-income Asia Pacific**						
EC	Male	11,226 (10,402 to 12,009)	21,827 (19,823 to 23,711)	12.71 (11.85 to 13.62)	10.03 (9.11 to 10.91)	-0.97 (-1.20 to -0.74)
	Female	2473 (2024 to 2858)	4764 (3719 to 5620)	2.18 (1.77 to 2.52)	1.69 (1.43 to 1.96)	-0.87 (-0.94 to -0.79)

SC	Male	83,713 (76,501 to 89,408)	84,034 (74,794 to 101,188)	96.70 (89.01 to 103.86)	38.28 (34.08 to 47.10)	-2.93 (-3.03 to -2.83)
	Female	46,719 (42,682 to 50,166)	48,865 (35,879 to 61,528)	41.69 (38.02 to 44.69)	16.26 (12.92 to 20.38)	-3.30 (-3.42 to -3.18)
CRC	Male	45,857 (42,476 to 49,195)	136,255 (122,718 to 149,150)	53.49 (49.67 to 57.24)	64.88 (58.69 to 70.95)	0.37 (0.27 to 0.46)
	Female	35,363 (32,015 to 38,381)	100,496 (79,267 to 115,828)	31.21 (28.20 to 33.89)	35.32 (29.63 to 39.87)	0.22 (0.16 to 0.28)
LC	Male	26,017 (23,085 to 29,306)	40,242 (35,816 to 44,968)	28.22 (25.08 to 31.75)	18.97 (16.83 to 21.26)	-1.96 (-2.36 to -1.57)
	Female	8796 (7560 to 10,098)	18,582 (14,079 to 22,132)	7.74 (6.64 to 8.91)	5.87 (4.62 to 6.87)	-1.70 (-2.24 to -1.17)
PC	Male	10,805 (10,302 to 11,307)	33,085 (29,956 to 35,274)	12.83 (12.18 to 13.42)	14.92 (13.64 to 15.86)	0.38 (0.27 to 0.48)
	Female	8655 (7671 to 9337)	34,531 (26,209 to 39,691)	7.55 (6.68 to 8.16)	10.41 (8.39 to 11.68)	1.01 (0.88 to 1.13)
GBTC	Male	7946 (7305 to 8614)	22,041 (19,653 to 25,031)	9.89 (9.13 to 10.66)	9.37 (8.38 to 10.75)	-0.33 (-0.38 to -0.29)
	Female	10,405 (9025 to 11,410)	20,294 (14,319 to 24,842)	9.08 (7.86 to 9.97)	5.41 (4.08 to 6.44)	-1.71 (-1.76 to -1.66)
**South Asia**						
EC	Male	13,696 (9554 to 18,155)	66,305 (47,429 to 89,931)	4.38 (3.04 to 5.82)	8.60 (6.12 to 11.73)	2.05 (1.64 to 2.47)
	Female	11,816 (8085 to 17,529)	58,566 (39,419 to 82,439)	4.19 (2.89 to 6.27)	7.43 (5.07 to 10.44)	1.49 (0.96 to 2.03)
SC	Male	33,415 (20,636 to 50,541)	68,933 (40,178 to 99,494)	10.66 (6.51 to 16.09)	8.81 (5.11 to 12.79)	-0.68 (-0.84 to -0.52)
	Female	18,761 (12,204 to 30,724)	40,758 (25,513 to 61,300)	6.33 (4.08 to 10.46)	5.04 (3.18 to 7.53)	-0.79 (-0.92 to -0.67)
CRC	Male	14,035 (10,651 to 19,737)	53,971 (39,458 to 72,050)	4.55 (3.38 to 6.42)	7.00 (5.13 to 9.36)	1.17 (1.01 to 1.33)
	Female	14,215 (9900 to 19,435)	55,349 (41,745 to 72,717)	4.83 (3.35 to 6.52)	6.85 (5.17 to 8.99)	0.81 (0.61 to 1.02)
LC	Male	8993 (5494 to 13,438)	31,785 (18,670 to 48,771)	2.71 (1.66 to 4.08)	3.91 (2.32 to 6.05)	0.88 (0.72 to 1.03)
	Female	4056 (2392 to 6579)	15,580 (8969 to 24,259)	1.39 (0.81 to 2.24)	1.93 (1.11 to 3.01)	0.94 (0.81 to 1.07)
PC	Male	4134 (2738 to 5659)	17,165 (12,027 to 23,530)	1.36 (0.89 to 1.87)	2.23 (1.54 to 3.04)	1.33 (1.17 to 1.49)
	Female	2715 (1803 to 3907)	12,851 (9160 to 17,167)	1.02 (0.68 to 1.47)	1.65 (1.17 to 2.21)	1.26 (1.05 to 1.47)
GBTC	Male	3992 (2513 to 5931)	15,144 (9650 to 22,133)	1.40 (0.88 to 2.06)	2.04 (1.30 to 2.98)	1.16 (1.03 to 1.29)
	Female	6481 (4500 to 9344)	26,268 (17,104 to 38,172)	2.41 (1.66 to 3.48)	3.35 (2.19 to 4.87)	0.90 (0.79 to 1.02)
**Southeast Asia**						
EC	Male	5202 (3705 to 6844)	16,675 (13,044 to 21,342)	4.15 (2.96 to 5.47)	4.68 (3.65 to 6.01)	0.22 (0.11 to 0.33)
	Female	2593 (1756 to 3783)	5122 (4025 to 7140)	1.86 (1.26 to 2.66)	1.34 (1.06 to 1.86)	-1.25 (-1.35 to -1.15)
SC	Male	16,808 (11,108 to 22,840)	37,667 (27,532 to 50,932)	13.56 (8.97 to 18.54)	10.85 (7.94 to 14.70)	-0.81 (-0.90 to -0.71)
	Female	10,000 (7050 to 13,695)	19,428 (14,652 to 26,123)	6.99 (4.94 to 9.52)	5.03 (3.80 to 6.75)	-1.16 (-1.27 to -1.06)
CRC	Male	15,653 (11,757 to 22,091)	83,044 (62,563 to 103,435)	12.56 (9.40 to 17.69)	23.90 (18.10 to 29.78)	1.95 (1.87 to 2.04)
	Female	11,724 (9179 to 15,287)	55,956 (42,434 to 71,019)	8.29 (6.47 to 10.87)	14.37 (10.94 to 18.22)	1.60 (1.54 to 1.66)
LC	Male	14,512 (10,636 to 19,927)	40,851 (31,556 to 55,137)	10.90 (8.02 to 15.05)	11.37 (8.78 to 15.26)	-0.24 (-0.32 to -0.15)
	Female	5128 (3500 to 7641)	12,985 (9009 to 18,738)	3.64 (2.52 to 5.32)	3.40 (2.38 to 4.89)	-0.50 (-0.58 to -0.43)

PC	Male	3429 (2435 to 4383)	15,261 (12,123 to 19,181)	2.76 (1.96 to 3.51)	4.39 (3.48 to 5.51)	1.34 (1.26 to 1.41)
	Female	2413 (1740 to 3205)	11,636 (8991 to 14,753)	1.79 (1.29 to 2.37)	3.02 (2.33 to 3.83)	1.54 (1.49 to 1.60)
GBTC	Male	2627 (1781 to 3758)	9061 (6894 to 14,360)	2.27 (1.53 to 3.26)	2.78 (2.13 to 4.45)	0.43 (0.27 to 0.59)
	Female	2167 (1525 to 3091)	6025 (4821 to 10,080)	1.64 (1.15 to 2.36)	1.60 (1.28 to 2.68)	-0.34 (-0.48 to -0.20)

EC: esophageal cancer; SC: stomach cancer; CRC: colon and rectum cancer; LC: liver cancer; PC: pancreatic cancer; GBTC: gallbladder and biliary tract cancer; ASIR: age-standardized incidence rate; EAPC: estimated annual percent change; UI: uncertainty interval; CI: confidence interval

Table S5. Deaths, age-standardized rates, and changes of gastrointestinal cancers by sex in five GBD subregions of Asia, 1990-2023

		1990 Death (95% UI)	2023 Death (95% UI)	1990 ASDR Per 100 000 (95% UI)	2023 ASDR Per 100 000 (95% UI)	1990 to 2023 EAPC (95% CI)
**Central Asia**						
EC	Male	3478 (3230 to 3706)	2332 (2147 to 2581)	10.50 (9.69 to 11.22)	6.24 (5.73 to 6.95)	-3.60 (-3.85 to -3.34)
	Female	2768 (2564 to 2953)	1457 (1340 to 1679)	14.11 (13.30 to 14.79)	3.01 (2.76 to 3.47)	-3.92 (-4.06 to -3.77)
SC	Male	7454 (6754 to 7985)	6947 (6239 to 7880)	40.19 (36.40 to 43.20)	17.86 (16.07 to 20.17)	-2.21 (-2.38 to -2.05)
	Female	4883 (4521 to 5245)	3302 (3023 to 3601)	18.27 (16.92 to 19.65)	6.81 (6.19 to 7.44)	-2.76 (-2.88 to -2.64)
CRC	Male	2330 (2164 to 2475)	3611 (3333 to 3856)	12.87 (11.93 to 13.63)	9.76 (9.02 to 10.41)	-0.43 (-0.65 to -0.21)
	Female	2541 (2348 to 2747)	2954 (2676 to 3265)	9.48 (8.77 to 10.26)	6.19 (5.62 to 6.86)	-0.91 (-1.06 to -0.76)
LC	Male	2233 (1907 to 2612)	3408 (3031 to 3899)	11.92 (10.21 to 13.97)	8.56 (7.61 to 9.80)	-1.19 (-1.31 to -1.07)
	Female	1494 (1246 to 1754)	2283 (1973 to 2631)	5.51 (4.61 to 6.48)	4.76 (4.09 to 5.50)	-0.36 (-0.65 to -0.06)
PC	Male	823 (693 to 976)	2114 (1843 to 2397)	4.40 (3.70 to 5.22)	5.51 (4.81 to 6.28)	1.22 (0.99 to 1.45)
	Female	830 (707 to 984)	1635 (1404 to 1869)	3.12 (2.65 to 3.70)	3.41 (2.92 to 3.90)	0.81 (0.58 to 1.03)
GBTC	Male	180 (153 to 212)	266 (223 to 314)	1.05 (0.89 to 1.23)	0.72 (0.60 to 0.85)	-1.24 (-1.63 to -0.85)
	Female	342 (285 to 405)	340 (283 to 404)	1.29 (1.08 to 1.53)	0.71 (0.59 to 0.85)	-1.78 (-2.16 to -1.40)
**East Asia**						
EC	Male	164,040 (121,928 to 203,022)	201,790 (173,465 to 234,676)	38.88 (29.69 to 48.58)	17.61 (15.23 to 20.52)	-2.96 (-3.27 to -2.65)
	Female	76,627 (39,024 to 102,349)	59,578 (45,412 to 72,248)	17.56 (9.25 to 23.50)	4.70 (3.60 to 5.69)	-4.98 (-5.39 to -4.56)
SC	Male	282,117 (217,113 to 370,446)	277,643 (193,451 to 371,807)	67.16 (51.19 to 89.94)	24.48 (16.83 to 32.34)	-3.41 (-3.64 to -3.18)
	Female	153,603 (123,588 to 198,438)	114,457 (91,311 to 148,388)	34.62 (27.77 to 44.93)	9.24 (7.41 to 11.88)	-4.65 (-4.97 to -4.33)
CRC	Male	76,531 (62,429 to 97,037)	170,923 (148,442 to 199,944)	18.89 (15.44 to 23.78)	15.52 (13.54 to 18.08)	-0.90 (-1.05 to -0.75)
	Female	62,318 (48,167 to 80,841)	102,925 (82,571 to 139,222)	14.06 (10.91 to 18.44)	8.33 (6.74 to 11.18)	-2.06 (-2.19 to -1.92)
LC	Male	64,862 (54,644 to 77,624)	109,238 (93,949 to 127,923)	13.59 (11.37 to 16.39)	9.76 (8.46 to 11.38)	-1.22 (-1.39 to -1.05)

	Female	29,218 (23,052 to 37,723)	40,109 (31,548 to 47,643)	6.32 (4.96 to 8.14)	3.26 (2.58 to 3.87)	-2.35 (-2.54 to -2.16)
PC	Male	26,083 (21,522 to 33,019)	73,308 (62,786 to 85,605)	6.12 (5.03 to 7.77)	6.42 (5.54 to 7.51)	-0.08 (-0.20 to 0.05)
	Female	18,094 (14,774 to 22,362)	47,327 (38,364 to 60,149)	4.06 (3.33 to 5.03)	3.75 (3.05 to 4.75)	-0.66 (-0.83 to -0.49)
GBTC	Male	9004 (6767 to 12,811)	18,136 (13,824 to 27,248)	2.32 (1.72 to 3.19)	1.63 (1.24 to 2.40)	-1.26 (-1.45 to -1.07)
	Female	10,306 (7146 to 13,992)	17,468 (12,717 to 24,455)	2.37 (1.63 to 3.25)	1.39 (1.01 to 1.94)	-1.97 (-2.18 to -1.76)
**High-income Asia Pacific**						
EC	Male	8771 (8289 to 9277)	14,578 (13,174 to 15,777)	10.18 (9.55 to 10.79)	6.49 (5.92 to 7.00)	-1.64 (-1.76 to -1.51)
	Female	1913 (1621 to 2205)	3275 (2492 to 3882)	1.69 (1.43 to 1.95)	1.04 (0.85 to 1.20)	-1.61 (-1.76 to -1.47)
SC	Male	46,724 (41,896 to 49,973)	45,099 (39,619 to 53,332)	56.70 (51.89 to 60.98)	19.61 (17.32 to 23.61)	-3.38 (-3.44 to -3.32)
	Female	28,052 (25,338 to 30,168)	29,436 (20,656 to 37,164)	25.17 (22.69 to 27.11)	8.21 (6.28 to 10.37)	-3.91 (-4.05 to -3.78)
CRC	Male	20,043 (19,057 to 20,916)	46,744 (42,182 to 49,806)	25.00 (23.55 to 26.14)	20.93 (19.00 to 22.31)	-0.76 (-0.81 to -0.70)
	Female	17,228 (15,273 to 18,543)	43,592 (32,330 to 50,891)	15.35 (13.52 to 16.55)	12.19 (9.84 to 13.80)	-0.89 (-0.95 to -0.83)
LC	Male	21,758 (19,141 to 24,682)	30,539 (27,180 to 34,025)	23.98 (21.21 to 27.10)	14.04 (12.47 to 15.89)	-2.29 (-2.73 to -1.86)
	Female	7500 (6442 to 8717)	14,720 (11,189 to 17,128)	6.57 (5.62 to 7.66)	4.15 (3.30 to 4.73)	-2.14 (-2.72 to -1.56)
PC	Male	10,009 (9535 to 10,489)	29,326 (26,946 to 31,019)	12.04 (11.38 to 12.62)	13.21 (12.20 to 13.93)	0.16 (0.05 to 0.26)
	Female	8112 (7299 to 8672)	30,484 (23,121 to 35,389)	7.09 (6.34 to 7.58)	9.02 (7.30 to 10.16)	0.71 (0.58 to 0.85)
GBTC	Male	6925 (6436 to 7463)	15,871 (14,012 to 17,810)	8.82 (8.21 to 9.48)	6.66 (5.93 to 7.55)	-1.12 (-1.18 to -1.06)
	Female	9150 (8065 to 10,063)	15,234 (10,696 to 18,585)	7.99 (7.02 to 8.82)	3.89 (2.96 to 4.63)	-2.43 (-2.49 to -2.37)
**South Asia**						
EC	Male	13,295 (9256 to 17,602)	65,011 (46,432 to 88,081)	4.44 (3.07 to 5.90)	8.79 (6.24 to 12.05)	2.09 (1.68 to 2.51)
	Female	11,387 (7846 to 16,976)	57,191 (39,177 to 80,319)	4.24 (2.96 to 6.37)	7.51 (5.24 to 10.52)	1.49 (0.97 to 2.03)
SC	Male	32,072 (19,721 to 48,481)	65,225 (37,929 to 94,011)	10.72 (6.49 to 16.12)	8.64 (4.99 to 12.53)	-0.74 (-0.89 to -0.58)
	Female	17,769 (11,570 to 29,272)	38,653 (24,408 to 57,795)	6.32 (4.10 to 10.50)	4.92 (3.13 to 7.35)	-0.84 (-0.96 to -0.72)
CRC	Male	12,272 (9247 to 17,072)	41,224 (30,540 to 55,712)	4.22 (3.11 to 5.93)	5.67 (4.20 to 7.67)	0.78 (0.65 to 0.92)
	Female	12,159 (8520 to 16,557)	41,886 (31,949 to 55,700)	4.37 (3.04 to 5.92)	5.39 (4.11 to 7.16)	0.43 (0.27 to 0.59)
LC	Male	8558 (5238 to 12,836)	30,099 (17,848 to 46,491)	2.68 (1.64 to 4.04)	3.80 (2.28 to 5.89)	0.82 (0.65 to 1.00)
	Female	3921 (2309 to 6350)	15,223 (8723 to 23,699)	1.41 (0.82 to 2.28)	1.94 (1.11 to 3.01)	0.83 (0.69 to 0.97)
PC	Male	4039 (2661 to 5533)	16,877 (11,774 to 23,037)	1.39 (0.91 to 1.91)	2.26 (1.56 to 3.07)	1.32 (1.17 to 1.47)
	Female	2682 (1786 to 3855)	12,690 (9021 to 16,971)	1.05 (0.70 to 1.51)	1.66 (1.18 to 2.23)	1.21 (1.01 to 1.40)
GBTC	Male	3980 (2512 to 5890)	14,954 (9543 to 22,059)	1.47 (0.94 to 2.15)	2.10 (1.34 to 3.09)	1.10 (0.97 to 1.23)
	Female	6446 (4468 to 9274)	26,251 (17,144 to 38,017)	2.52 (1.73 to 3.63)	3.44 (2.24 to 4.97)	0.87 (0.75 to 0.98)
**Southeast**						

**Asia**						
EC	Male	5002 (3568 to 6563)	15,501 (12,133 to 19,924)	4.15 (2.96 to 5.46)	4.48 (3.48 to 5.79)	0.08 (-0.03 to 0.19)
	Female	2482 (1685 to 3548)	4717 (3684 to 6610)	1.85 (1.26 to 2.64)	1.26 (0.98 to 1.76)	-1.44 (-1.53 to -1.34)
SC	Male	15,867 (10,410 to 21,607)	32,681 (23,930 to 44,671)	13.49 (8.86 to 18.59)	9.77 (7.16 to 13.36)	-1.10 (-1.19 to -1.01)
	Female	9567 (6744 to 13,112)	17,561 (13,184 to 23,751)	6.98 (4.93 to 9.57)	4.62 (3.48 to 6.24)	-1.40 (-1.50 to -1.31)
CRC	Male	12,514 (9495 to 17,805)	53,479 (40,959 to 67,324)	10.80 (8.11 to 15.33)	16.26 (12.58 to 20.48)	1.27 (1.18 to 1.35)
	Female	9403 (7283 to 12,211)	36,407 (28,538 to 46,695)	7.03 (5.47 to 9.14)	9.59 (7.57 to 12.30)	0.90 (0.83 to 0.96)
LC	Male	13,733 (10,060 to 18,915)	37,866 (29,184 to 51,241)	10.77 (7.91 to 14.97)	10.82 (8.32 to 14.55)	-0.29 (-0.38 to -0.20)
	Female	5000 (3437 to 7397)	12,280 (8596 to 17,975)	3.71 (2.56 to 5.39)	3.27 (2.31 to 4.76)	-0.62 (-0.71 to -0.54)
PC	Male	3315 (2344 to 4224)	14,641 (11,677 to 18,377)	2.79 (1.97 to 3.55)	4.33 (3.44 to 5.41)	1.26 (1.19 to 1.34)
	Female	2403 (1729 to 3191)	11,467 (8825 to 14,600)	1.84 (1.32 to 2.44)	3.02 (2.33 to 3.85)	1.44 (1.38 to 1.50)
GBTC	Male	2537 (1721 to 3632)	8019 (6110 to 12,949)	2.30 (1.54 to 3.31)	2.56 (1.95 to 4.17)	0.13 (-0.03 to 0.29)
	Female	2142 (1518 to 3082)	5637 (4515 to 9335)	1.68 (1.19 to 2.44)	1.52 (1.22 to 2.54)	-0.56 (-0.70 to -0.42)

EC: esophageal cancer; SC: stomach cancer; CRC: colon and rectum cancer; LC: liver cancer; PC: pancreatic cancer; GBTC: gallbladder and biliary tract cancer; ASDR: age-standardized death rate; EAPC: estimated annual percent change; UI: uncertainty interval; CI: confidence interval

Table S6. DALYs, age-standardized rates, and changes of gastrointestinal cancers by sex in five GBD subregions of Asia, 1990-2023

		1990 DALYs (95% UI)	2023 DALYs (95% UI)	1990 ASDALYR Per 100 000 (95% UI)	2023 ASDALYR Per 100 000 (95% UI)	1990 to 2023 EAPC (95% CI)
**Central Asia**						
EC	Male	97,486 (90,671 to 103,600)	62,228 (57,520 to 67,878)	486.80 (452.78 to 519.44)	148.02 (136.54 to 162.60)	-3.75 (-3.99 to -3.51)
	Female	70,527 (65,202 to 75,163)	38,230 (35,204 to 44,162)	257.89 (237.92 to 274.98)	74.39 (68.31 to 86.01)	-3.95 (-4.11 to -3.80)
SC	Male	226,887 (205,614 to 242,513)	196,466 (176,369 to 225,073)	1070.82 (973.16 to 1145.69)	452.25 (406.21 to 512.31)	-2.47 (-2.61 to -2.32)
	Female	135,139 (125,293 to 145,348)	90,221 (82,714 to 97,809)	485.77 (451.04 to 522.23)	175.47 (160.68 to 190.35)	-3.00 (-3.08 to -2.92)
CRC	Male	71,789 (66,532 to 76,657)	100,816 (93,087 to 107,765)	338.67 (314.45 to 360.53)	238.99 (221.10 to 255.33)	-0.76 (-0.96 to -0.56)

	Female	70,672 (65,226 to 76,388)	78,263 (71,140 to 86,790)	253.53 (233.84 to 273.93)	153.67 (139.32 to 170.62)	-1.32 (-1.42 to -1.22)
LC	Male	70,728 (60,082 to 83,791)	100,107 (89,209 to 115,115)	321.94 (275.02 to 377.87)	226.23 (201.27 to 259.32)	-1.31 (-1.43 to -1.19)
	Female	43,402 (35,955 to 51,519)	60,061 (51,953 to 69,195)	150.46 (124.98 to 177.08)	117.87 (101.55 to 136.26)	-0.77 (-1.05 to -0.48)
PC	Male	24,912 (21,096 to 29,495)	59,086 (51,568 to 67,032)	117.09 (98.80 to 138.59)	136.83 (119.49 to 155.25)	0.91 (0.69 to 1.13)
	Female	21,231 (18,130 to 25,221)	40,752 (35,043 to 46,456)	77.15 (65.77 to 91.69)	79.79 (68.68 to 91.09)	0.53 (0.33 to 0.72)
GBTC	Male	5010 (4249 to 5878)	7045 (5908 to 8338)	25.36 (21.52 to 29.76)	16.91 (14.15 to 19.99)	-1.40 (-1.76 to -1.04)
	Female	8619 (7232 to 10,193)	8420 (7026 to 9991)	31.53 (26.44 to 37.32)	16.50 (13.74 to 19.57)	-2.04 (-2.40 to -1.68)
**East Asia**						
EC	Male	4,601,079 (3,394,559 to 5,665,945)	4,803,223 (4,172,487 to 5,541,108)	982.15 (726.62 to 1215.24)	407.59 (356.02 to 467.33)	-3.27 (-3.59 to -2.94)
	Female	1,881,715 (921,715 to 2,495,620)	1,117,275 (873,413 to 1,344,704)	406.83 (202.06 to 540.25)	88.16 (69.42 to 105.50)	-5.68 (-6.12 to -5.24)
SC	Male	8,120,674 (6,213,313 to 10,718,484)	6,732,675 (4,896,774 to 9,047,421)	1713.72 (1316.43 to 2254.55)	582.16 (418.22 to 782.55)	-3.68 (-3.92 to -3.43)
	Female	4,235,404 (3,492,357 to 5,396,957)	2,433,756 (1,999,763 to 3,067,937)	878.33 (722.02 to 1126.52)	203.07 (169.36 to 252.96)	-5.10 (-5.42 to -4.79)
CRC	Male	2,391,385 (1,945,762 to 2,980,599)	4,344,230 (3,769,805 to 5,037,188)	492.37 (404.18 to 619.95)	388.35 (336.07 to 446.53)	-1.08 (-1.22 to -0.93)
	Female	1,837,044 (1,393,923 to 2,318,417)	2,305,824 (1,910,057 to 3,108,153)	373.31 (284.84 to 474.96)	193.50 (160.56 to 257.09)	-2.51 (-2.65 to -2.36)
LC	Male	2,270,385 (1,920,436 to 2,705,297)	3,277,875 (2,816,907 to 3,900,889)	430.20 (363.30 to 512.23)	300.04 (258.22 to 356.30)	-1.37 (-1.53 to -1.21)
	Female	903,056 (729,481 to 1,136,928)	936,799 (765,835 to 1,112,364)	181.56 (145.66 to 230.45)	80.63 (66.63 to 95.29)	-2.86 (-3.04 to -2.67)
PC	Male	776,602 (640,923 to 969,884)	1,863,229 (1,603,263 to 2,164,381)	160.70 (132.48 to 202.31)	161.43 (139.14 to 185.96)	-0.23 (-0.35 to -0.11)

	Female	491,679 (406,182 to 596,751)	1,037,014 (869,615 to 1,280,957)	102.94 (84.84 to 125.91)	83.90 (70.78 to 102.61)	-1.06 (-1.22 to -0.91)
GBTC	Male	248,112 (189,430 to 356,313)	424,707 (327,471 to 649,413)	54.66 (41.41 to 77.62)	37.10 (28.35 to 56.09)	-1.39 (-1.58 to -1.20)
	Female	265,219 (185,509 to 354,791)	365,803 (274,200 to 527,257)	56.60 (39.39 to 75.89)	29.49 (22.14 to 42.29)	-2.33 (-2.53 to -2.13)
**High-income Asia Pacific**						
EC	Male	228,079 (215,021 to 241,812)	273,695 (252,929 to 293,635)	246.01 (231.97 to 260.78)	133.78 (123.81 to 144.28)	-2.18 (-2.34 to -2.02)
	Female	39,468 (33,689 to 45,234)	54,449 (44,370 to 63,257)	34.82 (29.75 to 39.88)	22.58 (19.28 to 25.90)	-1.43 (-1.55 to -1.31)
SC	Male	1,202,666 (1,043,192 to 1,286,300)	786,244 (697,604 to 968,410)	1329.24 (1164.22 to 1421.31)	382.99 (341.68 to 483.26)	-3.95 (-4.01 to -3.88)
	Female	688,160 (637,030 to 736,036)	434,127 (331,183 to 550,064)	630.15 (582.16 to 672.88)	171.59 (141.04 to 215.02)	-4.44 (-4.58 to -4.30)
CRC	Male	518,030 (492,043 to 543,843)	906,884 (832,433 to 971,889)	580.29 (550.74 to 607.89)	457.18 (424.17 to 489.93)	-0.96 (-1.02 to -0.91)
	Female	400,167 (369,020 to 426,550)	679,194 (546,069 to 767,897)	358.85 (329.81 to 383.65)	262.18 (228.36 to 287.01)	-1.17 (-1.25 to -1.10)
LC	Male	632,552 (550,432 to 725,370)	587,755 (521,901 to 679,520)	660.47 (574.68 to 756.06)	306.13 (269.75 to 360.34)	-3.00 (-3.44 to -2.56)
	Female	173,873 (149,327 to 204,914)	215,490 (170,249 to 246,884)	155.41 (132.94 to 183.60)	79.92 (67.50 to 92.01)	-2.82 (-3.38 to -2.25)
PC	Male	249,278 (237,155 to 262,223)	549,522 (512,144 to 577,849)	275.16 (262.39 to 288.88)	275.25 (257.40 to 289.91)	-0.15 (-0.24 to -0.05)
	Female	171,340 (156,707 to 184,375)	465,077 (375,611 to 524,387)	150.26 (137.05 to 162.17)	174.59 (149.22 to 191.28)	0.43 (0.32 to 0.55)
GBTC	Male	156,064 (142,261 to 170,679)	254,703 (228,251 to 289,995)	180.01 (165.18 to 195.39)	116.88 (105.05 to 135.09)	-1.60 (-1.66 to -1.53)
	Female	184,777 (164,252 to 202,608)	205,268 (155,094 to 244,278)	161.41 (143.44 to 177.22)	67.21 (54.51 to 79.08)	-2.93 (-2.99 to -2.86)
**South Asia**						

EC	Male	396,875 (278,743 to 527,560)	1,813,132 (1,308,527 to 2,465,450)	117.60 (81.93 to 155.50)	221.19 (159.31 to 300.57)	1.91 (1.51 to 2.31)
	Female	341,103 (232,537 to 504,463)	1,544,235 (1,038,756 to 2,197,988)	110.94 (75.89 to 163.98)	186.94 (126.51 to 265.48)	1.33 (0.80 to 1.87)
SC	Male	968,012 (600,444 to 1,463,349)	1,845,871 (1,088,544 to 2,685,643)	284.77 (175.22 to 429.81)	223.46 (130.70 to 323.88)	-0.85 (-1.02 to -0.69)
	Female	580,391 (372,036 to 941,814)	1,129,436 (690,802 to 1,717,960)	176.62 (114.32 to 289.12)	132.87 (81.91 to 201.32)	-0.99 (-1.12 to -0.85)
CRC	Male	370,974 (282,500 to 516,756)	1,166,597 (867,158 to 1,559,292)	109.66 (82.91 to 152.30)	142.54 (105.75 to 190.58)	0.66 (0.51 to 0.81)
	Female	392,805 (274,775 to 544,889)	1,224,478 (926,510 to 1,639,263)	121.72 (85.50 to 166.26)	145.27 (110.07 to 195.26)	0.29 (0.09 to 0.48)
LC	Male	279,778 (170,518 to 419,956)	917,076 (534,963 to 1,401,015)	77.02 (47.09 to 115.43)	107.78 (63.06 to 164.10)	0.77 (0.59 to 0.95)
	Female	127,308 (74,595 to 209,071)	431,680 (249,898 to 675,177)	38.17 (22.45 to 61.93)	51.42 (29.69 to 80.27)	0.75 (0.63 to 0.87)
PC	Male	116,436 (77,817 to 157,644)	461,021 (323,874 to 625,693)	35.37 (23.38 to 48.38)	56.54 (39.71 to 77.23)	1.25 (1.08 to 1.42)
	Female	72,804 (48,523 to 103,509)	321,311 (229,401 to 431,166)	25.15 (16.76 to 36.11)	39.40 (28.06 to 52.82)	1.16 (0.93 to 1.39)
GBTC	Male	108,967 (68,560 to 161,178)	384,038 (243,125 to 556,903)	34.61 (21.90 to 51.12)	48.52 (30.75 to 70.44)	1.02 (0.88 to 1.16)
	Female	179,086 (123,532 to 259,294)	677,615 (436,912 to 979,457)	61.29 (42.62 to 88.41)	82.94 (53.67 to 119.39)	0.81 (0.70 to 0.92)
**Southeast Asia**						
EC	Male	149,964 (106,294 to 196,344)	462,738 (362,650 to 593,654)	111.19 (79.15 to 145.55)	124.01 (97.36 to 159.50)	0.19 (0.07 to 0.30)
	Female	71,995 (48,606 to 105,565)	126,462 (96,752 to 178,926)	48.06 (32.62 to 70.02)	32.10 (24.66 to 45.16)	-1.49 (-1.59 to -1.38)
SC	Male	476,619 (311,977 to 654,101)	967,204 (699,397 to 1,303,918)	351.02 (230.73 to 477.41)	264.17 (191.56 to 357.19)	-0.96 (-1.05 to -0.87)
	Female	293,536 (205,559 to 408,451)	505,674 (374,107 to 681,207)	188.67 (132.70 to 259.99)	127.78 (94.71 to 171.92)	-1.33 (-1.45 to -1.21)
CRC	Male	381,551 (286,192 to 548,027)	1,574,767 (1,194,585 to 1,991,004)	279.37 (211.67 to 398.44)	434.84 (332.21 to 548.44)	1.38 (1.30 to 1.46)

	Female	280,574 (217,615 to 371,614)	1,037,199 (801,739 to 1,341,476)	183.86 (142.45 to 241.03)	261.78 (202.91 to 338.33)	1.05 (1.01 to 1.10)
LC	Male	456,873 (333,285 to 629,692)	1,194,762 (911,088 to 1,639,864)	311.32 (227.34 to 428.94)	318.48 (243.44 to 435.72)	-0.26 (-0.36 to -0.16)
	Female	151,263 (102,403 to 228,343)	336,097 (232,844 to 503,285)	97.01 (66.11 to 145.06)	86.12 (60.03 to 128.56)	-0.62 (-0.72 to -0.52)
PC	Male	99,303 (70,104 to 127,782)	430,039 (338,934 to 544,647)	73.27 (51.79 to 93.58)	116.95 (92.29 to 147.88)	1.35 (1.28 to 1.43)
	Female	65,231 (46,825 to 87,544)	302,289 (233,107 to 385,281)	45.09 (32.48 to 60.24)	76.22 (58.78 to 96.98)	1.55 (1.50 to 1.60)
GBTC	Male	68,792 (46,552 to 97,618)	209,766 (160,083 to 332,530)	54.86 (37.10 to 78.36)	60.23 (46.08 to 96.22)	0.06 (-0.11 to 0.24)
	Female	55,408 (39,382 to 78,308)	136,508 (109,152 to 228,664)	39.38 (28.01 to 56.12)	35.04 (28.11 to 58.06)	-0.63 (-0.78 to -0.48)

EC: esophageal cancer; SC: stomach cancer; CRC: colon and rectum cancer; LC: liver cancer; PC: pancreatic cancer; GBTC: gallbladder and biliary tract cancer; DALYs: disability-adjusted life years; ASDALYR: age-standardized disability-adjusted life years rate; EAPC: estimated annual percent change; UI: uncertainty interval; CI: confidence interval

Table S7. Age-specific incidence rates and male-to-female ratios of esophageal cancer by region in Asia, 2023

Location	Age Group	Both	Male	Female	M/F
Central Asia	0-14	0.00 (0.00 to 0.00)	0.00 (0.00 to 0.00)	0.00 (0.00 to 0.00)	NA
	15-49	0.82 (0.75 to 0.90)	0.87 (0.78 to 0.96)	0.77 (0.66 to 0.90)	1.12
	50-69	13.24 (12.34 to 14.35)	18.31 (16.75 to 20.04)	8.83 (8.01 to 10.38)	2.07
	≥70	30.54 (27.69 to 33.34)	45.24 (40.31 to 51.09)	21.05 (18.43 to 24.42)	2.15
East Asia	0-14	0.00 (0.00 to 0.00)	0.00 (0.00 to 0.00)	0.00 (0.00 to 0.00)	NA
	15-49	1.99 (1.64 to 2.42)	3.38 (2.73 to 4.25)	0.49 (0.37 to 0.62)	6.94
	50-69	31.67 (27.02 to 37.47)	53.86 (45.28 to 64.11)	9.53 (7.21 to 12.03)	5.65
	≥70	92.90 (73.97 to 109.40)	139.80 (112.38 to 170.56)	53.10 (37.15 to 66.53)	2.63
High-income Asia Pacific	0-14	0.00 (0.00 to 0.00)	0.00 (0.00 to 0.00)	0.00 (0.00 to 0.00)	NA
	15-49	0.71 (0.62 to 0.82)	0.95 (0.83 to 1.15)	0.44 (0.37 to 0.53)	2.15
	50-69	14.95 (13.89 to 16.20)	25.61 (23.51 to 27.89)	4.38 (3.83 to 5.02)	5.85
	≥70	51.54 (44.81 to 56.12)	99.34 (88.54 to 107.65)	16.80 (12.35 to 20.24)	5.91
South Asia	0-14	0.00 (0.00 to 0.00)	0.00 (0.00 to 0.00)	0.00 (0.00 to 0.00)	NA
	15-49	2.26 (1.69 to 2.98)	2.34 (1.62 to 3.35)	2.17 (1.39 to 3.16)	1.08

	50-69	22.14 (16.99 to 29.14)	24.56 (17.01 to 33.25)	19.71 (13.17 to 28.37)	1.25
	≥70	53.38 (41.15 to 68.47)	55.58 (38.63 to 76.03)	51.38 (35.22 to 72.70)	1.08
Southeast Asia	0-14	0.00 (0.00 to 0.00)	0.00 (0.00 to 0.00)	0.00 (0.00 to 0.00)	NA
	15-49	0.92 (0.72 to 1.19)	1.36 (1.02 to 1.84)	0.48 (0.34 to 0.70)	2.85
	50-69	9.93 (7.99 to 12.32)	16.72 (13.08 to 21.45)	3.57 (2.71 to 5.09)	4.69
	≥70	15.21 (12.26 to 19.04)	23.34 (17.45 to 30.32)	9.15 (7.12 to 12.50)	2.55

Table S8. Age-specific death rates and male-to-female ratios of esophageal cancer by region in Asia, 2023

Location	Age Group	Both	Male	Female	M/F
Central Asia	0-14	0.00 (0.00 to 0.00)	0.00 (0.00 to 0.00)	0.00 (0.00 to 0.00)	NA
	15-49	0.64 (0.59 to 0.70)	0.71 (0.64 to 0.79)	0.58 (0.50 to 0.67)	1.23
	50-69	12.47 (11.63 to 13.54)	17.38 (15.92 to 19.05)	8.20 (7.46 to 9.69)	2.12
	≥70	34.86 (31.64 to 38.10)	51.42 (45.96 to 58.06)	24.18 (21.15 to 28.02)	2.13
East Asia	0-14	0.00 (0.00 to 0.00)	0.00 (0.00 to 0.00)	0.00 (0.00 to 0.00)	NA
	15-49	1.36 (1.10 to 1.67)	2.37 (1.90 to 3.01)	0.25 (0.19 to 0.31)	9.65
	50-69	27.20 (23.33 to 32.07)	46.89 (39.13 to 55.91)	7.55 (5.79 to 9.51)	6.21
	≥70	97.54 (78.42 to 114.86)	147.27 (117.50 to 180.83)	55.36 (39.95 to 69.20)	2.66
High-income Asia Pacific	0-14	0.00 (0.00 to 0.00)	0.00 (0.00 to 0.00)	0.00 (0.00 to 0.00)	NA
	15-49	0.31 (0.28 to 0.36)	0.44 (0.38 to 0.52)	0.18 (0.15 to 0.20)	2.50
	50-69	8.42 (7.87 to 9.06)	14.51 (13.51 to 15.62)	2.37 (2.07 to 2.70)	6.13
	≥70	37.25 (31.93 to 40.66)	71.21 (63.08 to 76.98)	12.57 (9.10 to 15.15)	5.66
South Asia	0-14	0.00 (0.00 to 0.00)	0.00 (0.00 to 0.00)	0.00 (0.00 to 0.00)	NA
	15-49	1.77 (1.33 to 2.35)	1.88 (1.30 to 2.69)	1.66 (1.07 to 2.42)	1.13
	50-69	20.65 (15.85 to 27.20)	23.09 (15.96 to 31.29)	18.19 (12.17 to 26.12)	1.27
	≥70	61.62 (47.38 to 78.91)	64.00 (44.44 to 87.52)	59.46 (40.56 to 83.90)	1.08
Southeast Asia	0-14	0.00 (0.00 to 0.00)	0.00 (0.00 to 0.00)	0.00 (0.00 to 0.00)	NA
	15-49	0.71 (0.56 to 0.92)	1.07 (0.81 to 1.44)	0.33 (0.23 to 0.48)	3.27
	50-69	8.97 (7.20 to 11.13)	15.21 (11.88 to 19.61)	3.14 (2.38 to 4.51)	4.84
	≥70	16.67 (13.36 to 20.74)	25.63 (19.08 to 33.15)	9.99 (7.77 to 13.41)	2.57

Table S9. Age-specific DALY rates and male-to-female ratios of esophageal cancer by region in Asia, 2023

Location	Age Group	Both	Male	Female	M/F
Central Asia	0-14	0.00 (0.00 to 0.00)	0.00 (0.00 to 0.00)	0.00 (0.00 to 0.00)	NA
	15-49	30.94 (28.06 to 33.58)	33.67 (30.24 to 37.37)	28.16 (24.42 to 32.81)	1.20
	50-69	372.10 (346.53 to 404.13)	517.24 (473.13 to 565.63)	245.77 (222.31 to 289.33)	2.10
	≥70	570.08 (518.96 to 621.61)	858.93 (764.42 to 971.53)	383.60 (334.64 to 445.78)	2.24
East Asia	0-14	0.00 (0.00 to 0.00)	0.00 (0.00 to 0.00)	0.00 (0.00 to 0.00)	NA
	15-49	62.75 (51.55 to 76.66)	109.68 (88.30 to 137.87)	11.59 (9.11 to 14.64)	9.46
	50-69	808.12 (696.79 to 945.80)	1401.51 (1173.31 to 1658.63)	215.94 (167.44 to 270.49)	6.49
	≥70	1516.98 (1204.90 to 1788.89)	2344.88 (1865.32 to 2859.45)	814.57 (590.48 to 1018.47)	2.88
High-income Asia Pacific	0-14	0.00 (0.00 to 0.00)	0.00 (0.00 to 0.00)	0.00 (0.00 to 0.00)	NA
	15-49	14.32 (12.83 to 16.53)	20.17 (17.59 to 23.74)	8.13 (6.99 to 9.36)	2.48
	50-69	245.18 (229.67 to 263.17)	420.72 (390.62 to 452.14)	70.92 (62.12 to 80.88)	5.93
	≥70	538.12 (471.51 to 583.15)	1056.95 (951.89 to 1140.64)	161.00 (120.08 to 191.00)	6.56
South Asia	0-14	0.00 (0.00 to 0.00)	0.00 (0.00 to 0.00)	0.00 (0.00 to 0.00)	NA
	15-49	86.04 (63.87 to 113.61)	90.88 (62.83 to 129.78)	81.00 (52.16 to 116.82)	1.12
	50-69	631.23 (483.76 to 825.20)	701.71 (486.11 to 946.99)	560.40 (375.97 to 805.30)	1.25
	≥70	973.55 (744.50 to 1255.44)	1027.14 (711.71 to 1402.82)	924.80 (634.01 to 1305.60)	1.11
Southeast Asia	0-14	0.00 (0.00 to 0.00)	0.00 (0.00 to 0.00)	0.00 (0.00 to 0.00)	NA
	15-49	33.55 (26.43 to 43.53)	50.43 (37.80 to 67.72)	16.19 (11.24 to 23.24)	3.11
	50-69	279.72 (224.75 to 348.70)	475.45 (371.53 to 612.79)	96.70 (72.81 to 139.84)	4.92
	≥70	273.48 (216.18 to 342.88)	432.79 (320.30 to 565.03)	154.86 (122.23 to 213.32)	2.79

DALY: Disability-Adjusted Life Year

Table S10. Age-specific incidence rates and male-to-female ratios of stomach cancer by region in Asia, 2023

Location	Age Group	Both	Male	Female	M/F
Central Asia	0-14	0.00 (0.00 to 0.00)	0.00 (0.00 to 0.00)	0.00 (0.00 to 0.00)	NA
	15-49	3.18 (2.92 to 3.51)	3.96 (3.49 to 4.61)	2.39 (2.12 to 2.72)	1.66
	50-69	37.17 (34.18 to 42.48)	57.69 (51.93 to 66.72)	19.32 (17.39 to 21.56)	2.99
	≥70	77.06 (69.56 to 85.38)	122.71 (109.67 to 141.55)	47.58 (41.81 to 53.25)	2.58
East Asia	0-14	0.00 (0.00 to 0.00)	0.00 (0.00 to 0.00)	0.00 (0.00 to 0.00)	NA
	15-49	6.93 (5.30 to 9.47)	9.90 (6.83 to 14.61)	3.69 (2.80 to 4.68)	2.68
	50-69	67.38 (50.26 to 90.10)	106.23 (75.37 to 151.56)	28.61 (21.85 to 36.77)	3.71

	≥70	187.47 (137.65 to 243.72)	275.26 (178.52 to 393.66)	112.99 (82.84 to 155.64)	2.44
High-income Asia Pacific	0-14	0.00 (0.00 to 0.00)	0.00 (0.00 to 0.00)	0.00 (0.00 to 0.00)	NA
	15-49	6.92 (6.05 to 9.07)	6.92 (5.92 to 9.84)	6.92 (5.76 to 8.45)	1.00
	50-69	54.84 (49.16 to 71.78)	80.35 (70.62 to 108.91)	29.51 (24.61 to 37.14)	2.72
	≥70	278.82 (221.79 to 325.95)	404.85 (356.36 to 464.47)	187.21 (129.76 to 235.48)	2.16
South Asia	0-14	0.00 (0.00 to 0.00)	0.00 (0.00 to 0.00)	0.00 (0.00 to 0.00)	NA
	15-49	2.09 (1.50 to 2.83)	2.21 (1.27 to 3.35)	1.96 (1.20 to 3.14)	1.13
	50-69	20.63 (14.36 to 27.50)	27.64 (16.69 to 40.97)	13.58 (8.43 to 21.58)	2.03
	≥70	41.35 (29.38 to 55.24)	53.15 (30.88 to 78.92)	30.62 (19.27 to 46.51)	1.74
Southeast Asia	0-14	0.00 (0.00 to 0.00)	0.00 (0.00 to 0.00)	0.00 (0.00 to 0.00)	NA
	15-49	3.10 (2.32 to 3.95)	3.81 (2.60 to 5.31)	2.38 (1.67 to 3.45)	1.60
	50-69	23.06 (18.07 to 30.16)	33.40 (24.70 to 45.07)	13.39 (10.10 to 18.55)	2.49
	≥70	43.68 (34.28 to 58.31)	62.17 (44.85 to 87.27)	29.92 (22.49 to 40.05)	2.08

Table S11. Age-specific death rates and male-to-female ratios of stomach cancer by region in Asia, 2023

Location	Age Group	Both	Male	Female	M/F
Central Asia	0-14	0.00 (0.00 to 0.00)	0.00 (0.00 to 0.00)	0.00 (0.00 to 0.00)	NA
	15-49	2.52 (2.32 to 2.77)	3.14 (2.77 to 3.65)	1.89 (1.68 to 2.13)	1.66
	50-69	33.75 (31.02 to 38.04)	52.42 (47.17 to 60.64)	17.50 (15.83 to 19.35)	3.00
	≥70	84.43 (76.55 to 92.30)	133.23 (119.01 to 152.94)	52.92 (46.81 to 59.08)	2.52
East Asia	0-14	0.00 (0.00 to 0.00)	0.00 (0.00 to 0.00)	0.00 (0.00 to 0.00)	NA
	15-49	3.34 (2.64 to 4.46)	4.72 (3.47 to 6.85)	1.84 (1.50 to 2.28)	2.56
	50-69	39.69 (31.27 to 52.59)	61.57 (46.30 to 86.86)	17.86 (14.09 to 22.47)	3.45
	≥70	144.15 (108.60 to 176.72)	204.91 (132.39 to 266.68)	92.61 (69.69 to 121.32)	2.21
High-income Asia Pacific	0-14	0.00 (0.00 to 0.00)	0.00 (0.00 to 0.00)	0.00 (0.00 to 0.00)	NA
	15-49	2.18 (1.95 to 2.83)	2.16 (1.84 to 3.06)	2.21 (1.90 to 2.70)	0.98
	50-69	22.99 (20.61 to 30.06)	33.75 (29.68 to 45.63)	12.31 (10.23 to 15.50)	2.74
	≥70	170.96 (133.77 to 199.24)	237.00 (205.19 to 269.73)	122.96 (82.84 to 155.32)	1.93
South Asia	0-14	0.00 (0.00 to 0.00)	0.00 (0.00 to 0.00)	0.00 (0.00 to 0.00)	NA
	15-49	1.65 (1.19 to 2.22)	1.75 (1.01 to 2.66)	1.54 (0.94 to 2.45)	1.13
	50-69	18.79 (13.20 to 25.18)	25.14 (15.20 to 37.48)	12.41 (7.75 to 19.54)	2.03
	≥70	46.18 (32.81 to 62.37)	58.90 (33.90 to 87.55)	34.60 (21.78 to 52.36)	1.70

Southeast Asia	0-14	0.00 (0.00 to 0.00)	0.00 (0.00 to 0.00)	0.00 (0.00 to 0.00)	NA
	15-49	2.27 (1.69 to 2.95)	2.73 (1.86 to 3.86)	1.80 (1.26 to 2.60)	1.52
	50-69	19.56 (15.45 to 25.28)	28.16 (21.06 to 37.95)	11.52 (8.52 to 16.01)	2.44
	≥70	46.24 (36.39 to 61.62)	64.88 (46.95 to 90.69)	32.37 (23.95 to 43.47)	2.00

Table S12. Age-specific DALY rates and male-to-female ratios of stomach cancer by region in Asia, 2023

Location	Age Group	Both	Male	Female	M/F
Central Asia	0-14	0.00 (0.00 to 0.00)	0.00 (0.00 to 0.00)	0.00 (0.00 to 0.00)	NA
	15-49	123.86 (113.86 to 135.59)	152.70 (135.08 to 177.87)	94.60 (84.11 to 106.74)	1.61
	50-69	1011.34 (927.97 to 1136.69)	1574.39 (1410.62 to 1824.38)	521.29 (470.31 to 574.78)	3.02
	≥70	1394.87 (1271.17 to 1531.69)	2258.21 (2027.88 to 2591.04)	837.53 (743.62 to 935.08)	2.70
East Asia	0-14	0.00 (0.00 to 0.00)	0.00 (0.00 to 0.00)	0.00 (0.00 to 0.00)	NA
	15-49	160.87 (127.53 to 212.27)	225.40 (164.60 to 323.42)	90.54 (73.57 to 110.19)	2.49
	50-69	1190.23 (942.62 to 1577.08)	1851.09 (1384.74 to 2573.26)	530.73 (422.35 to 667.87)	3.49
	≥70	2240.25 (1698.14 to 2784.41)	3277.68 (2159.27 to 4384.99)	1360.08 (1018.56 to 1814.32)	2.41
High-income Asia Pacific	0-14	0.00 (0.00 to 0.00)	0.00 (0.00 to 0.00)	0.00 (0.00 to 0.00)	NA
	15-49	106.40 (95.18 to 137.89)	104.29 (88.87 to 147.80)	108.63 (93.25 to 133.00)	0.96
	50-69	676.33 (603.73 to 884.43)	987.02 (864.39 to 1350.14)	367.93 (307.71 to 460.99)	2.68
	≥70	2225.17 (1805.01 to 2602.02)	3291.21 (2893.83 to 3776.18)	1450.32 (1018.32 to 1839.38)	2.27
South Asia	0-14	0.00 (0.00 to 0.00)	0.00 (0.00 to 0.00)	0.00 (0.00 to 0.00)	NA
	15-49	81.76 (58.78 to 111.00)	84.68 (48.87 to 127.73)	78.72 (47.80 to 124.25)	1.08
	50-69	571.64 (402.47 to 769.51)	765.81 (462.31 to 1142.22)	376.50 (234.80 to 598.55)	2.03
	≥70	746.61 (528.60 to 1001.57)	966.68 (560.68 to 1417.10)	546.39 (346.39 to 824.46)	1.77
Southeast Asia	0-14	0.00 (0.00 to 0.00)	0.00 (0.00 to 0.00)	0.00 (0.00 to 0.00)	NA
	15-49	111.97 (83.60 to 145.09)	133.44 (90.69 to 188.11)	89.87 (63.00 to 130.56)	1.48
	50-69	603.09 (474.68 to 785.38)	870.56 (644.30 to 1165.89)	353.00 (260.91 to 490.90)	2.47
	≥70	744.62 (586.64 to 989.77)	1074.80 (774.30 to 1485.97)	498.77 (372.85 to 662.77)	2.15

DALY: Disability-Adjusted Life Year

Table S13. Age-specific incidence rates and male-to-female ratios of colorectal cancer by region in Asia, 2023

Location	Age Group	Both	Male	Female	M/F

Central Asia	0-14	0.00 (0.00 to 0.00)	0.00 (0.00 to 0.00)	0.00 (0.00 to 0.00)	NA
	15-49	2.92 (2.62 to 3.22)	3.12 (2.71 to 3.49)	2.72 (2.33 to 3.12)	1.15
	50-69	30.76 (27.72 to 33.94)	38.36 (34.32 to 42.82)	24.15 (21.22 to 27.29)	1.59
	≥70	73.36 (66.09 to 80.59)	94.71 (86.37 to 103.05)	59.58 (51.73 to 67.70)	1.59
East Asia	0-14	0.00 (0.00 to 0.00)	0.00 (0.00 to 0.00)	0.00 (0.00 to 0.00)	NA
	15-49	7.74 (6.17 to 9.64)	10.90 (8.22 to 13.96)	4.29 (3.21 to 5.69)	2.54
	50-69	72.80 (58.40 to 89.68)	99.61 (80.18 to 121.92)	46.05 (34.51 to 61.37)	2.16
	≥70	208.28 (165.81 to 273.52)	264.48 (206.58 to 334.41)	160.59 (119.09 to 233.73)	1.65
High-income Asia Pacific	0-14	0.00 (0.00 to 0.00)	0.00 (0.00 to 0.00)	0.00 (0.00 to 0.00)	NA
	15-49	12.10 (10.48 to 14.06)	13.52 (11.53 to 15.93)	10.61 (8.71 to 12.31)	1.28
	50-69	124.87 (112.27 to 136.23)	166.50 (151.34 to 182.23)	83.56 (71.48 to 92.74)	1.99
	≥70	458.51 (379.21 to 515.04)	589.61 (516.55 to 645.33)	363.23 (268.46 to 428.34)	1.62
South Asia	0-14	0.00 (0.00 to 0.00)	0.00 (0.00 to 0.00)	0.00 (0.00 to 0.00)	NA
	15-49	2.20 (1.64 to 2.79)	1.96 (1.36 to 2.64)	2.44 (1.67 to 3.40)	0.80
	50-69	20.22 (15.84 to 24.96)	20.32 (14.89 to 27.40)	20.11 (14.45 to 27.18)	1.01
	≥70	40.90 (32.60 to 51.44)	43.20 (30.98 to 59.39)	38.81 (28.88 to 52.09)	1.11
Southeast Asia	0-14	0.00 (0.00 to 0.00)	0.00 (0.00 to 0.00)	0.00 (0.00 to 0.00)	NA
	15-49	6.92 (5.30 to 8.94)	7.90 (5.60 to 10.28)	5.90 (4.29 to 7.89)	1.34
	50-69	58.32 (45.75 to 70.15)	75.28 (56.58 to 94.26)	42.46 (32.27 to 53.58)	1.77
	≥70	104.90 (84.66 to 126.26)	136.27 (105.14 to 171.37)	81.54 (63.31 to 102.72)	1.67

Table S14. Age-specific death rates and male-to-female ratios of colorectal cancer by region in Asia, 2023

Location	Age Group	Both	Male	Female	M/F
Central Asia	0-14	0.00 (0.00 to 0.00)	0.00 (0.00 to 0.00)	0.00 (0.00 to 0.00)	NA
	15-49	1.56 (1.44 to 1.69)	1.70 (1.53 to 1.87)	1.41 (1.26 to 1.60)	1.20
	50-69	19.59 (18.29 to 20.78)	24.72 (22.77 to 26.74)	15.12 (13.61 to 16.63)	1.64
	≥70	63.59 (58.97 to 68.89)	81.14 (74.74 to 87.21)	52.26 (47.32 to 58.62)	1.55
East Asia	0-14	0.00 (0.00 to 0.00)	0.00 (0.00 to 0.00)	0.00 (0.00 to 0.00)	NA
	15-49	3.10 (2.61 to 3.71)	4.25 (3.36 to 5.28)	1.84 (1.46 to 2.48)	2.31
	50-69	26.08 (22.17 to 31.13)	35.51 (29.26 to 42.21)	16.67 (13.26 to 22.46)	2.13
	≥70	102.24 (84.59 to 124.71)	126.98 (105.73 to 154.24)	81.26 (61.85 to 110.09)	1.56
High-income Asia Pacific	0-14	0.00 (0.00 to 0.00)	0.00 (0.00 to 0.00)	0.00 (0.00 to 0.00)	NA

	15-49	2.67 (2.48 to 2.87)	2.88 (2.57 to 3.20)	2.45 (2.23 to 2.67)	1.18
	50-69	31.41 (29.32 to 33.49)	41.66 (38.87 to 44.68)	21.23 (19.18 to 23.28)	1.96
	≥70	202.11 (161.55 to 227.35)	232.77 (206.07 to 250.09)	179.82 (127.92 to 212.56)	1.29
South Asia	0-14	0.00 (0.00 to 0.00)	0.00 (0.00 to 0.00)	0.00 (0.00 to 0.00)	NA
	15-49	1.35 (1.03 to 1.70)	1.20 (0.86 to 1.59)	1.51 (1.04 to 2.08)	0.80
	50-69	14.43 (11.29 to 17.85)	14.58 (10.73 to 19.95)	14.28 (10.35 to 19.44)	1.02
	≥70	38.61 (30.95 to 48.45)	40.68 (29.67 to 55.02)	36.73 (26.74 to 49.30)	1.11
Southeast Asia	0-14	0.00 (0.00 to 0.00)	0.00 (0.00 to 0.00)	0.00 (0.00 to 0.00)	NA
	15-49	3.72 (2.93 to 4.72)	4.24 (2.95 to 5.72)	3.19 (2.34 to 4.25)	1.33
	50-69	34.63 (28.13 to 41.74)	45.04 (33.78 to 57.28)	24.90 (19.29 to 31.91)	1.81
	≥70	87.91 (71.85 to 105.94)	113.73 (89.69 to 140.61)	68.68 (54.23 to 86.97)	1.66

Table S15. Age-specific DALY rates and male-to-female ratios of colorectal cancer by region in Asia, 2023

Location	Age Group	Both	Male	Female	M/F
Central Asia	0-14	0.00 (0.00 to 0.00)	0.00 (0.00 to 0.00)	0.00 (0.00 to 0.00)	NA
	15-49	78.60 (72.98 to 84.98)	85.74 (77.58 to 94.29)	71.36 (63.79 to 80.61)	1.20
	50-69	591.39 (552.61 to 628.79)	746.22 (686.43 to 808.72)	456.64 (410.69 to 502.66)	1.63
	≥70	1030.10 (958.44 to 1113.91)	1354.94 (1251.16 to 1464.48)	820.39 (743.05 to 918.56)	1.65
East Asia	0-14	0.00 (0.00 to 0.00)	0.00 (0.00 to 0.00)	0.00 (0.00 to 0.00)	NA
	15-49	153.62 (130.78 to 183.76)	210.68 (167.56 to 259.36)	91.43 (73.16 to 121.81)	2.30
	50-69	813.79 (695.13 to 969.73)	1112.05 (914.12 to 1300.54)	516.13 (413.36 to 694.43)	2.15
	≥70	1602.53 (1327.35 to 1986.29)	2034.74 (1695.76 to 2491.02)	1235.83 (950.41 to 1692.78)	1.65
High-income Asia Pacific	0-14	0.00 (0.00 to 0.00)	0.00 (0.00 to 0.00)	0.00 (0.00 to 0.00)	NA
	15-49	133.18 (122.74 to 143.29)	143.79 (128.67 to 161.75)	121.98 (110.00 to 132.81)	1.18
	50-69	979.30 (909.83 to 1046.44)	1291.57 (1206.36 to 1394.75)	669.33 (604.58 to 728.16)	1.93
	≥70	2759.99 (2305.98 to 3058.41)	3478.62 (3121.45 to 3741.31)	2237.65 (1673.23 to 2602.99)	1.55
South Asia	0-14	0.00 (0.00 to 0.00)	0.00 (0.00 to 0.00)	0.00 (0.00 to 0.00)	NA
	15-49	68.52 (52.21 to 85.48)	61.13 (43.34 to 80.88)	76.20 (53.56 to 105.63)	0.80
	50-69	445.97 (350.53 to 551.33)	444.69 (330.06 to 597.05)	447.26 (322.54 to 608.91)	0.99
	≥70	609.89 (488.86 to 761.44)	648.23 (467.98 to 888.23)	575.00 (417.87 to 767.36)	1.13
Southeast Asia	0-14	0.00 (0.00 to 0.00)	0.00 (0.00 to 0.00)	0.00 (0.00 to 0.00)	NA
	15-49	185.96 (146.50 to 234.62)	211.18 (146.36 to 284.88)	160.01 (117.36 to 212.44)	1.32

	50-69	1079.47 (877.48 to 1299.95)	1402.61 (1045.66 to 1777.78)	777.33 (601.78 to 1000.84)	1.80
	≥70	1420.44 (1160.62 to 1703.15)	1893.59 (1457.98 to 2339.94)	1068.14 (845.50 to 1353.70)	1.77

DALY: Disability-Adjusted Life Year

Table S16. Age-specific incidence rates and male-to-female ratios of liver cancer by region in Asia, 2023

location	Age Group	Both	Male	Female	M/F
Central Asia	0-14	0.26 (0.21 to 0.32)	0.28 (0.21 to 0.37)	0.24 (0.17 to 0.32)	1.19
	15-49	1.48 (1.31 to 1.70)	1.88 (1.56 to 2.26)	1.07 (0.87 to 1.31)	1.76
	50-69	20.17 (18.35 to 22.60)	28.50 (24.83 to 32.51)	12.91 (11.26 to 15.01)	2.21
	≥70	40.35 (36.46 to 45.56)	50.81 (44.88 to 58.70)	33.61 (27.85 to 40.04)	1.51
East Asia	0-14	0.42 (0.35 to 0.52)	0.48 (0.37 to 0.63)	0.35 (0.26 to 0.46)	1.37
	15-49	4.40 (3.64 to 5.33)	7.40 (5.84 to 9.17)	1.13 (0.89 to 1.44)	6.56
	50-69	21.95 (18.31 to 26.35)	34.56 (27.51 to 43.03)	9.37 (7.50 to 11.69)	3.69
	≥70	36.11 (29.59 to 42.09)	47.16 (37.96 to 58.29)	26.73 (19.93 to 33.97)	1.76
High-income Asia Pacific	0-14	0.33 (0.27 to 0.40)	0.37 (0.29 to 0.47)	0.28 (0.23 to 0.36)	1.30
	15-49	2.21 (1.84 to 2.69)	3.36 (2.69 to 4.27)	0.99 (0.78 to 1.25)	3.39
	50-69	29.32 (25.21 to 33.93)	48.18 (40.69 to 56.64)	10.60 (8.52 to 12.84)	4.54
	≥70	117.82 (98.16 to 133.62)	177.15 (155.17 to 197.88)	74.69 (54.81 to 90.23)	2.37
South Asia	0-14	0.06 (0.04 to 0.09)	0.07 (0.03 to 0.11)	0.06 (0.03 to 0.10)	1.09
	15-49	0.92 (0.63 to 1.33)	1.27 (0.74 to 1.99)	0.57 (0.32 to 0.91)	2.24
	50-69	9.21 (6.43 to 12.90)	12.88 (7.79 to 19.89)	5.52 (3.20 to 8.57)	2.33
	≥70	16.08 (11.21 to 22.27)	20.00 (11.41 to 30.93)	12.52 (7.20 to 19.86)	1.60
Southeast Asia	0-14	0.24 (0.17 to 0.35)	0.32 (0.18 to 0.51)	0.17 (0.10 to 0.27)	1.91
	15-49	2.86 (2.10 to 3.76)	4.51 (3.21 to 6.36)	1.16 (0.74 to 1.78)	3.90
	50-69	23.36 (18.62 to 29.98)	38.39 (30.03 to 51.67)	9.30 (6.21 to 13.90)	4.13
	≥70	34.53 (27.24 to 42.96)	51.16 (38.08 to 68.17)	22.15 (16.62 to 29.93)	2.31

Table S17. Age-specific death rates and male-to-female ratios of liver cancer by region in Asia, 2023

Location	Age Group	Both	Male	Female	M/F
Central Asia	0-14	0.15 (0.12 to 0.19)	0.16 (0.12 to 0.21)	0.14 (0.10 to 0.19)	1.17
	15-49	1.22 (1.09 to 1.41)	1.57 (1.29 to 1.89)	0.88 (0.71 to 1.07)	1.78

	50-69	19.11 (17.40 to 21.38)	26.88 (23.48 to 30.76)	12.34 (10.81 to 14.29)	2.18
	≥70	46.37 (41.68 to 51.98)	57.38 (50.30 to 66.69)	39.26 (32.58 to 46.38)	1.46
East Asia	0-14	0.11 (0.09 to 0.13)	0.12 (0.09 to 0.16)	0.09 (0.07 to 0.12)	1.31
	15-49	3.26 (2.70 to 3.92)	5.52 (4.31 to 6.86)	0.80 (0.65 to 0.99)	6.87
	50-69	18.22 (15.21 to 21.70)	28.64 (23.06 to 35.37)	7.82 (6.31 to 9.76)	3.66
	≥70	37.12 (31.18 to 42.86)	48.09 (39.10 to 58.71)	27.80 (20.88 to 34.53)	1.73
High-income Asia Pacific	0-14	0.05 (0.04 to 0.06)	0.05 (0.04 to 0.07)	0.04 (0.03 to 0.05)	1.26
	15-49	1.34 (1.12 to 1.63)	2.11 (1.70 to 2.66)	0.53 (0.45 to 0.64)	3.95
	50-69	19.88 (17.09 to 23.40)	33.23 (28.46 to 39.97)	6.64 (5.64 to 7.89)	5.01
	≥70	95.33 (78.60 to 107.14)	141.35 (124.64 to 154.99)	61.87 (44.68 to 73.42)	2.28
South Asia	0-14	0.05 (0.03 to 0.07)	0.05 (0.02 to 0.07)	0.04 (0.02 to 0.07)	1.07
	15-49	0.75 (0.51 to 1.07)	1.03 (0.61 to 1.62)	0.45 (0.25 to 0.73)	2.30
	50-69	8.60 (5.97 to 12.07)	11.97 (7.27 to 18.57)	5.20 (3.02 to 7.94)	2.30
	≥70	18.06 (12.64 to 24.97)	22.24 (12.67 to 34.64)	14.26 (8.16 to 22.51)	1.56
Southeast Asia	0-14	0.15 (0.10 to 0.21)	0.19 (0.10 to 0.31)	0.10 (0.06 to 0.16)	1.90
	15-49	2.30 (1.71 to 3.01)	3.67 (2.60 to 5.20)	0.88 (0.58 to 1.42)	4.15
	50-69	21.27 (16.89 to 27.39)	35.13 (27.27 to 47.59)	8.30 (5.48 to 12.66)	4.23
	≥70	38.43 (30.44 to 47.71)	56.63 (42.07 to 75.56)	24.88 (18.74 to 33.94)	2.28

Table S18. Age-specific DALY rates and male-to-female ratios of liver cancer by region in Asia, 2023

Location	Age Group	Both	Male	Female	M/F
Central Asia	0-14	12.76 (10.21 to 16.06)	13.70 (10.27 to 18.08)	11.76 (8.36 to 16.35)	1.17
	15-49	60.07 (53.56 to 69.51)	75.65 (62.35 to 91.01)	44.26 (35.84 to 54.26)	1.71
	50-69	575.57 (522.87 to 643.50)	818.81 (713.54 to 939.45)	363.88 (317.87 to 422.03)	2.25
	≥70	752.66 (679.06 to 846.56)	963.92 (856.55 to 1116.35)	616.29 (511.70 to 728.14)	1.56
East Asia	0-14	9.31 (7.65 to 11.26)	10.46 (7.94 to 13.73)	8.01 (6.07 to 10.59)	1.31
	15-49	157.08 (131.04 to 187.88)	265.39 (208.80 to 328.55)	39.02 (31.80 to 47.84)	6.80
	50-69	564.96 (476.49 to 675.68)	897.86 (723.48 to 1113.10)	232.73 (188.63 to 291.18)	3.86
	≥70	585.72 (488.72 to 680.12)	776.94 (628.95 to 959.68)	423.48 (315.65 to 524.63)	1.83
High-income Asia Pacific	0-14	4.24 (3.44 to 5.18)	4.72 (3.68 to 6.05)	3.73 (2.95 to 4.80)	1.26
	15-49	62.80 (52.74 to 76.26)	98.08 (79.06 to 124.56)	25.54 (21.65 to 30.27)	3.84
	50-69	584.75 (501.01 to 692.57)	980.26 (840.53 to 1183.05)	192.15 (162.29 to 228.37)	5.10

	≥70	1278.68 (1087.01 to 1421.37)	1997.67 (1779.86 to 2178.90)	756.08 (568.38 to 883.89)	2.64
South Asia	0-14	3.94 (2.57 to 5.83)	4.06 (2.07 to 6.47)	3.81 (1.96 to 6.36)	1.07
	15-49	37.09 (25.26 to 53.13)	50.54 (29.69 to 79.03)	23.09 (12.85 to 37.12)	2.19
	50-69	262.23 (179.89 to 369.07)	368.00 (224.21 to 563.66)	155.92 (91.01 to 237.16)	2.36
	≥70	301.01 (209.92 to 420.61)	376.75 (214.88 to 583.74)	232.10 (132.83 to 370.21)	1.62
Southeast Asia	0-14	12.56 (8.49 to 18.23)	16.30 (8.96 to 26.75)	8.58 (4.91 to 14.04)	1.90
	15-49	111.84 (82.72 to 147.06)	177.82 (124.69 to 252.78)	43.96 (28.39 to 70.69)	4.04
	50-69	661.56 (527.35 to 850.71)	1100.72 (853.59 to 1496.03)	250.92 (166.37 to 383.32)	4.39
	≥70	627.83 (497.08 to 780.48)	950.36 (700.35 to 1269.90)	387.68 (289.86 to 535.28)	2.45

DALY: Disability-Adjusted Life Year

Table S19. Age-specific incidence rates and male-to-female ratios of pancreatic cancer by region in Asia, 2023

Location	Age Group	Both	Male	Female	M/F
Central Asia	0-14	0.00 (0.00 to 0.00)	0.00 (0.00 to 0.00)	0.00 (0.00 to 0.00)	NA
	15-49	0.85 (0.76 to 0.96)	1.11 (0.94 to 1.30)	0.59 (0.50 to 0.68)	1.89
	50-69	12.92 (11.70 to 14.09)	16.74 (14.72 to 18.93)	9.60 (8.30 to 10.95)	1.74
	≥70	30.74 (27.87 to 33.87)	37.46 (32.56 to 42.80)	26.40 (22.38 to 30.25)	1.42
East Asia	0-14	0.00 (0.00 to 0.00)	0.00 (0.00 to 0.00)	0.00 (0.00 to 0.00)	NA
	15-49	1.40 (1.15 to 1.67)	2.01 (1.58 to 2.52)	0.72 (0.56 to 0.90)	2.78
	50-69	14.47 (12.31 to 16.68)	19.23 (15.84 to 22.97)	9.72 (7.89 to 11.91)	1.98
	≥70	38.73 (31.98 to 47.03)	44.97 (36.67 to 55.80)	33.44 (25.27 to 44.33)	1.34
High-income Asia Pacific	0-14	0.00 (0.00 to 0.00)	0.00 (0.00 to 0.00)	0.00 (0.00 to 0.00)	NA
	15-49	1.55 (1.43 to 1.68)	1.88 (1.69 to 2.09)	1.19 (1.08 to 1.34)	1.58
	50-69	26.06 (24.25 to 27.66)	33.08 (31.03 to 35.61)	19.10 (17.16 to 20.81)	1.73
	≥70	148.59 (118.32 to 167.87)	159.11 (141.17 to 171.94)	140.95 (102.14 to 164.51)	1.13
South Asia	0-14	0.00 (0.00 to 0.00)	0.00 (0.00 to 0.00)	0.00 (0.00 to 0.00)	NA
	15-49	0.37 (0.28 to 0.47)	0.46 (0.31 to 0.65)	0.28 (0.19 to 0.38)	1.64
	50-69	5.78 (4.29 to 7.30)	6.87 (4.64 to 9.40)	4.68 (3.28 to 6.60)	1.47
	≥70	13.54 (10.41 to 17.26)	14.61 (9.71 to 21.07)	12.56 (9.04 to 17.43)	1.16
Southeast Asia	0-14	0.00 (0.00 to 0.00)	0.00 (0.00 to 0.00)	0.00 (0.00 to 0.00)	NA
	15-49	1.08 (0.87 to 1.33)	1.33 (1.02 to 1.72)	0.84 (0.60 to 1.11)	1.59
	50-69	11.45 (9.48 to 13.60)	14.25 (11.29 to 17.73)	8.85 (6.72 to 11.38)	1.61

	≥70	22.44 (18.74 to 26.24)	24.90 (20.29 to 30.94)	20.60 (15.97 to 26.15)	1.21

Table S20. Age-specific death rates and male-to-female ratios of pancreatic cancer by region in Asia, 2023

Location	Age Group	Both	Male	Female	M/F
Central Asia	0-14	0.00 (0.00 to 0.00)	0.00 (0.00 to 0.00)	0.00 (0.00 to 0.00)	NA
	15-49	0.70 (0.62 to 0.79)	0.92 (0.78 to 1.08)	0.48 (0.40 to 0.55)	1.94
	50-69	12.23 (11.05 to 13.30)	15.87 (13.97 to 17.94)	9.07 (7.84 to 10.32)	1.75
	≥70	34.20 (30.96 to 37.62)	41.54 (36.09 to 47.50)	29.45 (24.96 to 33.56)	1.41
East Asia	0-14	0.00 (0.00 to 0.00)	0.00 (0.00 to 0.00)	0.00 (0.00 to 0.00)	NA
	15-49	1.12 (0.92 to 1.34)	1.63 (1.28 to 2.05)	0.56 (0.44 to 0.69)	2.93
	50-69	13.41 (11.47 to 15.46)	17.88 (14.70 to 21.36)	8.95 (7.32 to 11.01)	2.00
	≥70	40.59 (33.86 to 48.98)	47.06 (38.07 to 57.66)	35.11 (27.06 to 46.52)	1.34
High-income Asia Pacific	0-14	0.00 (0.00 to 0.00)	0.00 (0.00 to 0.00)	0.00 (0.00 to 0.00)	NA
	15-49	1.20 (1.11 to 1.30)	1.49 (1.34 to 1.66)	0.9 (0.81 to 1.00)	1.66
	50-69	23.01 (21.58 to 24.15)	29.34 (27.41 to 31.46)	16.72 (14.95 to 18.01)	1.75
	≥70	131.86 (105.61 to 147.58)	141.46 (128.18 to 151.22)	124.88 (91.06 to 147.12)	1.13
South Asia	0-14	0.00 (0.00 to 0.00)	0.00 (0.00 to 0.00)	0.00 (0.00 to 0.00)	NA
	15-49	0.30 (0.23 to 0.39)	0.38 (0.26 to 0.54)	0.22 (0.15 to 0.30)	1.71
	50-69	5.44 (4.06 to 6.86)	6.49 (4.36 to 8.89)	4.40 (3.09 to 6.19)	1.48
	≥70	15.04 (11.64 to 19.09)	16.37 (10.98 to 23.69)	13.83 (9.97 to 19.13)	1.18
Southeast Asia	0-14	0.00 (0.00 to 0.00)	0.00 (0.00 to 0.00)	0.00 (0.00 to 0.00)	NA
	15-49	0.88 (0.70 to 1.08)	1.08 (0.83 to 1.40)	0.67 (0.48 to 0.90)	1.62
	50-69	10.76 (8.88 to 12.77)	13.37 (10.59 to 16.61)	8.32 (6.31 to 10.72)	1.61
	≥70	25.08 (21.05 to 29.33)	27.69 (22.50 to 34.49)	23.14 (17.81 to 29.47)	1.20

Table S21. Age-specific DALY rates and male-to-female ratios of pancreatic cancer by region in Asia, 2023

Location	Age Group	Both	Male	Female	M/F
Central Asia	0-14	0.00 (0.00 to 0.00)	0.00 (0.00 to 0.00)	0.00 (0.00 to 0.00)	NA
	15-49	33.70 (29.94 to 37.86)	44.25 (37.43 to 51.84)	22.99 (19.51 to 26.45)	1.92
	50-69	364.99 (329.55 to 397.80)	477.15 (418.23 to 536.81)	267.38 (230.67 to 305.62)	1.78
	≥70	549.92 (497.73 to 600.89)	687.58 (597.70 to 783.74)	461.05 (392.32 to 527.25)	1.49

East Asia	0-14	0.00 (0.00 to 0.00)	0.00 (0.00 to 0.00)	0.00 (0.00 to 0.00)	NA
	15-49	52.82 (43.49 to 63.01)	77.10 (60.80 to 96.31)	26.34 (20.84 to 32.51)	2.93
	50-69	399.41 (341.46 to 459.53)	536.10 (444.69 to 634.83)	262.99 (214.56 to 323.30)	2.04
	≥70	635.53 (532.22 to 770.16)	754.66 (614.97 to 928.10)	534.46 (411.89 to 706.02)	1.41
High-income Asia Pacific	0-14	0.00 (0.00 to 0.00)	0.00 (0.00 to 0.00)	0.00 (0.00 to 0.00)	NA
	15-49	55.39 (51.16 to 59.84)	68.49 (61.42 to 76.29)	41.56 (37.74 to 46.27)	1.65
	50-69	662.56 (620.07 to 697.15)	846.86 (791.56 to 908.05)	479.62 (431.61 to 516.20)	1.77
	≥70	1776.43 (1470.71 to 1960.58)	2047.24 (1875.62 to 2176.01)	1579.58 (1193.55 to 1827.91)	1.30
South Asia	0-14	0.00 (0.00 to 0.00)	0.00 (0.00 to 0.00)	0.00 (0.00 to 0.00)	NA
	15-49	14.47 (10.98 to 18.52)	17.92 (12.38 to 25.53)	10.88 (7.44 to 14.82)	1.65
	50-69	163.16 (120.58 to 207.52)	196.38 (133.24 to 268.88)	129.77 (91.17 to 182.39)	1.51
	≥70	244.33 (187.09 to 312.05)	268.41 (177.39 to 385.99)	222.43 (160.12 to 306.14)	1.21
Southeast Asia	0-14	0.00 (0.00 to 0.00)	0.00 (0.00 to 0.00)	0.00 (0.00 to 0.00)	NA
	15-49	42.31 (33.53 to 52.18)	52.09 (39.73 to 67.44)	32.25 (23.24 to 43.24)	1.62
	50-69	328.39 (270.90 to 389.38)	412.14 (324.23 to 515.95)	250.08 (188.95 to 324.17)	1.65
	≥70	407.21 (344.70 to 477.29)	463.23 (379.05 to 576.12)	365.51 (284.83 to 461.42)	1.27

DALY: Disability-Adjusted Life Year

Table S22. Age-specific incidence rates and male-to-female ratios of gallbladder and biliary tract cancer by region in Asia, 2023

Location	Age Group	Both	Male	Female	M/F
Central Asia	0-14	0.00 (0.00 to 0.00)	0.00 (0.00 to 0.00)	0.00 (0.00 to 0.00)	NA
	15-49	0.10 (0.09 to 0.12)	0.10 (0.08 to 0.12)	0.11 (0.09 to 0.12)	0.93
	50-69	2.05 (1.83 to 2.32)	2.09 (1.75 to 2.47)	2.01 (1.68 to 2.39)	1.04
	≥70	5.44 (4.83 to 6.14)	5.50 (4.51 to 6.59)	5.40 (4.45 to 6.43)	1.02
East Asia	0-14	0.00 (0.00 to 0.00)	0.00 (0.00 to 0.00)	0.00 (0.00 to 0.00)	NA
	15-49	0.42 (0.32 to 0.56)	0.54 (0.37 to 0.78)	0.29 (0.21 to 0.44)	1.82
	50-69	4.88 (3.85 to 6.78)	5.67 (4.27 to 8.73)	4.08 (2.80 to 6.49)	1.39
	≥70	17.61 (13.73 to 23.28)	18.37 (13.56 to 28.49)	16.97 (11.43 to 23.00)	1.08
High-income Asia Pacific	0-14	0.00 (0.00 to 0.00)	0.00 (0.00 to 0.00)	0.00 (0.00 to 0.00)	NA
	15-49	0.59 (0.51 to 0.73)	0.66 (0.55 to 0.87)	0.51 (0.43 to 0.64)	1.28
	50-69	11.49 (10.26 to 13.65)	14.76 (12.76 to 18.05)	8.24 (6.84 to 10.24)	1.79
	≥70	100.70 (79.86 to 115.81)	119.75 (104.99 to 135.86)	86.84 (59.96 to 107.44)	1.38

South Asia	0-14	0.00 (0.00 to 0.00)	0.00 (0.00 to 0.00)	0.00 (0.00 to 0.00)	NA
	15-49	0.48 (0.34 to 0.66)	0.35 (0.21 to 0.51)	0.62 (0.38 to 0.94)	0.56
	50-69	7.81 (5.62 to 10.44)	5.60 (3.53 to 8.19)	10.03 (6.38 to 14.53)	0.56
	≥70	19.63 (13.91 to 26.30)	15.36 (9.20 to 23.38)	23.52 (15.00 to 34.36)	0.65
Southeast Asia	0-14	0.00 (0.00 to 0.00)	0.00 (0.00 to 0.00)	0.00 (0.00 to 0.00)	NA
	15-49	0.40 (0.32 to 0.56)	0.50 (0.36 to 0.71)	0.30 (0.22 to 0.51)	1.67
	50-69	5.99 (4.78 to 8.74)	7.84 (5.94 to 12.66)	4.25 (3.30 to 7.39)	1.85
	≥70	16.52 (13.21 to 23.74)	21.17 (15.78 to 33.53)	13.06 (10.05 to 21.26)	1.62

Table S23. Age-specific death rates and male-to-female ratios of gallbladder and biliary tract cancer by region in Asia, 2023

Location	Age Group	Both	Male	Female	M/F
Central Asia	0-14	0.00 (0.00 to 0.00)	0.00 (0.00 to 0.00)	0.00 (0.00 to 0.00)	NA
	15-49	0.08 (0.07 to 0.10)	0.08 (0.07 to 0.10)	0.09 (0.07 to 0.10)	0.91
	50-69	1.92 (1.71 to 2.17)	1.95 (1.63 to 2.30)	1.91 (1.59 to 2.27)	1.02
	≥70	6.13 (5.45 to 6.95)	6.08 (5.01 to 7.27)	6.15 (5.08 to 7.36)	0.99
East Asia	0-14	0.00 (0.00 to 0.00)	0.00 (0.00 to 0.00)	0.00 (0.00 to 0.00)	NA
	15-49	0.24 (0.18 to 0.32)	0.29 (0.21 to 0.44)	0.17 (0.12 to 0.26)	1.69
	50-69	3.33 (2.70 to 4.58)	3.74 (2.83 to 5.79)	2.93 (2.07 to 4.42)	1.28
	≥70	14.20 (11.17 to 18.81)	14.35 (10.44 to 21.05)	14.08 (9.68 to 18.71)	1.02
High-income Asia Pacific	0-14	0.00 (0.00 to 0.00)	0.00 (0.00 to 0.00)	0.00 (0.00 to 0.00)	NA
	15-49	0.33 (0.29 to 0.41)	0.37 (0.31 to 0.50)	0.29 (0.25 to 0.35)	1.28
	50-69	7.60 (6.79 to 8.94)	9.71 (8.55 to 12.01)	5.50 (4.59 to 6.83)	1.76
	≥70	75.40 (59.46 to 86.49)	88.06 (76.56 to 97.26)	66.20 (44.71 to 81.21)	1.33
South Asia	0-14	0.00 (0.00 to 0.00)	0.00 (0.00 to 0.00)	0.00 (0.00 to 0.00)	NA
	15-49	0.39 (0.27 to 0.53)	0.28 (0.17 to 0.41)	0.50 (0.30 to 0.76)	0.56
	50-69	7.29 (5.26 to 9.72)	5.18 (3.26 to 7.54)	9.40 (5.99 to 13.67)	0.55
	≥70	22.42 (15.91 to 30.00)	17.38 (10.44 to 26.76)	27.01 (17.23 to 39.55)	0.64
Southeast Asia	0-14	0.00 (0.00 to 0.00)	0.00 (0.00 to 0.00)	0.00 (0.00 to 0.00)	NA
	15-49	0.29 (0.23 to 0.41)	0.35 (0.25 to 0.50)	0.22 (0.16 to 0.37)	1.60
	50-69	5.07 (4.11 to 7.45)	6.57 (4.88 to 10.76)	3.68 (2.83 to 6.35)	1.79
	≥70	17.15 (13.40 to 24.84)	21.58 (16.44 to 34.85)	13.84 (10.75 to 22.59)	1.56

Table S24. Age-specific DALY rates and male-to-female ratios of gallbladder and biliary tract cancer by region in Asia, 2023

Location	Age Group	Both	Male	Female	M/F
Central Asia	0-14	0.00 (0.00 to 0.00)	0.00 (0.00 to 0.00)	0.00 (0.00 to 0.00)	NA
	15-49	3.93 (3.45 to 4.49)	3.75 (3.11 to 4.53)	4.12 (3.46 to 4.87)	0.91
	50-69	57.21 (51.03 to 64.56)	58.06 (48.71 to 68.82)	56.46 (47.24 to 67.16)	1.03
	≥70	98.57 (88.03 to 111.57)	100.77 (82.91 to 119.94)	97.15 (80.58 to 115.99)	1.04
East Asia	0-14	0.00 (0.00 to 0.00)	0.00 (0.00 to 0.00)	0.00 (0.00 to 0.00)	NA
	15-49	11.18 (8.65 to 15.25)	13.96 (9.93 to 20.87)	8.15 (5.73 to 12.22)	1.71
	50-69	99.09 (80.76 to 136.54)	112.19 (85.10 to 175.44)	86.02 (61.22 to 129.28)	1.30
	≥70	216.19 (172.14 to 291.50)	222.24 (162.55 to 339.48)	211.05 (145.96 to 282.42)	1.05
High-income Asia Pacific	0-14	0.00 (0.00 to 0.00)	0.00 (0.00 to 0.00)	0.00 (0.00 to 0.00)	NA
	15-49	15.37 (13.70 to 19.16)	17.14 (14.29 to 23.18)	13.49 (11.57 to 16.50)	1.27
	50-69	215.71 (192.69 to 255.08)	274.40 (240.55 to 340.50)	157.45 (131.05 to 195.97)	1.74
	≥70	946.11 (769.93 to 1069.77)	1184.93 (1043.07 to 1305.48)	772.52 (540.50 to 935.75)	1.53
South Asia	0-14	0.00 (0.00 to 0.00)	0.00 (0.00 to 0.00)	0.00 (0.00 to 0.00)	NA
	15-49	18.60 (13.09 to 25.49)	13.32 (8.21 to 19.72)	24.10 (14.68 to 36.66)	0.55
	50-69	218.39 (157.22 to 287.83)	155.25 (96.95 to 225.54)	281.84 (179.73 to 405.69)	0.55
	≥70	355.95 (251.27 to 476.69)	277.28 (166.04 to 417.08)	427.52 (271.69 to 633.25)	0.65
Southeast Asia	0-14	0.00 (0.00 to 0.00)	0.00 (0.00 to 0.00)	0.00 (0.00 to 0.00)	NA
	15-49	13.49 (10.68 to 19.22)	16.41 (11.87 to 23.59)	10.49 (7.76 to 17.71)	1.56
	50-69	152.80 (123.45 to 226.17)	198.99 (147.22 to 325.71)	109.61 (84.91 to 190.83)	1.82
	≥70	273.54 (215.54 to 392.54)	352.14 (267.79 to 559.44)	215.01 (168.38 to 345.77)	1.64

DALY: Disability-Adjusted Life Year

Table S25. Incidence, death, DALYs, age-standardized rates, and changes from 1990 to 2023 for esophageal cancer in Asian countries and regions

	2023 Incidence (95% UI)	2023 ASIR Per 100 000 (95% UI)	1990 to 2023 EAPC (95% CI)	2023 Death (95% UI)	2023 ASDR Per 100 000 (95% UI)	1990 to 2023 EAPC (95% CI)	2023 DALYs (95% UI)	2023 ASDALYR Per 100 000 (95% UI)	1990 to 2023 EAPC (95% CI)
**Central Asia**									
Armenia	30 (26 to 35)	0.57 (0.50 to 0.65)	-3.84 (-4.14 to -3.55)	31 (28 to 36)	0.59 (0.52 to 0.67)	-3.87 (-4.16 to -3.58)	700 (611 to 798)	13.54 (11.83 to 15.44)	-3.96 (-4.26 to -3.66)

Azerbaijan	605 (492 to 723)	5.27 (4.25 to 6.36)	-1.64 (-1.85 to -1.42)	600 (487 to 721)	5.42 (4.32 to 6.59)	-1.53 (-1.74 to -1.31)	15,796 (12,813 to 18,903)	130.21 (107.09 to 156.37)	-1.95 (-2.19 to -1.72)
Georgia	65 (55 to 77)	1.06 (0.90 to 1.26)	-1.19 (-1.65 to -0.72)	66 (56 to 78)	1.07 (0.91 to 1.27)	-1.20 (-1.67 to -0.73)	1574 (1318 to 1875)	26.72 (22.46 to 31.76)	-1.27 (-1.73 to -0.82)
Kazakhstan	847 (774 to 914)	4.01 (3.65 to 4.31)	-4.69 (-4.94 to -4.44)	838 (768 to 907)	4.07 (3.71 to 4.40)	-4.74 (-5.01 to -4.47)	21,607 (19,996 to 23,231)	98.90 (91.18 to 106.03)	-4.79 (-5.02 to -4.57)
Kyrgyzstan	139 (124 to 153)	2.74 (2.45 to 3.02)	-3.63 (-3.83 to -3.43)	138 (124 to 152)	2.86 (2.58 to 3.16)	-3.55 (-3.76 to -3.34)	3587 (3200 to 3955)	64.75 (58.06 to 71.16)	-3.96 (-4.16 to -3.76)
Mongolia	359 (294 to 450)	15.49 (12.79 to 19.48)	-1.86 (-2.03 to -1.70)	359 (295 to 453)	16.43 (13.48 to 20.91)	-1.80 (-1.96 to -1.63)	9451 (7720 to 11,874)	366.52 (302.86 to 462.56)	-2.05 (-2.23 to -1.87)
Tajikistan	275 (213 to 486)	4.98 (3.86 to 8.58)	-4.20 (-4.72 to -3.68)	272 (209 to 481)	5.25 (4.06 to 8.98)	-4.06 (-4.56 to -3.54)	7249 (5582 to 12,719)	117.64 (91.27 to 205.40)	-4.57 (-5.10 to -4.03)
Turkmenistan	382 (339 to 430)	8.60 (7.63 to 9.59)	-3.91 (-4.49 to -3.33)	374 (332 to 420)	8.72 (7.77 to 9.72)	-3.90 (-4.48 to -3.33)	10,360 (9195 to 11,610)	219.10 (194.80 to 244.47)	-3.95 (-4.52 to -3.37)
Uzbekistan	1135 (1076 to 1202)	4.26 (4.05 to 4.52)	-4.38 (-4.91 to -3.85)	1110 (1053 to 1177)	4.36 (4.15 to 4.64)	-4.39 (-4.92 to -3.85)	30,132 (28,525 to 31,745)	104.35 (99.33 to 110.47)	-4.50 (-5.01 to -3.99)
**East Asia**									
China	267,432 (231,585 to 306,644)	11.45 (9.83 to 13.10)	-3.42 (-3.75 to -3.09)	254,463 (217,044 to 293,116)	10.95 (9.35 to 12.55)	-3.58 (-3.92 to -3.24)	5,734,334 (5,077,392 to 6,554,933)	244.50 (216.48 to 278.63)	-3.96 (-4.31 to -3.60)
Democratic People's Republic of Korea	3677 (2552 to 5226)	10.81 (7.57 to 15.51)	-0.08 (-0.18 to 0.03)	3486 (2434 to 4988)	10.45 (7.27 to 15.03)	-0.21 (-0.32 to -0.11)	90,583 (61,571 to 128,474)	257.42 (178.24 to 368.73)	-0.28 (-0.38 to -0.19)
**High-income Asia Pacific**									
Brunei Darussalam	9 (7 to 11)	2.05 (1.60 to 2.57)	-1.12 (-1.18 to -1.07)	8 (6 to 10)	1.88 (1.44 to 2.36)	-1.31 (-1.37 to -1.26)	213 (163 to 263)	45.65 (34.89 to 56.63)	-1.29 (-1.35 to -1.22)
Japan	22,612 (20,015 to 24,387)	6.21 (5.61 to 6.66)	-0.42 (-0.67 to -0.17)	15,204 (13,362 to 16,449)	3.86 (3.50 to 4.15)	-1.04 (-1.18 to -0.91)	271,603 (244,361 to 291,517)	84.06 (77.25 to 89.98)	-1.37 (-1.57 to -1.18)
Republic of Korea	3683 (3155 to 4458)	3.58 (3.06 to 4.35)	-2.41 (-2.63 to -2.19)	2451 (2143 to 2882)	2.37 (2.07 to 2.80)	-3.61 (-3.86 to -3.35)	52,074 (45,836 to 62,958)	50.97 (45.04 to 61.97)	-4.21 (-4.50 to -3.91)

Singapore	287 (241 to 333)	3.06 (2.57 to 3.55)	-2.39 (-2.58 to -2.19)	190 (164 to 214)	2.03 (1.75 to 2.29)	-3.47 (-3.67 to -3.27)	4254 (3692 to 4815)	45.08 (39.16 to 50.94)	-3.60 (-3.79 to -3.40)
**South Asia**									
Bangladesh	14,704 (9628 to 22,178)	10.93 (7.14 to 16.43)	1.16 (0.91 to 1.41)	13,908 (9074 to 20,945)	10.84 (7.05 to 16.28)	1.19 (0.97 to 1.41)	408,047 (263,960 to 618,392)	284.17 (184.48 to 429.62)	0.90 (0.60 to 1.20)
Bhutan	42 (25 to 64)	6.20 (3.74 to 9.43)	0.43 (0.29 to 0.56)	41 (25 to 61)	6.11 (3.70 to 9.22)	0.32 (0.19 to 0.45)	1182 (708 to 1800)	166.91 (100.38 to 252.96)	0.49 (0.35 to 0.62)
India	97,321 (76,412 to 122,702)	7.77 (6.09 to 9.76)	2.21 (1.64 to 2.78)	95,871 (75,278 to 121,011)	7.95 (6.19 to 9.95)	2.25 (1.68 to 2.83)	2,587,556 (2,001,398 to 3,270,075)	195.92 (151.70 to 248.24)	2.02 (1.47 to 2.58)
Nepal	1292 (856 to 1941)	4.90 (3.25 to 7.37)	0.02 (-0.19 to 0.23)	1270 (843 to 1902)	4.97 (3.32 to 7.37)	0.00 (-0.21 to 0.20)	35,153 (23,164 to 52,531)	127.91 (84.28 to 191.42)	-0.01 (-0.22 to 0.20)
Pakistan	11,511 (8538 to 15,751)	8.02 (5.98 to 11.02)	0.31 (0.20 to 0.43)	11,113 (8286 to 15,300)	7.99 (5.96 to 10.98)	0.26 (0.14 to 0.37)	325,430 (242,246 to 448,601)	214.97 (159.38 to 295.57)	0.34 (0.23 to 0.46)
**Southeast Asia**									
Cambodia	291 (208 to 425)	2.04 (1.46 to 2.97)	-0.69 (-0.78 to -0.61)	282 (201 to 409)	2.04 (1.46 to 2.96)	-0.66 (-0.74 to -0.58)	8040 (5666 to 11,747)	53.69 (38.35 to 78.34)	-1.00 (-1.09 to -0.91)
Indonesia	6783 (4599 to 9720)	2.32 (1.58 to 3.32)	0.01 (-0.13 to 0.14)	6310 (4258 to 9034)	2.22 (1.51 to 3.17)	-0.15 (-0.28 to -0.01)	196,556 (132,870 to 279,631)	63.90 (43.36 to 90.79)	0.14 (0.00 to 0.27)
Lao People's Democratic Republic	167 (120 to 235)	3.12 (2.24 to 4.36)	0.44 (0.25 to 0.62)	158 (114 to 222)	3.08 (2.20 to 4.30)	0.35 (0.17 to 0.52)	4780 (3411 to 6880)	83.18 (59.84 to 117.59)	0.42 (0.22 to 0.62)
Malaysia	619 (522 to 748)	1.90 (1.59 to 2.30)	-1.03 (-1.19 to -0.88)	593 (501 to 716)	1.87 (1.57 to 2.26)	-1.21 (-1.38 to -1.05)	15,426 (13,025 to 18,584)	45.73 (38.67 to 55.23)	-1.03 (-1.17 to -0.90)
Maldives	8 (6 to 9)	2.57 (2.02 to 3.24)	-0.06 (-0.18 to 0.07)	7 (6 to 9)	2.58 (2.04 to 3.26)	-0.11 (-0.24 to 0.02)	172 (133 to 218)	51.59 (40.65 to 64.65)	-0.81 (-0.91 to -0.72)
Mauritius	53 (46 to 61)	2.80 (2.43 to 3.23)	-1.06 (-1.35 to -0.77)	50 (43 to 57)	2.67 (2.32 to 3.05)	-1.20 (-1.48 to -0.91)	1305 (1135 to 1508)	69.41 (60.31 to 80.46)	-1.12 (-1.40 to -0.83)
Myanmar	1658 (1147 to 2386)	3.18 (2.23 to 4.55)	-0.57 (-0.79 to -0.35)	1596 (1114 to 2298)	3.15 (2.23 to 4.51)	-0.61 (-0.82 to -0.41)	45,394 (31,249 to 65,119)	83.23 (57.86 to 119.20)	-0.71 (-0.93 to -0.49)
Philippines	1073 (841 to 1360)	1.20 (0.94 to 1.52)	-0.45 (-0.66 to -0.24)	1024 (804 to 1290)	1.20 (0.94 to 1.50)	-0.48 (-0.68 to -0.28)	29,466 (23,192 to 37,213)	31.05 (24.53 to 39.22)	-0.53 (-0.74 to -0.31)

Seychelles	7 (6 to 9)	5.56 (4.23 to 7.00)	-0.34 (-0.56 to -0.11)	7 (5 to 9)	5.31 (4.04 to 6.59)	-0.42 (-0.64 to -0.19)	194 (147 to 247)	144.46 (109.07 to 182.68)	-0.47 (-0.68 to -0.25)
Sri Lanka	1825 (1509 to 2243)	6.04 (4.98 to 7.41)	-0.22 (-0.45 to 0.01)	1697 (1414 to 2080)	5.60 (4.67 to 6.85)	-0.42 (-0.64 to -0.19)	42,603 (34,928 to 52,840)	142.53 (116.89 to 176.64)	-0.56 (-0.80 to -0.32)
Thailand	5940 (4979 to 7081)	5.30 (4.45 to 6.26)	-0.82 (-1.09 to -0.54)	5398 (4551 to 6428)	4.73 (3.97 to 5.58)	-1.11 (-1.38 to -0.84)	154,798 (129,286 to 184,853)	140.85 (117.50 to 167.93)	-0.84 (-1.13 to -0.56)
Timor-Leste	25 (18 to 36)	2.71 (1.92 to 3.91)	-0.82 (-1.02 to -0.63)	24 (17 to 35)	2.67 (1.89 to 3.84)	-0.96 (-1.14 to -0.77)	681 (481 to 973)	69.95 (49.01 to 101.10)	-0.76 (-0.98 to -0.54)
Viet Nam	3322 (1981 to 5228)	2.84 (1.69 to 4.46)	1.35 (1.22 to 1.47)	3046 (1788 to 4757)	2.64 (1.55 to 4.09)	1.12 (1.00 to 1.25)	89,056 (53,588 to 139,589)	74.50 (44.73 to 116.42)	1.37 (1.23 to 1.52)

ASIR: age-standardized incidence rate; EAPC: estimated annual percent change; ASDR: age-standardized death rate; ASDALYR: age-standardized disability-adjusted life years rate; UI: uncertainty interval; CI: confidence interval

Table S26. Incidence, death, DALYs, age-standardized rates, and changes from 1990 to 2023 for stomach cancer in Asian countries and regions

	2023 Incidence (95% UI)	2023 ASIR Per 100 000 (95% UI)	1990 to 2023 EAPC (95% CI)	2023 Death (95% UI)	2023 ASDR Per 100 000 (95% UI)	1990 to 2023 EAPC (95% CI)	2023 DALYs (95% UI)	2023 ASDALYR Per 100 000 (95% UI)	1990 to 2023 EAPC (95% CI)
**Central Asia**									
Armenia	380 (338 to 425)	7.43 (6.64 to 8.36)	-2.30 (-2.61 to -2.00)	370 (330 to 410)	7.13 (6.36 to 7.89)	-2.35 (-2.67 to -2.03)	8495 (7654 to 9432)	172.57 (155.96 to 192.42)	-2.82 (-3.10 to -2.53)
Azerbaijan	1843 (1372 to 2425)	15.75 (11.68 to 20.70)	-1.59 (-1.75 to -1.43)	1746 (1283 to 2264)	15.42 (11.38 to 19.84)	-1.57 (-1.72 to -1.42)	48,813 (35,942 to 64,243)	398.13 (293.63 to 521.20)	-1.97 (-2.15 to -1.80)
Georgia	663 (588 to 727)	10.97 (9.70 to 12.07)	-0.09 (-0.56 to 0.38)	649 (579 to 708)	10.60 (9.45 to 11.55)	-0.12 (-0.60 to 0.36)	15,415 (13,759 to 16,976)	266.03 (236.68 to 292.36)	-0.46 (-0.91 to -0.01)
Kazakhstan	2277 (2107 to 2468)	10.60 (9.79 to 11.50)	-3.31 (-3.46 to -3.16)	2118 (1955 to 2290)	10.08 (9.31 to 10.88)	-3.46 (-3.63 to -3.28)	57,882 (53,711 to 62,342)	263.12 (244.32 to 282.82)	-3.61 (-3.77 to -3.46)
Kyrgyzstan	763 (677 to 849)	13.99 (12.49 to 15.57)	-2.27 (-2.53 to -2.02)	712 (631 to 788)	13.65 (12.27 to 15.07)	-2.29 (-2.55 to -2.04)	20,236 (17,963 to 22,534)	343.37 (306.08 to 380.17)	-2.69 (-2.95 to -2.44)

Mongolia	984 (765 to 1190)	38.64 (29.95 to 46.13)	-1.70 (-1.87 to -1.53)	932 (731 to 1118)	38.62 (29.98 to 46.62)	-1.73 (-1.90 to -1.56)	27,350 (21,265 to 33,559)	972.56 (762.27 to 1166.58)	-1.89 (-2.05 to -1.73)
Tajikistan	1179 (942 to 1574)	19.82 (15.64 to 26.40)	-1.87 (-2.26 to -1.46)	1124 (890 to 1498)	19.96 (15.77 to 26.52)	-1.74 (-2.13 to -1.36)	32,293 (26,311 to 42,472)	486.33 (385.93 to 641.90)	-2.36 (-2.77 to -1.94)
Turkmenistan	444 (399 to 505)	9.64 (8.69 to 10.93)	-2.53 (-2.89 to -2.16)	420 (376 to 475)	9.39 (8.48 to 10.57)	-2.58 (-2.94 to -2.22)	12,484 (11,242 to 14,187)	255.37 (230.20 to 287.38)	-2.61 (-2.97 to -2.25)
Uzbekistan	2332 (2181 to 2530)	8.32 (7.78 to 8.97)	-2.79 (-2.99 to -2.59)	2178 (2040 to 2354)	8.10 (7.57 to 8.76)	-2.83 (-3.03 to -2.63)	63,719 (59,844 to 68,863)	210.22 (197.05 to 227.18)	-3.03 (-3.20 to -2.86)
**East Asia**									
China	575,712 (435,410 to 755,447)	25.04 (18.93 to 32.90)	-2.76 (-2.96 to -2.55)	381,500 (301,099 to 479,840)	16.62 (13.15 to 20.84)	-3.84 (-4.11 to -3.58)	8,909,848 (7,026,141 to 11,289,073)	390.14 (307.72 to 493.75)	-4.17 (-4.44 to -3.90)
Democratic People's Republic of Korea	7050 (4632 to 10,247)	20.90 (13.75 to 30.19)	-0.43 (-0.58 to -0.28)	6221 (4136 to 9144)	18.90 (12.60 to 28.00)	-0.71 (-0.85 to -0.58)	166,240 (112,191 to 235,429)	480.26 (324.36 to 685.14)	-0.71 (-0.85 to -0.58)
**High-income Asia Pacific**									
Brunei Darussalam	51 (37 to 64)	11.89 (8.67 to 15.05)	-2.46 (-2.61 to -2.32)	40 (29 to 48)	10.00 (7.53 to 12.26)	-2.76 (-2.88 to -2.63)	1126 (830 to 1371)	241.32 (178.96 to 294.88)	-2.75 (-2.91 to -2.60)
Japan	106,736 (87,905 to 119,455)	26.55 (22.87 to 29.47)	-2.94 (-3.03 to -2.84)	61,196 (48,960 to 69,083)	13.40 (11.33 to 14.92)	-3.10 (-3.18 to -3.02)	952,106 (805,093 to 1,056,907)	270.43 (236.41 to 298.21)	-3.57 (-3.65 to -3.48)
Republic of Korea	25,244 (20,192 to 42,711)	25.73 (20.65 to 43.06)	-3.24 (-3.38 to -3.10)	12,856 (10,301 to 21,107)	12.91 (10.40 to 20.99)	-5.15 (-5.38 to -4.93)	258,118 (212,293 to 444,977)	270.21 (223.76 to 457.95)	-5.74 (-5.94 to -5.53)
Singapore	868 (741 to 969)	9.54 (8.11 to 10.62)	-3.36 (-3.48 to -3.25)	443 (379 to 491)	4.83 (4.12 to 5.34)	-4.97 (-5.12 to -4.83)	9022 (7940 to 9992)	98.93 (87.26 to 109.08)	-5.32 (-5.45 to -5.19)
**South Asia**									
Bangladesh	16,576 (9652 to 24,691)	12.14 (7.04 to 17.86)	-0.24 (-0.42 to -0.05)	15,279 (8905 to 22,437)	11.70 (6.77 to 17.06)	-0.26 (-0.41 to -0.10)	464,403 (273,788 to 697,437)	317.13 (186.35 to 471.08)	-0.57 (-0.80 to -0.33)

Bhutan	59 (35 to 92)	8.48 (5.04 to 13.25)	-0.54 (-0.70 to -0.38)	55 (32 to 85)	8.11 (4.79 to 12.73)	-0.69 (-0.85 to -0.52)	1693 (999 to 2688)	233.22 (138.30 to 366.21)	-0.51 (-0.68 to -0.34)
India	85,400 (60,934 to 112,998)	6.66 (4.76 to 8.82)	-0.91 (-1.05 to -0.76)	81,279 (58,392 to 108,357)	6.53 (4.71 to 8.74)	-0.96 (-1.11 to -0.81)	2,285,422 (1,658,488 to 3,049,401)	170.18 (123.24 to 227.84)	-1.07 (-1.22 to -0.93)
Nepal	1665 (1057 to 2459)	6.24 (3.93 to 9.25)	-1.37 (-1.64 to -1.09)	1597 (1008 to 2380)	6.16 (3.82 to 9.21)	-1.42 (-1.70 to -1.14)	46,418 (29,573 to 67,802)	166.47 (105.57 to 245.01)	-1.41 (-1.66 to -1.16)
Pakistan	5991 (4184 to 8219)	4.05 (2.83 to 5.60)	-0.35 (-0.46 to -0.24)	5667 (3951 to 7796)	3.99 (2.78 to 5.50)	-0.43 (-0.54 to -0.32)	177,371 (123,572 to 235,670)	110.88 (77.27 to 150.86)	-0.33 (-0.44 to -0.22)
**Southeast Asia**									
Cambodia	847 (566 to 1195)	5.92 (3.96 to 8.38)	-1.52 (-1.65 to -1.39)	803 (538 to 1129)	5.82 (3.94 to 8.24)	-1.49 (-1.62 to -1.36)	23,537 (15,579 to 33,112)	155.12 (102.97 to 217.63)	-2.00 (-2.14 to -1.85)
Indonesia	19,754 (13,326 to 28,646)	6.91 (4.69 to 9.94)	-0.29 (-0.45 to -0.13)	17,925 (12,175 to 25,950)	6.52 (4.48 to 9.39)	-0.49 (-0.65 to -0.34)	562,920 (378,390 to 811,210)	185.77 (125.66 to 268.31)	-0.21 (-0.37 to -0.06)
Lao People's Democratic Republic	513 (349 to 709)	9.54 (6.44 to 13.08)	-0.27 (-0.44 to -0.10)	479 (326 to 657)	9.34 (6.37 to 12.94)	-0.37 (-0.52 to -0.21)	14,917 (10,160 to 20,805)	253.80 (172.67 to 349.50)	-0.36 (-0.56 to -0.17)
Malaysia	1702 (1386 to 2155)	5.32 (4.33 to 6.80)	-1.91 (-2.13 to -1.70)	1556 (1284 to 1981)	5.04 (4.16 to 6.54)	-2.20 (-2.44 to -1.97)	39,519 (32,357 to 49,721)	117.95 (96.46 to 148.42)	-2.12 (-2.30 to -1.94)
Maldives	19 (13 to 23)	6.44 (4.55 to 8.08)	-0.44 (-0.70 to -0.19)	16 (12 to 20)	6.04 (4.39 to 7.68)	-0.74 (-0.99 to -0.49)	381 (275 to 482)	116.04 (83.80 to 144.85)	-1.53 (-1.75 to -1.32)
Mauritius	162 (145 to 184)	8.78 (7.79 to 9.93)	-2.19 (-2.37 to -2.01)	146 (131 to 164)	7.99 (7.10 to 8.95)	-2.46 (-2.63 to -2.28)	3622 (3224 to 4099)	195.98 (174.77 to 221.49)	-2.40 (-2.58 to -2.21)
Myanmar	4693 (3205 to 6569)	9.12 (6.20 to 12.89)	-1.18 (-1.42 to -0.95)	4457 (3044 to 6214)	8.95 (6.10 to 12.46)	-1.24 (-1.46 to -1.02)	128,475 (87,851 to 181,322)	237.26 (162.04 to 334.93)	-1.42 (-1.66 to -1.18)
Philippines	3314 (2604 to 4074)	3.79 (3.00 to 4.62)	-1.04 (-1.18 to -0.90)	3098 (2455 to 3760)	3.73 (2.97 to 4.53)	-1.11 (-1.25 to -0.98)	88,634 (69,375 to 108,303)	94.08 (73.88 to 114.65)	-1.15 (-1.29 to -1.01)
Seychelles	9 (7 to 13)	7.18 (5.17 to 9.90)	-1.56 (-1.74 to -1.39)	8 (6 to 11)	6.55 (4.78 to 8.80)	-1.75 (-1.92 to -1.58)	221 (162 to 304)	170.04 (124.21 to 231.59)	-1.82 (-1.97 to -1.67)
Sri Lanka	2387 (1773 to 2993)	7.96 (5.95 to 9.99)	-1.07 (-1.28 to -0.87)	2039 (1535 to 2540)	6.79 (5.14 to 8.46)	-1.51 (-1.70 to -1.32)	50,535 (37,883 to 63,244)	171.14 (127.86 to 213.92)	-1.68 (-1.90 to -1.45)
Thailand	10,610 (8400 to 14,050)	9.68 (7.60 to 12.87)	-1.36 (-1.54 to -1.18)	8850 (7073 to 11,917)	7.90 (6.30 to 10.55)	-1.88 (-2.05 to -1.70)	238,805 (188,155 to 312,306)	226.13 (176.34 to 293.12)	-1.71 (-1.92 to -1.51)

Timor-Leste	74 (49 to 105)	7.88 (5.21 to 11.11)	-1.51 (-1.73 to -1.29)	70 (46 to 98)	7.80 (5.08 to 10.88)	-1.63 (-1.84 to -1.42)	1998 (1349 to 2815)	201.34 (135.42 to 281.55)	-1.50 (-1.76 to -1.24)
Viet Nam	12,941 (9329 to 17,383)	11.21 (8.09 to 15.03)	-1.08 (-1.27 to -0.90)	10,732 (7761 to 14,139)	9.43 (6.82 to 12.34)	-1.49 (-1.65 to -1.32)	317,495 (229,703 to 418,119)	268.68 (194.42 to 352.36)	-1.35 (-1.51 to -1.19)

ASIR: age-standardized incidence rate; EAPC: estimated annual percent change; ASDR: age-standardized death rate; ASDALYR: age-standardized disability-adjusted life years rate; UI: uncertainty interval; CI: confidence interval

Table S27. Incidence, death, DALYs, age-standardized rates, and changes from 1990 to 2023 for colorectal cancer in Asian countries and regions

	2023 Incidence (95% UI)	2023 ASIR Per 100 000 (95% UI)	1990 to 2023 EAPC (95% CI)	2023 Death (95% UI)	2023 ASDR Per 100 000 (95% UI)	1990 to 2023 EAPC (95% CI)	2023 DALYs (95% UI)	2023 ASDALYR Per 100 000 (95% UI)	1990 to 2023 EAPC (95% CI)
**Central Asia**									
Armenia	736 (633 to 838)	14.44 (12.48 to 16.52)	0.30 (0.05 to 0.54)	503 (453 to 548)	9.69 (8.75 to 10.65)	-0.19 (-0.44 to 0.06)	11,217 (10,114 to 12,325)	227.20 (204.85 to 249.44)	-0.71 (-0.93 to -0.50)
Azerbaijan	1246 (1078 to 1468)	10.62 (9.17 to 12.52)	0.35 (0.04 to 0.67)	875 (766 to 1024)	7.83 (6.82 to 9.19)	0.01 (-0.28 to 0.29)	25,051 (21,974 to 29,390)	206.67 (181.58 to 242.70)	-0.44 (-0.71 to -0.17)
Georgia	1051 (910 to 1179)	17.51 (15.08 to 19.63)	2.30 (1.86 to 2.74)	753 (663 to 838)	12.27 (10.81 to 13.60)	2.10 (1.62 to 2.58)	17,689 (15,548 to 19,550)	306.02 (269.27 to 338.44)	1.59 (1.19 to 2.00)
Kazakhstan	2884 (2565 to 3173)	13.67 (12.12 to 14.98)	-0.54 (-0.74 to -0.35)	1906 (1771 to 2035)	9.45 (8.73 to 10.10)	-1.24 (-1.49 to -0.99)	50,673 (47,284 to 53,915)	235.69 (219.95 to 250.63)	-1.49 (-1.68 to -1.29)
Kyrgyzstan	467 (413 to 524)	8.92 (7.88 to 10.10)	-1.02 (-1.19 to -0.84)	321 (290 to 356)	6.68 (6.10 to 7.42)	-1.44 (-1.59 to -1.28)	8943 (8062 to 9956)	159.07 (144.76 to 176.86)	-1.83 (-1.99 to -1.68)
Mongolia	290 (235 to 353)	11.12 (8.96 to 13.49)	-0.34 (-0.59 to -0.08)	210 (167 to 252)	8.73 (6.85 to 10.49)	-0.71 (-0.96 to -0.47)	6356 (5037 to 7719)	224.66 (178.55 to 270.17)	-0.91 (-1.14 to -0.68)
Tajikistan	512 (416 to 658)	8.48 (6.84 to 10.98)	-1.11 (-1.60 to -0.61)	380 (311 to 486)	6.84 (5.59 to 8.80)	-1.15 (-1.61 to -0.70)	11,155 (9061 to 14,189)	167.52 (138.02 to 211.83)	-1.80 (-2.27 to -1.33)
Turkmenistan	294 (257 to 336)	6.42 (5.65 to 7.27)	-0.50 (-1.02 to 0.03)	218 (193 to 245)	5.00 (4.44 to 5.61)	-0.76 (-1.26 to -0.25)	6584 (5810 to 7467)	136.08 (121.36 to 153.31)	-0.84 (-1.32 to -0.35)

Uzbekistan	2029 (1794 to 2310)	7.13 (6.33 to 8.05)	-0.38 (-0.87 to 0.12)	1399 (1258 to 1531)	5.24 (4.73 to 5.72)	-0.71 (-1.18 to -0.24)	41,410 (37,153 to 45,897)	136.74 (122.55 to 150.56)	-0.92 (-1.37 to -0.46)
**East Asia**									
China	611,364 (493,490 to 771,955)	26.60 (21.57 to 33.58)	0.46 (0.34 to 0.58)	258,088 (225,359 to 305,788)	11.45 (10.01 to 13.50)	-1.41 (-1.55 to -1.27)	6,279,382 (5,563,225 to 7,428,441)	282.23 (250.64 to 333.09)	-1.70 (-1.84 to -1.55)
Democratic People's Republic of Korea	7615 (5329 to 10,377)	22.60 (15.81 to 30.67)	1.48 (1.32 to 1.63)	4668 (3370 to 6361)	14.42 (10.35 to 19.88)	0.76 (0.64 to 0.88)	128,234 (92,313 to 171,447)	377.48 (272.75 to 508.15)	0.75 (0.64 to 0.87)
**High-income Asia Pacific**									
Brunei Darussalam	129 (97 to 160)	29.91 (22.60 to 37.11)	-0.06 (-0.18 to 0.05)	78 (62 to 95)	19.71 (15.68 to 23.92)	-0.62 (-0.74 to -0.50)	2228 (1760 to 2738)	479.14 (379.59 to 581.93)	-0.56 (-0.69 to -0.43)
Japan	195,100 (167,087 to 215,829)	53.92 (47.96 to 58.49)	0.39 (0.31 to 0.48)	75,292 (61,982 to 84,036)	17.20 (15.09 to 18.71)	-0.60 (-0.68 to -0.52)	1,283,575 (1,121,846 to 1,405,010)	387.17 (351.15 to 416.47)	-0.76 (-0.85 to -0.67)
Republic of Korea	37,891 (30,751 to 45,265)	37.78 (30.66 to 45.22)	1.71 (1.43 to 1.99)	13,695 (11,502 to 15,387)	13.66 (11.45 to 15.38)	-0.77 (-0.92 to -0.62)	272,585 (236,509 to 312,849)	279.24 (242.17 to 321.17)	-1.30 (-1.41 to -1.19)
Singapore	3632 (2992 to 4243)	39.23 (32.29 to 45.98)	-0.29 (-0.42 to -0.17)	1271 (1139 to 1385)	13.75 (12.33 to 14.96)	-2.34 (-2.47 to -2.22)	27,691 (25,165 to 30,188)	300.84 (274.26 to 327.43)	-2.51 (-2.62 to -2.40)
**South Asia**									
Bangladesh	13,513 (9529 to 18,126)	9.91 (6.97 to 13.36)	1.46 (1.17 to 1.74)	9722 (6769 to 13,028)	7.59 (5.32 to 10.28)	1.00 (0.79 to 1.22)	296,259 (209,222 to 390,404)	203.67 (143.86 to 269.51)	0.72 (0.41 to 1.04)
Bhutan	44 (32 to 60)	6.39 (4.64 to 8.61)	0.97 (0.88 to 1.06)	33 (24 to 44)	4.98 (3.58 to 6.60)	0.44 (0.35 to 0.53)	1020 (728 to 1378)	141.43 (101.88 to 189.88)	0.68 (0.57 to 0.78)
India	82,174 (65,520 to 100,472)	6.47 (5.18 to 7.90)	0.88 (0.71 to 1.05)	62,584 (49,953 to 76,194)	5.17 (4.15 to 6.29)	0.49 (0.35 to 0.64)	1,758,047 (1,394,054 to 2,143,552)	132.08 (104.96 to 160.75)	0.32 (0.17 to 0.48)
Nepal	1174 (864 to 1528)	4.40 (3.23 to 5.71)	0.43 (0.19 to 0.67)	939 (696 to 1223)	3.66 (2.68 to 4.76)	0.06 (-0.17 to 0.30)	27,351 (20,325 to 36,111)	98.23 (72.95 to 129.70)	0.15 (-0.07 to 0.37)

Pakistan	12,415 (9018 to 16,287)	8.37 (6.09 to 10.91)	1.42 (1.28 to 1.56)	9832 (7213 to 12,842)	6.96 (5.09 to 8.99)	1.12 (1.00 to 1.24)	308,398 (222,528 to 403,356)	194.37 (142.31 to 253.15)	1.23 (1.11 to 1.35)
**Southeast Asia**									
Cambodia	1564 (1100 to 2194)	10.90 (7.58 to 15.42)	1.44 (1.28 to 1.60)	1216 (859 to 1692)	8.88 (6.26 to 12.42)	1.14 (0.99 to 1.29)	35,430 (25,036 to 48,687)	235.12 (165.95 to 323.88)	0.66 (0.50 to 0.82)
Indonesia	42,484 (30,299 to 57,734)	14.67 (10.48 to 19.82)	2.25 (2.07 to 2.42)	30,268 (21,665 to 40,987)	11.01 (7.95 to 14.79)	1.70 (1.54 to 1.86)	962,115 (686,879 to 1,301,055)	317.44 (226.90 to 427.71)	2.04 (1.86 to 2.21)
Lao People's Democratic Republic	897 (643 to 1171)	16.59 (12.04 to 21.81)	2.65 (2.43 to 2.88)	694 (504 to 931)	13.59 (9.99 to 18.10)	2.20 (2.01 to 2.39)	21,637 (15,598 to 28,457)	369.83 (269.29 to 492.78)	2.27 (2.04 to 2.49)
Malaysia	7456 (6215 to 8752)	22.97 (19.29 to 26.82)	1.26 (1.09 to 1.43)	4871 (4059 to 5610)	15.81 (13.15 to 18.10)	0.39 (0.19 to 0.59)	123,937 (104,715 to 143,739)	371.22 (312.13 to 428.68)	0.67 (0.53 to 0.82)
Maldives	45 (33 to 56)	15.22 (11.48 to 18.91)	3.02 (2.92 to 3.12)	26 (20 to 32)	10.08 (7.86 to 12.36)	1.94 (1.86 to 2.03)	615 (472 to 750)	192.63 (150.67 to 232.79)	1.18 (1.12 to 1.24)
Mauritius	392 (340 to 450)	21.07 (18.15 to 24.16)	1.66 (1.49 to 1.82)	248 (224 to 274)	13.61 (12.23 to 15.07)	1.04 (0.90 to 1.19)	6141 (5565 to 6831)	332.67 (301.17 to 369.39)	1.08 (0.94 to 1.21)
Myanmar	8072 (5960 to 10,800)	15.56 (11.46 to 20.74)	1.70 (1.47 to 1.93)	6302 (4606 to 8361)	12.68 (9.27 to 16.66)	1.29 (1.09 to 1.48)	182,039 (133,311 to 241,850)	336.52 (245.88 to 445.02)	1.17 (0.97 to 1.37)
Philippines	14,310 (11,798 to 16,704)	16.04 (13.24 to 18.67)	0.79 (0.68 to 0.89)	10,443 (8699 to 12,031)	12.48 (10.40 to 14.41)	0.59 (0.48 to 0.70)	303,894 (252,876 to 350,650)	321.14 (266.99 to 369.07)	0.56 (0.45 to 0.66)
Seychelles	40 (32 to 50)	31.53 (25.45 to 39.06)	1.01 (0.69 to 1.33)	26 (21 to 32)	21.48 (17.21 to 25.79)	0.45 (0.16 to 0.75)	686 (549 to 843)	533.53 (427.64 to 653.48)	0.42 (0.13 to 0.71)
Sri Lanka	4000 (3152 to 4885)	13.31 (10.53 to 16.21)	1.91 (1.69 to 2.13)	2273 (1851 to 2767)	7.58 (6.18 to 9.21)	0.78 (0.62 to 0.95)	56,424 (46,331 to 68,939)	190.80 (157.54 to 232.62)	0.66 (0.46 to 0.86)

Thailand	30,436 (25,097 to 36,437)	27.28 (22.72 to 32.50)	0.60 (0.26 to 0.95)	17,204 (14,681 to 20,034)	15.15 (13.05 to 17.55)	-0.49 (-0.85 to -0.12)	445,714 (389,335 to 512,786)	413.92 (363.41 to 470.87)	-0.33 (-0.66 to 0.01)
Timor-Leste	121 (88 to 163)	12.86 (9.28 to 17.32)	0.87 (0.73 to 1.02)	94 (69 to 126)	10.44 (7.60 to 14.09)	0.46 (0.32 to 0.59)	2737 (1983 to 3636)	274.46 (197.79 to 366.29)	0.69 (0.50 to 0.89)
Viet Nam	29,010 (20,519 to 38,863)	25.10 (17.86 to 33.77)	3.36 (3.25 to 3.46)	16,110 (11,933 to 21,111)	14.29 (10.61 to 18.82)	2.34 (2.22 to 2.46)	467,372 (340,616 to 615,530)	398.36 (292.40 to 525.42)	2.41 (2.29 to 2.53)

ASIR: age-standardized incidence rate; EAPC: estimated annual percent change; ASDR: age-standardized death rate; ASDALYR: age-standardized disability-adjusted life years rate; UI: uncertainty interval; CI: confidence interval

Table S28. Incidence, death, DALYs, age-standardized rates, and changes from 1990 to 2023 for liver cancer in Asian countries and regions

	2023 Incidence (95% UI)	2023 ASIR Per 100 000 (95% UI)	1990 to 2023 EAPC (95% CI)	2023 Death (95% UI)	2023 ASDR Per 100 000 (95% UI)	1990 to 2023 EAPC (95% CI)	2023 DALYs (95% UI)	2023 ASDALYR Per 100 000 (95% UI)	1990 to 2023 EAPC (95% CI)
**Central Asia**									
Armenia	194 (171 to 219)	3.82 (3.37 to 4.32)	-1.81 (-2.47 to -1.14)	201 (177 to 228)	3.90 (3.45 to 4.39)	-2.15 (-2.89 to -1.40)	4640 (4087 to 5214)	94.95 (83.61 to 106.82)	-2.03 (-2.74 to -1.31)
Azerbaijan	761 (587 to 1042)	6.72 (5.22 to 9.14)	-0.27 (-0.45 to -0.09)	752 (580 to 1025)	6.83 (5.33 to 9.34)	-0.23 (-0.41 to -0.05)	20,675 (16,016 to 27,916)	176.02 (136.96 to 236.50)	-0.54 (-0.72 to -0.36)
Georgia	224 (189 to 258)	3.87 (3.26 to 4.49)	-1.09 (-2.14 to -0.03)	223 (189 to 257)	3.79 (3.20 to 4.38)	-1.07 (-2.19 to 0.06)	5852 (4941 to 6865)	105.69 (88.84 to 124.23)	-0.95 (-2.05 to 0.16)
Kazakhstan	723 (666 to 788)	3.38 (3.11 to 3.68)	-3.72 (-4.13 to -3.31)	709 (654 to 769)	3.39 (3.11 to 3.68)	-3.83 (-4.29 to -3.37)	19,418 (17,891 to 21,045)	88.45 (81.50 to 95.56)	-4.17 (-4.63 to -3.71)
Kyrgyzstan	170 (145 to 196)	3.17 (2.68 to 3.66)	-0.46 (-0.85 to -0.08)	167 (142 to 192)	3.25 (2.75 to 3.74)	-0.12 (-0.50 to 0.27)	4547 (3887 to 5240)	78.75 (67.17 to 90.93)	-0.63 (-1.03 to -0.23)
Mongolia	1688 (1367 to 2063)	67.84 (55.44 to 81.90)	0.72 (0.15 to 1.30)	1662 (1349 to 2031)	70.46 (57.91 to 85.47)	0.90 (0.35 to 1.45)	47,419 (38,466 to 57,919)	1720.82 (1402.36 to 2097.07)	0.28 (-0.24 to 0.81)
Tajikistan	345 (238 to 493)	5.96 (4.08 to 8.53)	-0.15 (-0.43 to 0.13)	338 (233 to 485)	6.22 (4.26 to 8.93)	-0.02 (-0.32 to 0.28)	9855 (6762 to 14,067)	148.62 (102.29 to 212.38)	-0.45 (-0.78 to -0.13)

Turkmenistan	255 (218 to 303)	5.51 (4.71 to 6.49)	0.51 (0.32 to 0.70)	247 (211 to 293)	5.50 (4.70 to 6.45)	0.42 (0.13 to 0.71)	7392 (6325 to 8730)	150.52 (128.99 to 177.06)	0.29 (-0.03 to 0.61)
Uzbekistan	1432 (1311 to 1567)	5.16 (4.72 to 5.63)	0.10 (0.02 to 0.18)	1391 (1274 to 1520)	5.22 (4.76 to 5.72)	0.11 (0.03 to 0.19)	40,369 (36,875 to 44,217)	134.19 (122.26 to 147.01)	-0.14 (-0.25 to -0.04)
**East Asia**									
China	165,113 (143,144 to 190,095)	7.52 (6.58 to 8.64)	-1.39 (-1.54 to -1.23)	143,500 (126,372 to 163,697)	6.44 (5.72 to 7.37)	-1.64 (-1.82 to -1.47)	4,055,010 (3,543,092 to 4,675,076)	189.99 (166.57 to 219.32)	-1.85 (-2.02 to -1.68)
Democratic People's Republic of Korea	2880 (1793 to 4301)	8.47 (5.26 to 12.62)	-0.16 (-0.25 to -0.06)	2644 (1657 to 3881)	7.86 (4.89 to 11.46)	-0.28 (-0.37 to -0.19)	81,469 (50,941 to 125,366)	237.69 (148.45 to 366.29)	-0.36 (-0.44 to -0.27)
**High-income Asia Pacific**									
Brunei Darussalam	43 (31 to 55)	9.74 (7.21 to 12.43)	-1.46 (-1.56 to -1.36)	39 (29 to 49)	9.15 (6.92 to 11.65)	-1.64 (-1.76 to -1.51)	1113 (816 to 1432)	233.48 (173.74 to 297.00)	-1.62 (-1.73 to -1.50)
Japan	40,139 (34,066 to 44,945)	9.73 (8.42 to 10.76)	-2.30 (-2.80 to -1.81)	31,373 (26,217 to 34,578)	7.04 (6.12 to 7.64)	-2.53 (-3.08 to -1.97)	492,735 (427,128 to 534,120)	139.57 (126.33 to 148.57)	-3.42 (-3.96 to -2.88)
Republic of Korea	17,998 (14,187 to 23,011)	18.00 (14.29 to 23.00)	-1.54 (-1.84 to -1.23)	13,356 (10,889 to 16,305)	13.25 (10.78 to 16.16)	-2.45 (-2.74 to -2.17)	298,687 (234,401 to 380,445)	307.36 (241.64 to 391.80)	-2.97 (-3.33 to -2.60)
Singapore	644 (551 to 756)	7.00 (5.95 to 8.22)	-0.87 (-1.46 to -0.28)	491 (445 to 537)	5.29 (4.79 to 5.77)	-1.40 (-2.06 to -0.72)	10,710 (9866 to 11,657)	115.16 (106.08 to 125.09)	-1.93 (-2.59 to -1.27)
**South Asia**									
Bangladesh	5655 (3634 to 8119)	4.06 (2.62 to 5.92)	0.72 (0.52 to 0.91)	5318 (3424 to 7675)	3.95 (2.56 to 5.79)	0.66 (0.46 to 0.85)	164,962 (107,142 to 236,523)	111.42 (71.81 to 159.50)	0.41 (0.17 to 0.66)
Bhutan	25 (14 to 41)	3.65 (2.04 to 5.88)	0.45 (0.24 to 0.67)	24 (13 to 39)	3.51 (1.98 to 5.67)	0.31 (0.07 to 0.54)	756 (419 to 1236)	105.12 (58.56 to 170.53)	0.53 (0.29 to 0.77)
India	36,616 (25,380 to 50,436)	2.81 (1.94 to 3.86)	0.85 (0.68 to 1.03)	35,211 (24,261 to 48,729)	2.76 (1.91 to 3.79)	0.79 (0.60 to 0.99)	1,025,110 (704,183 to 1,411,189)	75.63 (52.24 to 104.32)	0.71 (0.52 to 0.90)
Nepal	553 (364 to 828)	2.03 (1.33 to 3.03)	0.82 (0.56 to 1.09)	530 (349 to 800)	1.99 (1.31 to 3.02)	0.75 (0.47 to 1.03)	16,256 (10,597 to 24,271)	57.48 (37.64 to 85.86)	0.92 (0.67 to 1.16)

Pakistan	4516 (3014 to 6299)	2.95 (1.97 to 4.09)	0.76 (0.58 to 0.93)	4239 (2806 to 5915)	2.87 (1.90 to 3.98)	0.62 (0.44 to 0.81)	141,672 (93,857 to 198,771)	85.93 (57.02 to 119.61)	0.74 (0.56 to 0.92)
**Southeast Asia**									
Cambodia	794 (489 to 1235)	5.36 (3.31 to 8.43)	-0.99 (-1.08 to -0.90)	752 (465 to 1180)	5.23 (3.21 to 8.25)	-1.01 (-1.11 to -0.92)	24,043 (14,647 to 36,779)	154.00 (94.53 to 237.78)	-1.45 (-1.57 to -1.34)
Indonesia	15,751 (9283 to 25,429)	5.31 (3.15 to 8.43)	1.09 (0.97 to 1.22)	14,574 (8598 to 23,471)	5.05 (3.00 to 7.98)	0.95 (0.81 to 1.09)	480,244 (282,936 to 780,294)	155.03 (92.35 to 249.07)	1.21 (1.07 to 1.36)
Lao People's Democratic Republic	430 (268 to 627)	7.73 (4.85 to 11.32)	0.92 (0.81 to 1.03)	403 (252 to 589)	7.54 (4.76 to 11.04)	0.80 (0.70 to 0.91)	13,220 (8256 to 19,238)	219.63 (137.21 to 320.68)	0.81 (0.68 to 0.95)
Malaysia	3310 (2673 to 4402)	10.04 (8.28 to 13.14)	-0.11 (-0.30 to 0.07)	3190 (2588 to 4200)	9.89 (8.23 to 12.75)	-0.27 (-0.46 to -0.08)	85,458 (68,780 to 114,239)	250.96 (202.06 to 334.14)	-0.10 (-0.27 to 0.06)
Maldives	24 (17 to 31)	7.71 (5.62 to 10.06)	0.39 (0.24 to 0.53)	23 (17 to 30)	7.91 (5.79 to 10.31)	0.36 (0.21 to 0.51)	585 (420 to 786)	165.17 (120.39 to 217.57)	-0.31 (-0.43 to -0.19)
Mauritius	21 (18 to 24)	1.14 (0.99 to 1.29)	0.09 (-1.40 to 1.60)	20 (18 to 23)	1.10 (0.96 to 1.25)	1.24 (-0.40 to 2.91)	532 (460 to 602)	28.78 (24.84 to 32.54)	1.28 (-0.35 to 2.93)
Myanmar	3436 (1865 to 6424)	6.46 (3.50 to 12.02)	0.96 (0.86 to 1.05)	3279 (1771 to 6119)	6.32 (3.41 to 11.72)	0.96 (0.83 to 1.08)	100,149 (53,536 to 189,194)	180.78 (97.14 to 339.99)	0.80 (0.66 to 0.93)
Philippines	5392 (4263 to 6665)	5.88 (4.66 to 7.28)	-0.39 (-0.47 to -0.31)	5096 (4062 to 6332)	5.77 (4.58 to 7.18)	-0.29 (-0.37 to -0.20)	159,816 (127,078 to 198,605)	163.57 (130.10 to 203.52)	-0.47 (-0.55 to -0.38)
Seychelles	6 (4 to 9)	4.76 (3.48 to 6.62)	-1.63 (-1.82 to -1.44)	6 (4 to 8)	4.60 (3.36 to 6.35)	-1.72 (-1.95 to -1.50)	168 (123 to 236)	127.02 (92.55 to 177.98)	-1.76 (-1.98 to -1.53)
Sri Lanka	933 (772 to 1146)	3.10 (2.57 to 3.81)	-0.06 (-0.37 to 0.25)	891 (744 to 1098)	2.95 (2.47 to 3.60)	-0.24 (-0.59 to 0.10)	22,726 (18,727 to 27,788)	76.86 (63.53 to 93.90)	-0.41 (-0.79 to -0.04)
Thailand	12,747 (9832 to 16,588)	11.53 (8.96 to 14.88)	-1.22 (-1.45 to -1.00)	11,911 (9367 to 15,266)	10.57 (8.35 to 13.53)	-1.34 (-1.60 to -1.09)	333,056 (254,720 to 444,601)	312.35 (242.72 to 412.36)	-1.22 (-1.48 to -0.96)
Timor-Leste	44 (27 to 66)	4.54 (2.77 to 6.93)	-0.16 (-0.28 to -0.05)	42 (25 to 64)	4.47 (2.71 to 6.83)	-0.28 (-0.38 to -0.17)	1280 (782 to 1945)	125.57 (76.73 to 190.99)	-0.12 (-0.27 to 0.03)
Viet Nam	10,883 (7727 to 14,887)	9.32 (6.56 to 12.69)	-1.48 (-1.73 to -1.24)	9897 (6988 to 13,582)	8.55 (5.98 to 11.68)	-1.65 (-1.90 to -1.40)	307,692 (219,722 to 424,349)	258.61 (183.99 to 355.73)	-1.65 (-1.92 to -1.39)

ASIR: age-standardized incidence rate; EAPC: estimated annual percent change; ASDR: age-standardized death rate; ASDALYR: age-standardized disability-adjusted life years rate; UI: uncertainty interval; CI: confidence interval

Table S29. Incidence, death, DALYs, age-standardized rates, and changes from 1990 to 2023 for pancreatic cancer in Asian countries and regions

	2023 Incidence (95% UI)	2023 ASIR Per 100 000 (95% UI)	1990 to 2023 EAPC (95% CI)	2023 Death (95% UI)	2023 ASDR Per 100 000 (95% UI)	1990 to 2023 EAPC (95% CI)	2023 DALYs (95% UI)	2023 ASDALYR Per 100 000 (95% UI)	1990 to 2023 EAPC (95% CI)
**Central Asia**									
Armenia	376 (338 to 425)	7.31 (6.52 to 8.23)	0.67 (0.35 to 1.00)	386 (345 to 437)	7.43 (6.62 to 8.39)	0.66 (0.33 to 0.98)	8606 (7711 to 9753)	173.10 (154.84 to 194.70)	0.53 (0.20 to 0.86)
Azerbaijan	513 (405 to 652)	4.46 (3.51 to 5.70)	1.99 (1.60 to 2.38)	505 (399 to 642)	4.52 (3.56 to 5.82)	2.07 (1.66 to 2.47)	13,596 (10,684 to 17,333)	111.58 (88.06 to 143.20)	1.70 (1.33 to 2.06)
Georgia	401 (356 to 462)	6.65 (5.89 to 7.64)	4.19 (3.63 to 4.75)	408 (361 to 469)	6.68 (5.90 to 7.67)	4.20 (3.64 to 4.76)	9616 (8491 to 11,091)	166.19 (146.91 to 190.80)	4.11 (3.54 to 4.68)
Kazakhstan	1046 (928 to 1148)	4.89 (4.33 to 5.40)	-0.36 (-0.65 to -0.07)	1020 (908 to 1116)	4.87 (4.33 to 5.37)	-0.36 (-0.65 to -0.07)	27,678 (24,663 to 30,356)	125.47 (111.89 to 137.40)	-0.55 (-0.84 to -0.25)
Kyrgyzstan	253 (220 to 289)	4.83 (4.20 to 5.52)	1.69 (1.03 to 2.35)	247 (215 to 281)	4.92 (4.28 to 5.61)	1.75 (1.08 to 2.41)	6771 (5917 to 7675)	117.88 (102.73 to 133.74)	1.44 (0.78 to 2.11)
Mongolia	224 (178 to 269)	8.65 (6.87 to 10.47)	1.34 (1.24 to 1.44)	215 (171 to 259)	8.70 (6.89 to 10.59)	1.33 (1.22 to 1.44)	6361 (5038 to 7664)	223.08 (177.26 to 269.07)	1.26 (1.16 to 1.36)
Tajikistan	124 (94 to 159)	2.15 (1.59 to 2.75)	0.51 (0.07 to 0.95)	122 (92 to 157)	2.21 (1.63 to 2.84)	0.60 (0.17 to 1.03)	3357 (2514 to 4306)	52.02 (38.91 to 66.71)	0.19 (-0.25 to 0.64)
Turkmenistan	129 (111 to 154)	2.85 (2.46 to 3.35)	8.88 (7.67 to 10.10)	125 (107 to 149)	2.84 (2.46 to 3.34)	8.87 (7.66 to 10.09)	3611 (3105 to 4287)	74.82 (64.10 to 88.16)	8.85 (7.61 to 10.10)
Uzbekistan	742 (665 to 827)	2.72 (2.43 to 3.02)	1.90 (1.67 to 2.13)	721 (644 to 802)	2.74 (2.44 to 3.04)	1.90 (1.68 to 2.13)	20,241 (18,046 to 22,546)	68.24 (60.70 to 75.83)	1.79 (1.57 to 2.01)
**East Asia**									
China	118,381 (103,773 to 135,339)	5.13 (4.48 to 5.88)	-0.24 (-0.37 to -0.11)	115,396 (101,499 to 132,269)	4.99 (4.38 to 5.73)	-0.34 (-0.48 to -0.21)	2,775,370 (2,463,011 to 3,148,173)	121.04 (106.88 to 137.26)	-0.59 (-0.73 to -0.46)
Democratic People's Republic of Korea	1520 (1072 to 2061)	4.48 (3.16 to 6.14)	0.95 (0.79 to 1.11)	1483 (1051 to 2032)	4.44 (3.14 to 6.15)	0.90 (0.74 to 1.06)	40,230 (28,492 to 54,352)	115.47 (81.42 to 155.05)	0.84 (0.69 to 1.00)
**High-income Asia Pacific**									

Brunei Darussalam	23 (18 to 28)	5.63 (4.46 to 6.96)	0.46 (0.36 to 0.55)	22 (18 to 27)	5.58 (4.38 to 6.84)	0.39 (0.30 to 0.49)	590 (463 to 724)	129.45 (102.35 to 159.13)	0.35 (0.24 to 0.45)
Japan	56,868 (46,849 to 63,174)	13.60 (11.85 to 14.76)	0.98 (0.86 to 1.10)	50,283 (41,626 to 55,455)	11.88 (10.39 to 12.86)	0.75 (0.62 to 0.87)	825,971 (720,228 to 893,224)	244.85 (222.33 to 260.94)	0.53 (0.43 to 0.63)
Republic of Korea	10,068 (8447 to 11,356)	9.85 (8.26 to 11.09)	-0.03 (-0.17 to 0.11)	8914 (7609 to 9880)	8.72 (7.45 to 9.66)	-0.44 (-0.58 to -0.31)	175,673 (154,795 to 195,432)	174.76 (153.89 to 194.24)	-1.07 (-1.24 to -0.89)
Singapore	657 (585 to 728)	7.07 (6.29 to 7.85)	0.21 (0.11 to 0.31)	590 (533 to 641)	6.32 (5.69 to 6.86)	-0.14 (-0.24 to -0.03)	12,365 (11,324 to 13,401)	131.55 (120.68 to 142.30)	-0.47 (-0.58 to -0.35)
**South Asia**									
Bangladesh	3200 (2310 to 4280)	2.42 (1.75 to 3.22)	1.57 (1.33 to 1.82)	3094 (2229 to 4122)	2.43 (1.74 to 3.25)	1.57 (1.35 to 1.80)	86,463 (62,771 to 116,348)	61.14 (44.17 to 81.80)	1.36 (1.07 to 1.66)
Bhutan	12 (8 to 17)	1.85 (1.27 to 2.56)	1.33 (1.22 to 1.43)	12 (8 to 17)	1.84 (1.26 to 2.53)	1.23 (1.12 to 1.34)	332 (225 to 457)	47.98 (32.63 to 65.89)	1.42 (1.30 to 1.53)
India	23,021 (17,439 to 29,096)	1.84 (1.39 to 2.32)	1.24 (1.06 to 1.41)	22,766 (17,133 to 28,642)	1.86 (1.40 to 2.35)	1.22 (1.05 to 1.38)	591,786 (452,819 to 744,201)	45.10 (34.36 to 56.52)	1.12 (0.93 to 1.31)
Nepal	339 (241 to 453)	1.30 (0.92 to 1.72)	0.72 (0.42 to 1.02)	336 (238 to 445)	1.31 (0.94 to 1.73)	0.68 (0.38 to 0.98)	8928 (6423 to 11,952)	32.72 (23.44 to 43.91)	0.74 (0.45 to 1.03)
Pakistan	3442 (2457 to 4474)	2.44 (1.75 to 3.18)	1.41 (1.28 to 1.54)	3358 (2399 to 4368)	2.46 (1.76 to 3.24)	1.33 (1.21 to 1.46)	94,823 (67,883 to 123,937)	63.14 (45.18 to 82.25)	1.47 (1.34 to 1.60)
**Southeast Asia**									
Cambodia	331 (235 to 439)	2.34 (1.66 to 3.07)	1.65 (1.49 to 1.80)	326 (231 to 429)	2.36 (1.68 to 3.11)	1.70 (1.54 to 1.86)	9144 (6557 to 12,174)	61.15 (43.63 to 81.11)	1.33 (1.18 to 1.49)
Indonesia	9722 (7089 to 12,594)	3.38 (2.49 to 4.36)	2.01 (1.84 to 2.19)	9229 (6741 to 11,933)	3.31 (2.42 to 4.27)	1.89 (1.71 to 2.06)	283,718 (206,395 to 371,807)	93.31 (67.90 to 121.18)	2.18 (2.00 to 2.35)
Lao People's Democratic Republic	200 (140 to 255)	3.80 (2.67 to 4.84)	2.87 (2.69 to 3.05)	193 (135 to 246)	3.82 (2.67 to 4.88)	2.80 (2.63 to 2.98)	5715 (3968 to 7312)	100.67 (70.40 to 128.47)	2.88 (2.68 to 3.07)
Malaysia	1486 (1254 to 1757)	4.62 (3.88 to 5.46)	1.10 (1.00 to 1.21)	1486 (1248 to 1752)	4.72 (3.97 to 5.58)	1.00 (0.88 to 1.11)	37,254 (31,266 to 44,189)	110.98 (93.53 to 131.57)	1.14 (1.06 to 1.23)
Maldives	11 (9 to 14)	3.98 (3.13 to 4.91)	2.36 (2.26 to 2.46)	11 (9 to 14)	4.22 (3.33 to 5.19)	2.44 (2.34 to 2.54)	256 (202 to 312)	80.81 (62.99 to 98.66)	1.71 (1.61 to 1.82)

Mauritius	97 (86 to 109)	5.15 (4.55 to 5.80)	0.21 (-0.13 to 0.56)	97 (86 to 108)	5.17 (4.58 to 5.80)	0.14 (-0.20 to 0.49)	2408 (2128 to 2705)	127.80 (113.03 to 143.56)	0.20 (-0.14 to 0.54)
Myanmar	1963 (1419 to 2550)	3.82 (2.75 to 4.97)	2.00 (1.79 to 2.21)	1932 (1392 to 2513)	3.86 (2.76 to 5.03)	1.98 (1.78 to 2.19)	53,369 (38,461 to 69,422)	98.90 (71.41 to 128.46)	1.88 (1.67 to 2.08)
Philippines	2951 (2456 to 3417)	3.38 (2.82 to 3.92)	1.02 (0.92 to 1.12)	2887 (2404 to 3340)	3.44 (2.86 to 3.97)	1.01 (0.91 to 1.11)	80,755 (66,903 to 94,434)	86.16 (71.61 to 100.42)	0.91 (0.82 to 1.01)
Seychelles	8 (7 to 11)	6.56 (5.28 to 8.24)	0.43 (0.27 to 0.58)	8 (7 to 10)	6.59 (5.30 to 8.27)	0.41 (0.25 to 0.56)	217 (174 to 273)	165.55 (132.74 to 208.27)	0.33 (0.19 to 0.48)
Sri Lanka	860 (733 to 1017)	2.84 (2.41 to 3.35)	1.78 (1.63 to 1.94)	854 (731 to 1014)	2.82 (2.40 to 3.35)	1.72 (1.58 to 1.86)	20,990 (17,947 to 24,934)	70.11 (59.81 to 83.57)	1.60 (1.42 to 1.78)
Thailand	5522 (4638 to 6573)	4.87 (4.11 to 5.76)	-0.15 (-0.52 to 0.22)	5442 (4551 to 6470)	4.74 (4.00 to 5.60)	-0.28 (-0.66 to 0.10)	139,485 (115,734 to 165,439)	126.55 (106.72 to 148.93)	-0.13 (-0.49 to 0.22)
Timor-Leste	23 (16 to 32)	2.54 (1.71 to 3.53)	0.85 (0.66 to 1.04)	23 (16 to 32)	2.58 (1.71 to 3.59)	0.75 (0.57 to 0.93)	629 (433 to 851)	65.21 (44.58 to 88.92)	0.96 (0.74 to 1.18)
Viet Nam	3689 (2653 to 4850)	3.24 (2.34 to 4.26)	2.56 (2.46 to 2.67)	3588 (2571 to 4726)	3.19 (2.29 to 4.19)	2.44 (2.34 to 2.55)	97,483 (70,017 to 128,761)	83.14 (59.73 to 109.74)	2.60 (2.50 to 2.71)

ASIR: age-standardized incidence rate; EAPC: estimated annual percent change; ASDR: age-standardized death rate; ASDALYR: age-standardized disability-adjusted life years rate; UI: uncertainty interval; CI: confidence interval

Table S30. Incidence, death, DALYs, age-standardized rates, and changes from 1990 to 2023 for gallbladder and biliary tract cancer in Asian countries and regions

	2023 Incidence (95% UI)	2023 ASIR Per 100 000 (95% UI)	1990 to 2023 EAPC (95% CI)	2023 Death (95% UI)	2023 ASDR Per 100 000 (95% UI)	1990 to 2023 EAPC (95% CI)	2023 DALYs (95% UI)	2023 ASDALYR Per 100 000 (95% UI)	1990 to 2023 EAPC (95% CI)
**Central Asia**									
Armenia	50 (43 to 57)	0.94 (0.82 to 1.07)	1.34 (0.68 to 2.01)	51 (44 to 58)	0.96 (0.83 to 1.10)	1.28 (0.61 to 1.96)	1129 (979 to 1287)	21.96 (18.99 to 24.99)	1.13 (0.50 to 1.76)
Azerbaijan	71 (52 to 96)	0.63 (0.46 to 0.85)	-0.78 (-0.94 to -0.63)	71 (52 to 95)	0.65 (0.47 to 0.88)	-0.74 (-0.88 to -0.59)	1828 (1327 to 2422)	15.25 (11.04 to 20.29)	-1.05 (-1.22 to -0.89)
Georgia	91 (79 to 104)	1.48 (1.27 to 1.69)	-1.22 (-2.17 to -0.25)	93 (81 to 107)	1.50 (1.30 to 1.71)	-1.27 (-2.25 to -0.28)	2120 (1833 to 2424)	35.44 (30.66 to 40.69)	-1.39 (-2.33 to -0.45)
Kazakhstan	170 (149 to 192)	0.81 (0.72 to 0.92)	-1.68 (-1.92 to -1.43)	167 (148 to 189)	0.82 (0.73 to 0.93)	-1.75 (-2.01 to -1.48)	4353 (3853 to 4940)	20.02 (17.70 to 22.72)	-1.81 (-2.05 to -1.57)

Kyrgyzstan	42 (36 to 48)	0.82 (0.71 to 0.95)	-2.72 (-3.75 to -1.69)	41 (36 to 47)	0.85 (0.73 to 0.98)	-2.70 (-3.72 to -1.67)	1072 (932 to 1229)	19.41 (16.82 to 22.17)	-3.01 (-4.05 to -1.95)
Mongolia	50 (38 to 67)	1.97 (1.48 to 2.69)	-0.99 (-1.15 to -0.83)	48 (37 to 66)	2.01 (1.51 to 2.77)	-0.99 (-1.14 to -0.83)	1373 (1041 to 1818)	49.65 (37.53 to 67.25)	-1.16 (-1.33 to -0.99)
Tajikistan	9 (7 to 13)	0.16 (0.12 to 0.23)	-0.36 (-0.76 to 0.05)	9 (7 to 13)	0.17 (0.12 to 0.24)	-0.30 (-0.69 to 0.09)	246 (176 to 333)	3.93 (2.82 to 5.37)	-0.60 (-1.02 to -0.19)
Turkmenistan	33 (28 to 39)	0.75 (0.64 to 0.87)	-4.61 (-5.85 to -3.35)	33 (28 to 38)	0.76 (0.65 to 0.88)	-4.63 (-5.87 to -3.38)	900 (770 to 1047)	19.08 (16.37 to 22.16)	-4.65 (-5.88 to -3.40)
Uzbekistan	93 (82 to 106)	0.35 (0.31 to 0.40)	1.46 (1.02 to 1.90)	92 (81 to 104)	0.36 (0.32 to 0.41)	1.39 (0.96 to 1.83)	2444 (2144 to 2781)	8.52 (7.52 to 9.66)	1.45 (1.01 to 1.89)
**East Asia**									
China	45,688 (35,602 to 60,313)	1.99 (1.55 to 2.64)	-0.61 (-0.74 to -0.48)	33,946 (27,355 to 44,923)	1.48 (1.19 to 1.96)	-1.58 (-1.76 to -1.40)	752,766 (615,798 to 998,842)	32.73 (26.83 to 43.49)	-1.82 (-1.99 to -1.64)
Democratic People's Republic of Korea	647 (390 to 1320)	1.95 (1.18 to 4.05)	0.38 (0.23 to 0.54)	596 (352 to 1221)	1.84 (1.10 to 3.80)	0.15 (0.02 to 0.29)	15,031 (9047 to 29,809)	43.85 (26.40 to 87.95)	0.15 (0.02 to 0.28)
**High-income Asia Pacific**									
Brunei Darussalam	9 (7 to 11)	2.28 (1.74 to 2.95)	-1.32 (-1.38 to -1.25)	9 (7 to 11)	2.37 (1.80 to 3.09)	-1.38 (-1.44 to -1.32)	206 (159 to 261)	48.62 (37.24 to 62.35)	-1.49 (-1.56 to -1.42)
Japan	33,176 (26,472 to 37,874)	6.81 (5.72 to 7.67)	-1.19 (-1.24 to -1.13)	24,274 (19,438 to 27,533)	4.79 (4.03 to 5.35)	-1.90 (-1.96 to -1.84)	340,058 (285,351 to 379,583)	83.56 (73.43 to 91.53)	-2.34 (-2.41 to -2.27)
Republic of Korea	9013 (7097 to 11,480)	8.82 (6.96 to 11.24)	-0.12 (-0.22 to -0.03)	6718 (5425 to 8372)	6.57 (5.29 to 8.19)	-1.35 (-1.49 to -1.20)	117,759 (97,374 to 151,717)	115.72 (95.56 to 150.42)	-1.94 (-2.09 to -1.80)
Singapore	137 (113 to 164)	1.48 (1.23 to 1.77)	-1.27 (-1.43 to -1.11)	104 (88 to 120)	1.12 (0.94 to 1.30)	-2.26 (-2.44 to -2.08)	1948 (1665 to 2243)	20.79 (17.75 to 23.96)	-2.57 (-2.75 to -2.39)
**South Asia**									
Bangladesh	5054 (3544 to 7014)	3.90 (2.73 to 5.38)	1.23 (1.04 to 1.41)	4913 (3427 to 6807)	3.97 (2.76 to 5.48)	1.22 (1.06 to 1.39)	134,370 (93,941 to 185,389)	96.38 (67.38 to 133.14)	0.99 (0.77 to 1.21)
Bhutan	19 (13 to 25)	2.80 (1.94 to 3.84)	0.96 (0.79 to 1.13)	18 (13 to 25)	2.84 (1.97 to 3.89)	0.84 (0.66 to 1.01)	494 (338 to 682)	71.68 (49.52 to 98.96)	1.03 (0.86 to 1.21)
India	32,501 (23,554 to 43,570)	2.63 (1.89 to 3.53)	1.14 (1.00 to 1.27)	32,444 (23,559 to 43,598)	2.71 (1.95 to 3.63)	1.10 (0.95 to 1.24)	823,206 (596,199 to 1,104,253)	63.46 (45.93 to 85.12)	1.01 (0.88 to 1.14)

Nepal	534 (377 to 738)	2.08 (1.46 to 2.88)	0.20 (-0.12 to 0.51)	539 (381 to 746)	2.16 (1.52 to 2.99)	0.17 (-0.14 to 0.48)	13,736 (9803 to 18,838)	51.14 (36.38 to 70.27)	0.14 (-0.17 to 0.46)
Pakistan	3304 (2320 to 4540)	2.39 (1.67 to 3.28)	0.27 (0.12 to 0.43)	3291 (2306 to 4500)	2.47 (1.74 to 3.37)	0.22 (0.06 to 0.37)	89,849 (63,097 to 123,120)	60.70 (42.80 to 83.27)	0.32 (0.17 to 0.48)
**Southeast Asia**									
Cambodia	84 (53 to 166)	0.60 (0.37 to 1.21)	-0.02 (-0.18 to 0.13)	82 (51 to 164)	0.61 (0.38 to 1.23)	-0.03 (-0.18 to 0.12)	2219 (1392 to 4243)	15.09 (9.46 to 29.34)	-0.30 (-0.45 to -0.15)
Indonesia	2163 (1322 to 4332)	0.76 (0.47 to 1.54)	0.70 (0.50 to 0.90)	2015 (1227 to 4083)	0.74 (0.45 to 1.49)	0.52 (0.33 to 0.71)	60,219 (36,796 to 118,274)	20.07 (12.29 to 40.00)	0.82 (0.62 to 1.01)
Lao People's Democratic Republic	49 (31 to 98)	0.96 (0.59 to 1.94)	1.10 (0.89 to 1.31)	47 (29 to 96)	0.96 (0.58 to 1.96)	0.99 (0.79 to 1.19)	1354 (858 to 2650)	24.39 (15.30 to 48.81)	1.11 (0.89 to 1.33)
Malaysia	310 (238 to 402)	0.97 (0.74 to 1.27)	-1.51 (-1.85 to -1.18)	294 (228 to 385)	0.95 (0.73 to 1.25)	-1.76 (-2.11 to -1.42)	7033 (5443 to 9168)	21.21 (16.46 to 27.63)	-1.64 (-1.96 to -1.31)
Maldives	4 (3 to 5)	1.36 (1.01 to 1.77)	1.23 (1.12 to 1.35)	3 (3 to 4)	1.32 (0.99 to 1.71)	1.00 (0.89 to 1.11)	73 (55 to 92)	24.48 (18.25 to 31.25)	0.27 (0.19 to 0.35)
Mauritius	18 (15 to 21)	0.95 (0.82 to 1.10)	-3.16 (-4.44 to -1.87)	17 (15 to 19)	0.91 (0.79 to 1.04)	-3.33 (-4.61 to -2.04)	390 (342 to 441)	20.55 (17.98 to 23.22)	-3.36 (-4.62 to -2.07)
Myanmar	478 (293 to 924)	0.94 (0.58 to 1.84)	0.08 (-0.18 to 0.34)	468 (286 to 915)	0.95 (0.58 to 1.87)	0.01 (-0.25 to 0.26)	12,486 (7751 to 23,811)	23.42 (14.49 to 44.81)	-0.04 (-0.30 to 0.22)
Philippines	458 (355 to 661)	0.53 (0.41 to 0.77)	0.27 (0.18 to 0.37)	438 (336 to 637)	0.53 (0.40 to 0.77)	0.23 (0.14 to 0.33)	12,060 (9343 to 16,875)	12.99 (10.03 to 18.56)	0.19 (0.10 to 0.27)
Seychelles	1 (1 to 2)	1.02 (0.77 to 1.46)	-2.11 (-2.37 to -1.85)	1 (1 to 2)	0.99 (0.74 to 1.41)	-2.25 (-2.50 to -2.00)	30 (22 to 42)	22.79 (17.09 to 32.57)	-2.31 (-2.56 to -2.05)
Sri Lanka	422 (298 to 719)	1.38 (0.99 to 2.35)	-3.88 (-4.64 to -3.10)	381 (272 to 653)	1.25 (0.90 to 2.13)	-4.23 (-4.98 to -3.48)	8841 (6358 to 15,007)	29.18 (21.21 to 49.29)	-4.28 (-5.06 to -3.50)
Thailand	9571 (7520 to 12,845)	8.25 (6.50 to 11.04)	-0.49 (-0.76 to -0.22)	8535 (6783 to 11,442)	7.28 (5.78 to 9.74)	-0.90 (-1.17 to -0.62)	206,370 (162,017 to 275,479)	179.52 (141.03 to 238.63)	-0.79 (-1.06 to -0.51)
Timor-Leste	7 (4 to 13)	0.76 (0.47 to 1.46)	-0.41 (-0.63 to -0.19)	7 (4 to 13)	0.76 (0.47 to 1.48)	-0.54 (-0.75 to -0.33)	177 (112 to 332)	18.62 (11.68 to 35.27)	-0.33 (-0.57 to -0.08)
Viet Nam	1502 (1080 to 2082)	1.35 (0.98 to 1.87)	0.91 (0.79 to 1.02)	1352 (982 to 1876)	1.24 (0.90 to 1.70)	0.58 (0.48 to 0.69)	34,595 (24,837 to 47,475)	30.12 (21.81 to 41.54)	0.68 (0.58 to 0.78)

ASIR: age-standardized incidence rate; EAPC: estimated annual percent change; ASDR: age-standardized death rate; ASDALYR: age-standardized disability-adjusted life years rate; UI: uncertainty interval; CI: confidence interval
